# The seven human coronavirus main proteases exhibit differential modulation of NF-κB suppression via TAB1 cleavage

**DOI:** 10.1038/s42003-026-10225-9

**Published:** 2026-05-21

**Authors:** Xinming Fu, Dan Cao, Huan Zhou, Hao Huang

**Affiliations:** 1 Laboratory of Structural Biology and Drug Discovery, Laboratory of Ubiquitination and Targeted Therapy, School of Chemical Biology and Biotechnology, Peking University Shenzhen Graduate School, Shenzhen, China; 2 Shanghai Advanced Research Institute, Chinese Academy of Sciences, Shanghai, China; 3 Institute of Chemical Biology, Shenzhen Bay Laboratory, Shenzhen, China

**Keywords:** Biochemistry, Structural biology

## Abstract

The divergence in pathogenicity among human coronaviruses (HCoVs), from causing common cold to lethal pneumonia, may indicate distinct viral immune evasion strategies. However, the molecular basis of their differential immune evasion strategies remains unclear. In this study, we find that the innate immune adapter protein TGF-β activated kinase 1 (TAB1) is a universal target of the main proteases (Mpros) of all seven HCoVs. We show that Mpros from coronaviruses that cause severe disease (SARS-CoV-2, SARS-CoV, and MERS-CoV) efficiently cleave TAB1 at two distinct sites (Q10 and Q444), disrupting the TAB1-TAK1 complex and blocking Nuclear Factor kappa-B (NF-κB) signaling. In contrast, Mpro from common cold coronaviruses (HCoV-OC43, HCoV-HKU1, HCoV-NL63, and HCoV-229E) primarily cleave at Q10, resulting in only partial NF-κB suppression. Furthermore, structural analysis of SARS-CoV-2 Mpro in complex with a TAB1 peptide reveals key features of the substrate-enzyme interaction interface. Kinetic analysis shows that Mpros from the severe disease associated coronaviruses cleave Q444 more efficiently than Mpros from the common cold associated viruses. Conversely, Mpros from common cold associated coronaviruses prefer Q10 over Q444. Although Q10 cleavage is observed in all viruses, Mpros from common cold associated coronaviruses cleave Q10 more efficiently than it cleaves Q444. Overall, our findings establish the Mpro-mediated cleavage of TAB1 as a conserved strategy for immune evasion across HCoVs.

## Abstract (web summary)

This study systematically demonstrates that seven human coronaviruses (HCoVs) suppress the NF-κB pathway by cleaving the key host protein TAB1. Divergent cleavage efficiencies and site preferences underlie their stratified immune evasion strategies, revealing new targets for developing broad-spectrum antiviral therapies.

## Introduction

Coronaviruses are a class of pathogens capable of causing respiratory diseases ranging from mild common colds to lethal pneumonia^1,2^. The COVID-19 pandemic, caused by severe acute respiratory syndrome coronavirus 2 (SARS-CoV-2), has resulted in over seven million deaths worldwide according to the World Health Organization data, underscoring the profound socioeconomic and public health impacts of coronavirus outbreaks. Furthermore, the high fatality rates of severe acute respiratory syndrome coronavirus (SARS-CoV; >10%) and Middle East respiratory syndrome coronavirus (MERS-CoV; ~35%) emphasize the persistent threat posed by highly pathogenic coronaviruses^3–7^. To date, seven coronaviruses known to infect humans (HCoVs) have been identified. Among them, SARS-CoV, MERS-CoV, and SARS-CoV-2 can cause severe pneumonia^4,7,8^, multi-organ failure, and high mortality rates, whereas HCoV-229E, -OC43, -NL63, and -HKU1 typically induce only mild upper respiratory tract infections^9^. Elucidating the viral and host factors underlying this divergence in pathogenicity is crucial for pandemic preparedness, the development of broad-spectrum antiviral agents, and the design of universal vaccines^10,11^.

The maturation of coronavirus nonstructural proteins (nsps) relies on self-cleavage mediated by the main protease (Mpro/3CLpro)^12^. As a core hydrolase, Mpro processes 11 conserved sites within viral polyproteins to liberate functional nsps, such as RNA-dependent RNA polymerase (RdRp)^8^, thereby driving the assembly of the viral transcription-replication complex^13^. The strict substrate specificity of Mpro for Leu-Gln ↓ (Ser/Ala/Gly) motifs ( ↓ denotes the scissile bond) and its high conservation among coronaviruses make it an attractive broad-spectrum therapeutic target^14,15^. Beyond its essential role in viral polyprotein processing, accumulating evidence indicates that Mpro also cleaves host innate immune signaling molecules to facilitate viral immune evasion. For instance, Mpro targets proteins such as NF-κB essential modulator (NEMO) and melanoma differentiation-associated gene 5 (MDA5) to suppress type I interferon production^16–19^ and cleaves gasdermin D (GSDMD) to inhibit pyroptosis^20^. Previous studies, including work from the Yann Gambin group, have shown that SARS-CoV-2 Mpro can cleave TAB1 at sites Q132 and Q444^21^. However, a systematic analysis of Mpro-mediated immune evasion across all seven HCoVs, particularly regarding target specificity, cleavage efficiency, and pathogenic implications, is still lacking.

The NF-κB signaling pathway serves as a cornerstone of antiviral innate immunity, coordinating the expression of inflammatory cytokines, chemokines, and interferon-stimulated genes (ISGs) to establish host defense^22,23^. Activation of this pathway critically depends on the assembly of the TAK1-TAB1 complex, in which TAB1 acts as a scaffolding protein that facilitates TAK1 (TGF-β-activated kinase 1) autophosphorylation and recruitment of the IκB kinase (IKK) complex^21,24^. Disruption of this complex impairs NF-κB signaling and provides a critical opportunity for viral immune evasion.

Therefore, this study aims to systematically investigate the site-specific cleavage of TAB1 by Mpro from all seven HCoVs and to elucidate the molecular mechanisms underlying their differential suppression of NF-κB-mediated innate immune responses. Using structural and enzymatic kinetic analyses, we further reveal that the enhanced catalytic efficiency of Mpro from severe disease associated HCoVs (SARS-CoV-2, SARS-CoV, and MERS-CoV) at the critical Q444 site is a key factor driving differential cleavage. Our findings uncover a conserved yet stratified immune evasion strategy mediated by Mpro through site-selective cleavage of TAB1 among HCoVs.

## Results

### SARS-CoV-2 Mpro interacts with and cleaves TAB1

**Fig. 1:**
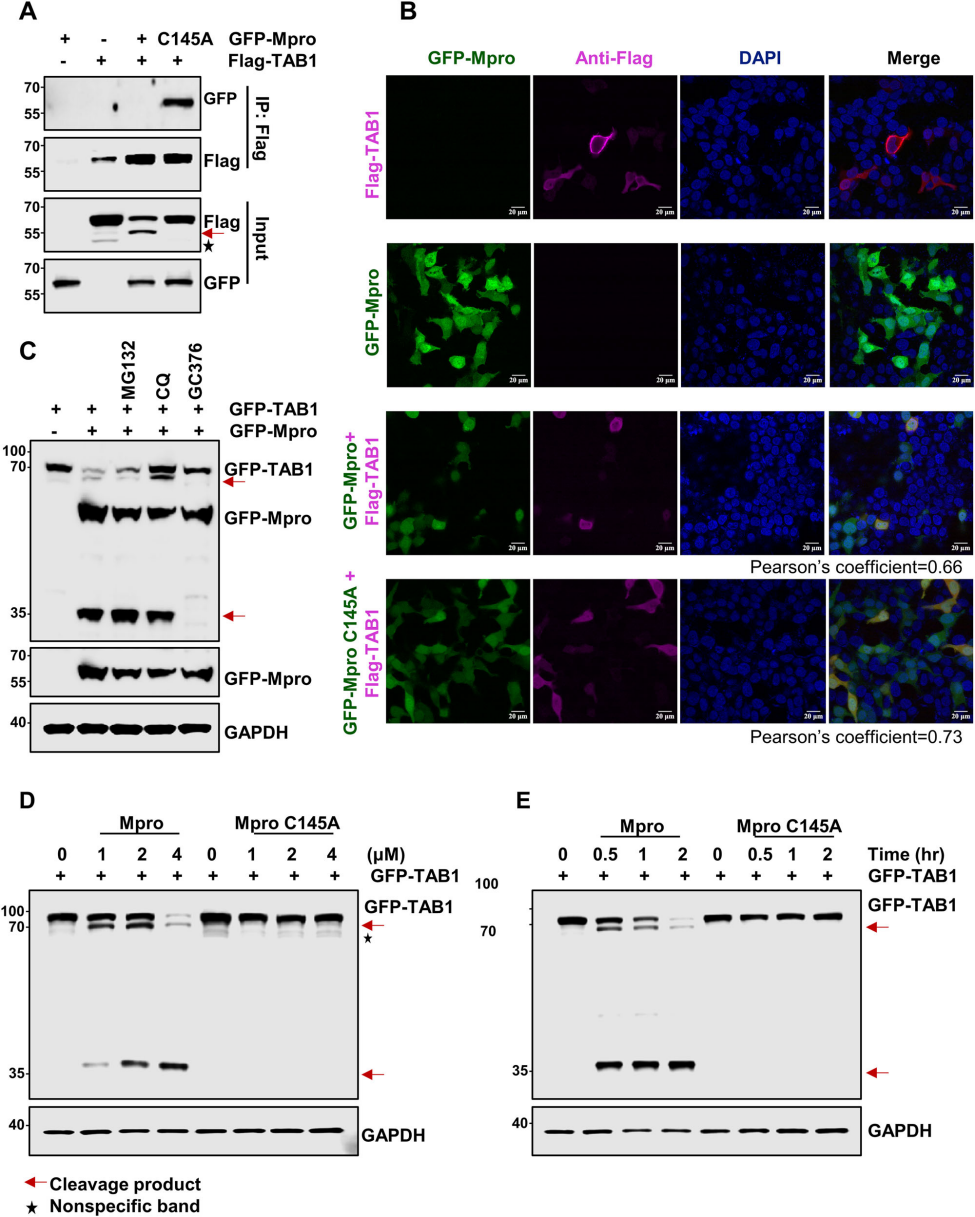
SARS-CoV-2 Mpro interacts with and cleaves TAB1. **A** Co-immunoprecipitation (co-IP) analysis of the interaction between SARS-CoV-2 Mpro and TAB1. HEK-293T cells were co-transfected with plasmids encoding GFP-tagged Mpro (WT or C145A) and Flag-tagged TAB1 for 24 h. Cell lysates were subjected to immunoprecipitation (IP) using anti-Flag M2 magnetic beads, followed by immunoblotting (IB) with antibodies against GFP and Flag. **B** Intracellular colocalization of SARS-CoV-2 Mpro and TAB1. HEK-293T cells transfected with GFP-Mpro (WT- or C145A) and Flag-TAB1 were fixed at 24 h post-transfection. Flag-TAB1 was stained with anti-FLAG primary antibody (1:1000) and Alexa Fluor 594-conjugated secondary antibody (red; 1:2000). Nuclei were counterstained with DAPI (blue). Fluorescence was detected by confocal microscopy (60× oil objective). Single-transfected controls (GFP-Mpro or Flag-TAB1 alone) are shown. Scale bars: 20 μm. **C** Mpro protease activity-dependent cleavage of TAB1 in cells. HEK-293T cells co-expressing GFP-TAB1 and GFP-Mpro were treated for 8 h with proteasome inhibitor MG132 (10 μM), autophagy inhibitor chloroquine (CQ, 20 μM), or Mpro inhibitor GC376 (5 μM). Lysates were analyzed by IB using anti-GFP antibody. **D** Dose-dependent cleavage of TAB1 by SARS-CoV-2 Mpro in vitro. Cell lysates from HEK-293T cells expressing GFP-TAB1 were incubated for 2 h at 37 °C with purified SARS-CoV-2 Mpro (0, 0.5, 1, 2 μM). Cleavage products were detected by anti-GFP IB. **E** Time-course analysis of TAB1 cleavage by SARS-CoV-2 Mpro in vitro. GFP-TAB1-containing lysates were incubated with 1 μM Mpro for 0, 30, 60, or 120 min at 37 °C. Immunoblotting was performed with anti-GFP antibody. GAPDH served as a loading control in all immunoblots (**A**), (**C**), (**D**), and (**E**).

To investigate the interactions between SARS-CoV-2 Mpro and TAB1, we co-expressed N-terminally tagged wild type (WT)-SARS-CoV-2 Mpro (or its catalytically inactive mutant Mpro C145A) and TAB1 in HEK-293T cells. Initial co-immunoprecipitation (Co-IP) assays using anti-Flag M2 beads to pull down TAB1 revealed a specific and robust interaction with the catalytically inactive GFP-Mpro C145A, whereas only a very weak interaction was detected with WT GFP-Mpro (Fig. 1A). This difference in co-precipitation efficiency is consistent with the transient nature of enzyme-substrate interactions, where WT-Mpro cleaves and releases TAB1 during the assay, whereas the inactive C145A mutant forms a more stable complex. Notably, in the input samples, the Flag antibody detected both full-length (FL-) TAB1 and a cleaved fragment (Fig. 1A), confirming the proteolytic activity of WT-Mpro. These results indicate that the interaction between WT Mpro and TAB1 is transient and catalysis-dependent; Mpro likely dissociates from its product after cleavage, explaining its poor recovery in the immunoprecipitate. The residual full-length Flag-TAB1 in the immunoprecipitate may represent the uncut substrate pool. This result qualitatively proves the direct interaction between Mpro and TAB1 and suggests that its catalytic activity significantly affects the stability of the complex. Supporting this, indirect immunofluorescence microscopy showed pronounced cytoplasmic and nuclear colocalization of WT-GFP-Mpro, GFP-Mpro-C145A and Flag-TAB1 (Fig. 1B). We next sought to determine if this interaction leads to TAB1 cleavage. Treatment of cells with the specific Mpro inhibitor GC376, but not with the proteasome inhibitor MG132 or the lysosome inhibitor chloroquine (CQ), markedly inhibited TAB1 cleavage (Fig. 1C), indicating that the proteolytic activity of Mpro is essential. Furthermore, in vitro cleavage assays using lysates from HEK-293T cells expressing GFP-TAB1 confirmed that TAB1 proteolysis increased in both dose- and time-dependent manners in presence of WT-Mpro, but not with Mpro C145A (Fig. 1D, E). Collectively, these results establish that SARS-CoV-2 Mpro directly binds to and cleaves TAB1.

### Identification of Q10 and Q444 as Mpro cleavage sites in TAB1

**Fig. 2:**
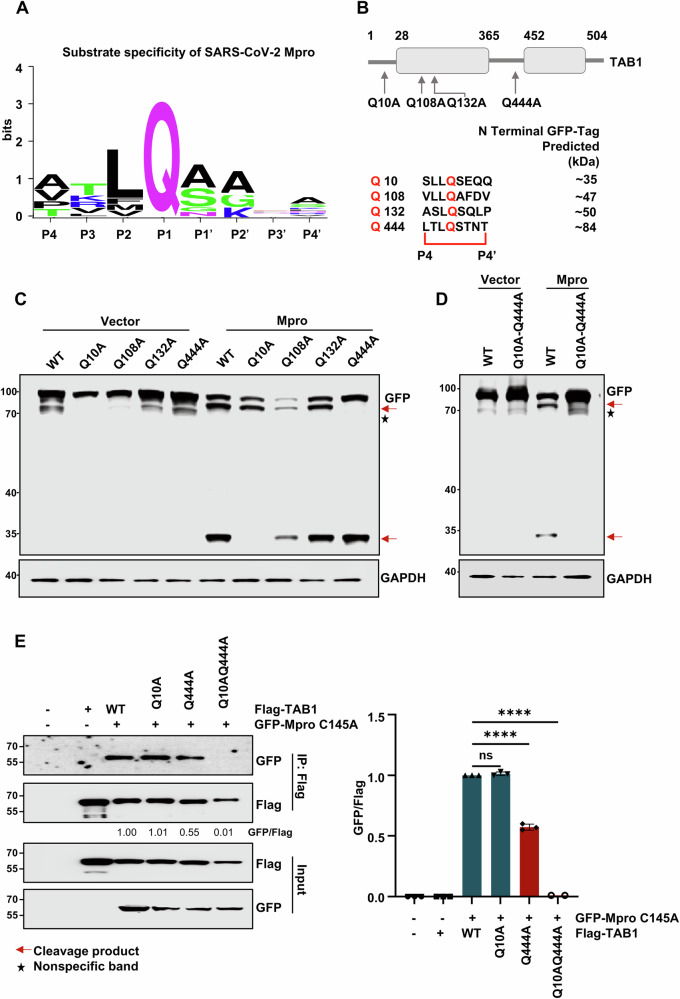
Identification of Q10 and Q444 as SARS-CoV-2 Mpro cleavage sites in TAB1. **A** Substrate specificity of SARS-CoV-2 Mpro. Sequence logo generated by WebLogo 3 (http://weblogo.threeplusone.com/) based on 11 canonical cleavage sites in viral polyproteins (P4-P4’ positions indicated). **B** Domain architecture of human TAB1 and design of cleavage‑site mutants. Schematic of full-length human TAB1 (N-terminal PP2C domain; C-terminal TAK1-binding domain) with Mpro cleavage sites. Site-directed mutants Q10A, Q108A, Q132A and Q444A were constructed. **C** Validation of Q10 as a primary cleavage site. Lysates from HEK-293T cells expressing GFP-tagged TAB1 (WT, Q10A, Q108A, or Q132A) were incubated in vitro with 2 μM purified SARS-CoV-2 Mpro or buffer control for 2 h at 37 °C. Cleavage was assessed by anti-GFP immunoblotting. **D** Essential role of Q10 and Q444 in complete TAB1 processing. Lysates from cells expressing GFP-TAB1 (WT or Q10A/Q444A double mutant) were treated with 2 μM Mpro as in (**C**). Immunoblotting shows complete loss of cleavage in the double mutant. GAPDH served as loading control (**C**, **D**). **E** Interaction of TAB1 mutants with Mpro C145A. Co-IP (anti-Flag) of lysates from HEK-293T cells co-transfected with GFP-Mpro C145A, Flag-TAB1(Q10A, Q444A, Q10A-Q444A), followed by western blot analysis. * Data are presented as mean ± SD of three independent experiments, each performed in triplicate. Statistical significance was determined by one-way ANOVA and is indicated as follows: *P < 0.05, **P < 0.01, ***P < 0.001, ****P < 0.0001; ns, not significant.

Based on the consensus cleavage motif of SARS-CoV-2 Mpro, which exhibits a strong preference for glutamine (Q) at the P1 position (Fig. 2A), we scanned the TAB1 sequence for matching sites. This analysis, combined with the sizes of observed cleavage fragments, identified four candidate sites: Q10, Q108, Q132, and Q444 (Fig. 2B). To validate these sites functionally, we generated a series of TAB1 mutants with glutamine-to-alanine substitutions (Q10A, Q108A, Q132A, Q444A). In cleavage assays where lysates from HEK-293T cells expressing wild-type (WT) or mutant TAB1 were incubated with purified SARS-CoV-2 Mpro, the WT, Q108A, and Q132A proteins all yielded two characteristic fragments: a high molecular weight (MW) band ( ~ 70-80 kDa) and a low-MW band ( ~ 30-35 kDa) (Fig. 2C).

Strikingly, mutation of the candidate sites yielded distinct cleavage patterns. The TAB1 Q10A mutant produced only the high-MW fragment, with a complete absence of the low-MW band. Conversely, the TAB1 Q444A mutant yielded only the low-MW fragment (Fig. 2C). These results indicate that Q10 and Q444 are essential for generating the low- and high-MW fragments, respectively. We then constructed a double mutant (TAB1 Q10A-Q444A), which was completely resistant to Mpro-mediated cleavage (Fig. 2D), confirming that both Q10 and Q444 are bona fide cleavage sites. Multiple sequence alignment revealed high conservation of both Q10 and Q444 among mammalian TAB1 orthologs (Supplementary Table 1), suggesting their functional importance across species^21^.

To investigate whether these cleavage sites are also involved in Mpro recognition of TAB1, we performed co-IP. Flag-WT-TAB1 and point mutants (Q10A, Q444A, and Q10A-Q444A) were tested for interaction with GFP-Mpro C145A. The Q10A mutant retained binding to Mpro C145A, while the Q444A mutant exhibited a markedly reduced binding. Moreover, the Q10A/Q444A double mutant failed to co-immunoprecipitate with Mpro C145A, indicating a complete loss of binding (Fig. 2E). These data establish Q10 and Q444 as critical residues on TAB1 for both recognition and cleavage by SARS-CoV-2 Mpro.

Notably, our identification of Q10 as a major cleavage site differs slightly from a previous study which reported Q132. Since mutation of Q132 did not abolish cleavage in our hands (Fig. 2C), the discrepancy likely stems from differences in experimental systems or detection methods.

In summary, our data demonstrate that SARS-CoV-2 Mpro selectively cleaves TAB1 at two evolutionarily conserved sites (Q10 and Q444), which are also critical for protease binding. This cleavage is likely to dysregulate host signaling pathways such as NF-κB.

### SARS-CoV-2 Mpro suppresses NF-κB signaling through proteolytic inactivation of TAB1

**Fig. 3:**
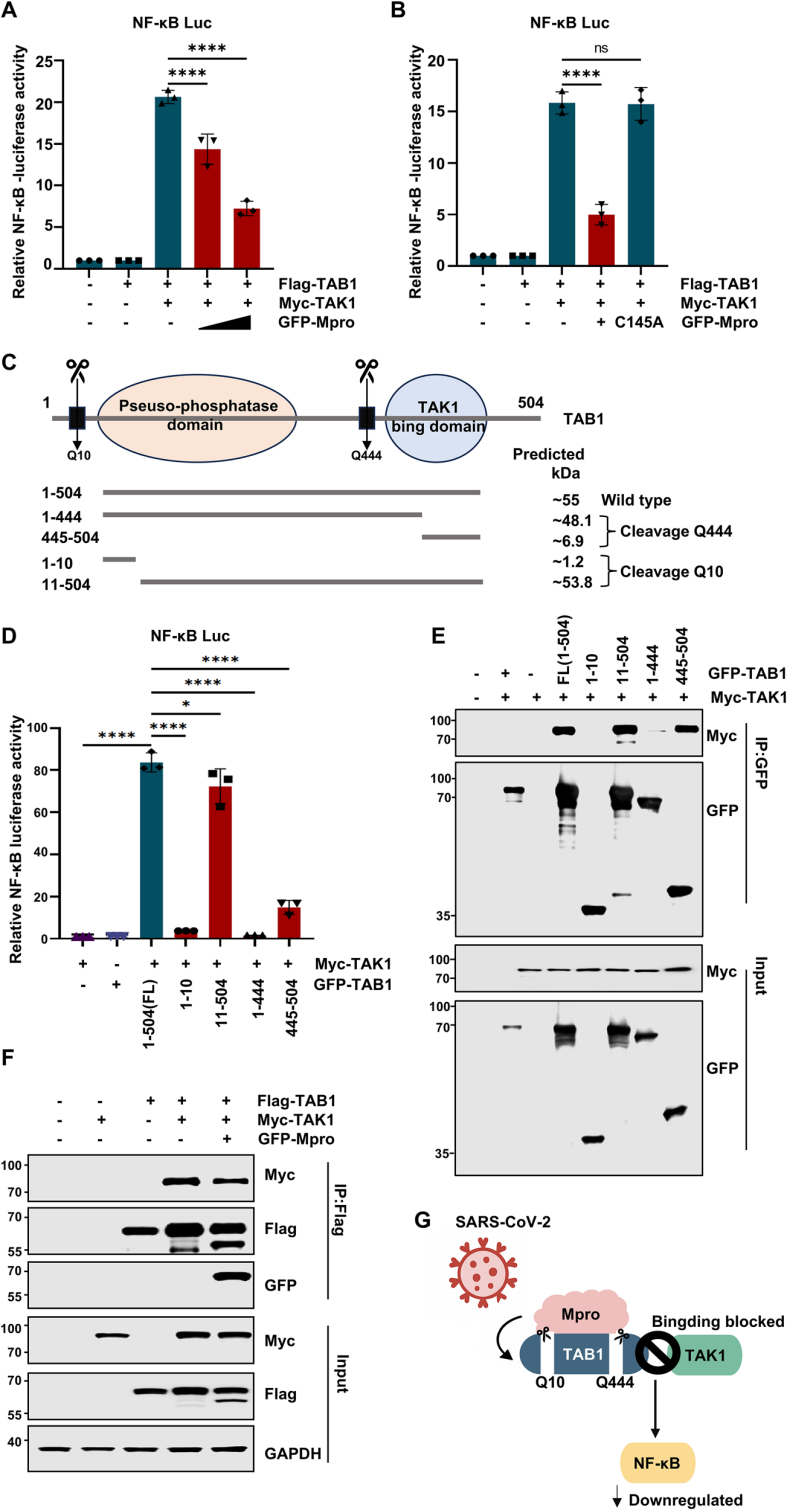
SARS-CoV-2 Mpro suppresses NF-κB signaling by cleaving TAB1. **A** Mpro dose-dependently inhibits TAB1/TAK1-induced NF-κB activation. HEK-293T cells were co-transfected with Flag-TAB1, Myc-TAK1, NF-κB reporter plasmids, and increasing amounts of GFP-Mpro. NF-κB activity was assessed by dual-luciferase assay 24 h post-transfection. **B** Mpro catalytic activity is required for NF-κB inhibition. HEK-293T cells co-transfected with Flag-TAB1, Myc-TAK1, NF-κB reporter plasmids, and either GFP-Mpro or the GFP-Mpro C145A were analyzed by dual-luciferase assay. **C** Schematic of Mpro cleavage sites in human TAB1 ( ↓ indicates scissile bond). **D** Truncated TAB1 mutants show impaired ability to activate NF-κB. HEK-293T cells transfected with NF-κB reporter plasmids and TAB1 truncation mutants (TAB1, TAB1_1-10_, TAB1_11-504_, TAB1_1-444_ or TAB1_445-504_) were analyzed by dual-luciferase assay. **E** Interaction between TAK1 and TAB1 truncation variants. HEK-293 T cells were co-transfected with plasmids expressing Myc-tagged TAK1 and GFP-tagged full-length (FL) or truncated TAB1 (residues 1-10, 11-504, 1-444, or 445-504). After 24 h, cells were harvested and lysed. The lysates were subjected to immunoprecipitation overnight with anti-GFP magnetic antibody. The beads were washed three times, and bound proteins were eluted and analyzed by western blotting using anti-GFP and anti-Myc antibodies. **F** Mpro disrupts TAB1-TAK1 binding. Co-IP (anti-Flag) of lysates from HEK-293T cells co-transfected with GFP-Mpro, Flag-TAB1, and Myc-TAK1, followed by western blot analysis. **G** Model of Mpro-mediated disruption of the TAB1-TAK1 complex. * Data are presented as mean ± SD of three independent experiments, each performed in triplicate. Statistical significance was determined by one-way ANOVA and is indicated as follows: *P < 0.05, **P < 0.01, ***P < 0.001, ****P < 0.0001; ns, not significant.

Using TNF-α to activate NF-κB signaling^25,26^, we observed that co-expression of SARS-CoV-2 Mpro suppressed this activation in a dose-dependent manner, while the catalytically inactive C145A mutant did not (Supplementary Fig. 1A). Given the established role of the TAB1-TAK1 complex in NF-κB signaling^27,28^, we further tested whether Mpro could inhibit TAB1/TAK1‑driven activation. Indeed, SARS‑CoV‑2 Mpro dose‑dependently suppressed NF‑κB activation induced by TAB1-TAK1 overexpression (Fig. 3A), and this suppression was abolished when the C145A mutant was used (Fig. 3B), confirming the requirement for proteolytic activity. We also examined the nuclear translocation of the NF-κB p65 subunit^29^ as a functional readout. The results showed that SARS-CoV-2 Mpro inhibited the TNF-α-induced nuclear translocation of p65, whereas C145A did not (Supplementary Fig. 1B).

To evaluate the functional consequences of Mpro-mediated TAB1 cleavage on innate immune signaling, we examined the NF-κB-inducing capacity of four cleavage fragments corresponding to the predicted products: TAB1(1-10), TAB1(11-504), TAB1(1-444), and TAB1(445-504) (Fig. 3C), following transfection into HEK-293T cells. Notably, only the TAB1(11-504) fragment retained significant ability to activate NF-κB, while TAB1(1-10), TAB1(1-444), and TAB1(445-504) failed to elicit robust signaling (Fig. 3D). These results indicate that cleavage at Q10 liberates an N-terminal decapeptide but leaves a large C-terminal fragment (11-504) that remains functionally active. Cleavage at Q444, however, is critical for signaling inactivation, as it serves the essential C-terminal TAK1-binding domain, generating two signaling-deficient fragments.

We next assessed the impact of these TAB1 truncations on the TAB1-TAK1 interaction. Co-IP assays confirmed that, consistent with a previous study^27^, the C-terminal region of TAB1 (present in TAB1(445-504) and the intact protein) retained binding capacity for TAK1, whereas the N-terminal fragments (TAB1(1-10) and TAB1(1-444)) did not (Fig. 3E). This explains the signaling incompetence of the TAB1(1-444) fragment observed in Fig. 3D.

We therefore hypothesized that SARS‑CoV‑2 Mpro disrupts the TAB1-TAK1 complex by cleaving TAB1 and removing its critical C‑terminal binding region. To test this hypothesis, we performed co‑IP experiments. As shown in Fig. 3F, a robust TAB1-TAK1 interaction was observed in the absence of Mpro, whereas co‑expression of WT‑Mpro markedly attenuated this binding. Notably, Mpro C145A did not disrupt the interaction, confirming that the observed effect is due to proteolytic activity. It is worth noting that the use of an N-terminally tagged TAB1 may overestimate the reduction in TAK1 binding due to the concurrent cleavage at the Q10 site. However, as demonstrated in Fig. 3E, the C-terminal region of TAB1 (445-504) is essential for TAK1 binding. Thus, the loss of the C-terminus by Mpro cleavage is sufficient to abolish the TAB1-TAK1 interaction entirely, regardless of the tag separation at the N-terminus. Together, these data demonstrate that Mpro‑mediated cleavage of TAB1 directly disrupts the TAB1-TAK1 complex.

In summary, SARS‑CoV‑2 Mpro cleaves TAB1 at two key sites: cleavage at Q10 releases a small N‑terminal peptide that leaves the core signaling fragment largely intact, whereas cleavage at the critical Q444 site removes the essential C‑terminal TAK1‑binding domain, thereby disrupting the TAB1-TAK1 association and effectively blocking downstream NF‑κB signaling (Fig. 3G).

### Seven human coronavirus Mpros cleave TAB1 at different sites

Next, we investigated whether TAB1 cleavage extends beyond SARS-CoV-2 to other human coronaviruses (HCoVs). We performed a comparative analysis of these seven HCoV Mpros from the perspectives of phylogenetic relationships (Supplementary Fig. 2A), sequence identity (Supplementary Fig. 2B), and key biochemical properties (Supplementary Fig. 2C). Multiple sequence alignment confirmed low overall sequence identity among them (Supplementary Fig. 3), yet their predicted catalytic domains exhibited high structural similarity (Supplementary Fig. 4).

**Fig. 4:**
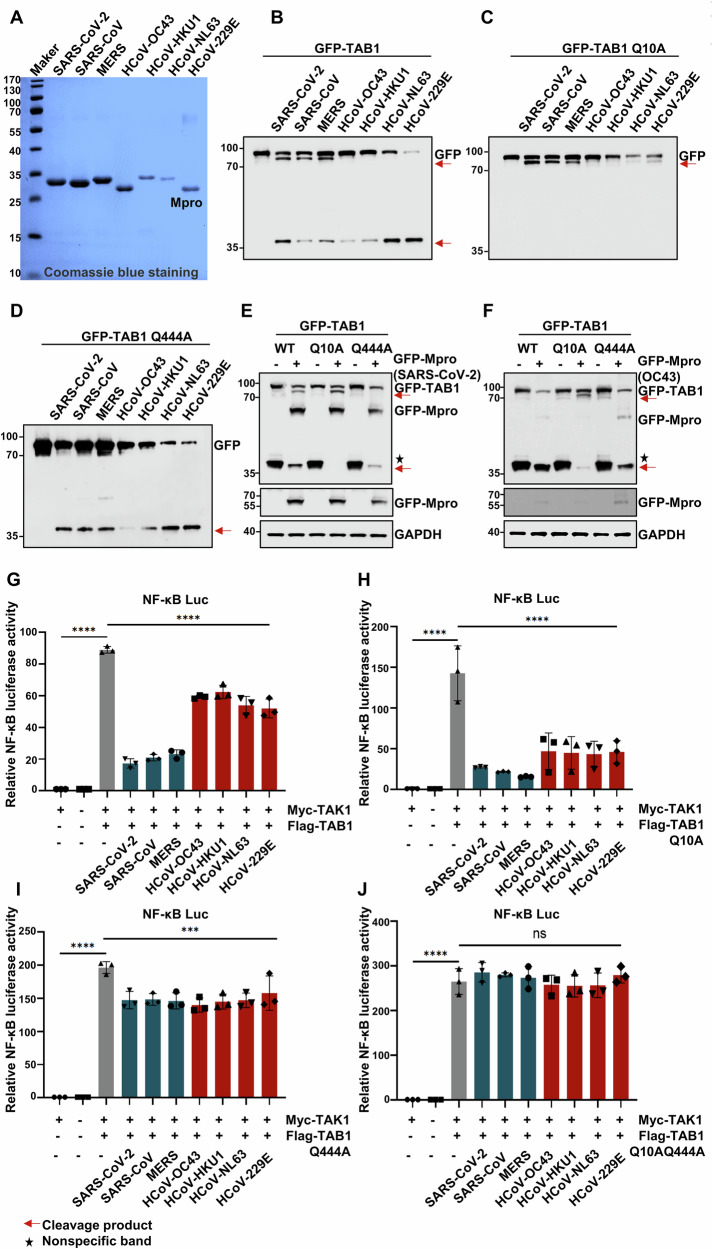
Cleavage of TAB1 by seven human coronavirus Mpro proteases at multiple sites. **A** Purification of WT-Mpros from seven human coronaviruses, validated by SDS-PAGE and Coomassie blue staining. **B–D** In vitro cleavage of TAB1 by Mpros. HEK-293T cells were transfected for 24 h with plasmids encoding: **B** GFP-TAB1, **C** GFP-TAB1 Q10A mutant, or **D** GFP-TAB1 Q444A mutant. Cells were lysed on ice for 30 min, and lysates were incubated with Mpro for 2 h at 37 °C. Immunoblots show GFP-TAB1 levels; GAPDH served as a loading control. **E** HCoV-OC43 Mpro-mediated cleavage of TAB1. HEK-293T cells were co-transfected with GFP-TAB1 and GFP-Mpro plasmids for 24 h, followed by lysis (30 min, ice). Immunoblots detect GFP-TAB1; GAPDH is a loading control. **F** SARS-CoV-2 Mpro cleavage of TAB1. Experimental procedure and analysis identical to (**E**). **G–J** Inhibition of TAB1(Q10A, Q444A, Q10A-Q444A)-TAK1-induced NF-κB activation by seven coronavirus Mpros. HEK-293T cells were co-transfected with GFP-Mpro, Flag-TAB1(Q10A, Q444A, Q10A-Q444A), Myc-TAK1, and NF-κB reporter plasmids for 24 h. NF-κB activity was assessed by dual-luciferase assay. *Data are presented as mean ± SD of three independent experiments, each performed in triplicate. Statistical significance was determined by one-way ANOVA and is indicated as follows: *P < 0.05, **P < 0.01, ***P < 0.001, ****P < 0.0001; ns, not significant.

We first expressed and purified all seven recombinant HCoV Mpro proteins (Fig. 4A) and assessed their ability to cleave TAB1 in vitro. All seven Mpros cleaved TAB1 but produced distinct fragment patterns (Fig. 4B). Mpros from SARS-CoV-2, SARS-CoV, and MERS-CoV cleaved at both the Q10 and Q444 sites, generating the characteristic doublet of high- and low-molecular-weight (MW) fragments. In contrast, Mpros from the common cold-associated viruses selectively cleaved only at the Q10 site, yielding a single low-MW fragment (Fig. 4B).

To further define the cleavage site specificity, we performed cleavage assays using the TAB1-Q10A and TAB1-Q444A single mutants. When the preferred Q10 site was mutated (Q10A), the Mpros from common cold associated viruses gained the ability to cleave at the Q444 site, producing a high-MW fragment pattern similar to that seen with the SARS/MERS group Mpros (Fig. 4C). Conversely, mutation of the Q444 site (Q444A) did not prevent any of the seven Mpros from cleaving at the Q10 site, resulting in a uniform low-MW fragment pattern (Fig. 4D). These results indicate that while all seven Mpros can recognize the Q10 site, the Mpros from SARS-CoV-2, SARS-CoV, and MERS-CoV possess robust dual-site activity, whereas the common cold-associated virus Mpros exhibit a strong preference for Q10, only cleaving at Q444 when Q10 is unavailable.

We validated these observations by directly comparing the cleavage kinetics of a representative common cold-associated virus Mpro (HCoV-OC43) and a coronavirus that causes severe disease Mpro (SARS-CoV-2); this comparison confirmed the differential site preference and efficiency (Fig. 4E, F).

Given our earlier finding that cleavage at Q444 is critical for NF-κB pathway suppression, we quantitatively compared the inhibitory potency of all seven HCoV Mpros on TAB1/TAK1-induced NF-κB signaling using a luciferase reporter assay. Consistent with their cleavage profiles, Mpros from SARS-CoV-2, SARS-CoV, and MERS-CoV potently inhibited NF-κB activation. In contrast, Mpros from the four common cold associated viruses showed significantly weaker inhibition (*p* < 0.01, Fig. 4G), correlating with their inability to efficiently cleave the Q444 site under normal conditions.

To directly link the cleavage sites to the functional output, we tested the inhibitory effects of all seven Mpros on NF-κB activation induced by the TAB1 mutants (Q10A, Q444A, and Q10A/Q444A) (Fig. 4H–J). All Mpros effectively inhibited signaling driven by the Q10A mutant (which retains the Q444 site). However, their inhibitory capacity was markedly and consistently attenuated against the Q444A mutant (which retains only the Q10 site). Finally, signaling induced by the cleavage-resistant Q10A/Q444A double mutant was largely refractory to inhibition by any of the Mpros. These results demonstrate that cleavage at either site can modulate NF-κB signaling, but efficient cleavage at the Q444 site is the predominant mechanism for strong pathway suppression by HCoV Mpros.

### Enzymatic basis for differential cleavage at Q444 and Q10

To elucidate the molecular basis for the differential cleavage of the TAB1 Q444 and Q10 sites by seven Mpros, we performed in vitro steady-state kinetic analyses. Substrate peptides encompassing the P4-P4’ residues surrounding the Q444 (TAB1 residues 441-448) or Q10 (TAB1 residues 7-14) scissile bonds were synthesized. The catalytic efficiency (*k*_cat_/*K*_m_) of each purified Mpro for the Q444-site peptide varied dramatically across strains (Table 1, Supplementary Fig. 5). Mpros from coronaviruses that cause severe disease (SARS-CoV-2, SARS-CoV, and MERS-CoV) cleaved this peptide with significantly higher efficiency than those Mpros from common cold-associated viruses (HCoV-OC43, -HKU1, -NL63, and -229E). The mean *k*_*cat*_*/K*_*m*_ for the Coronaviruses that cause severe disease Mpro group was approximately 14.4-fold greater than that of the common cold-associated virus Mpro group.

**Table 1:** Kinetics of seven Mpro proteases measured with MCA-TAB1_441-448_-Lys (DNP)

Proteases	*k*_cat_ (min^-1^)	*K*_m_ (μM)	*k*_cat_/*K*_m_ (μM^-1 ^min^-1^)
SARS-CoV-2 Mpro	9.26 ± 0.56	7.03 ± 0.29	1.32 ± 0.05
SARS-CoV Mpro	15.12 ± 1.00	12.61 ± 2.2	1.22 ± 0.18
MERS-CoV Mpro	12.71 ± 0.56	13.81 ± 3.26	0.92 ± 0.12
HCoV-HKU1 Mpro	1.66 ± 0.16	21.64 ± 6.07	0.08 ± 0.02
HCoV-OC43 Mpro	2.72 ± 0.13	25.18 ± 2.38	0.11 ± 0.01
HCoV-NL63 Mpro	1.62 ± 0.13	21.29 ± 1.95	0.08 ± 0.00
HCoV-229E Mpro	2.16 ± 0.20	44.74 ± 6.21	0.05 ± 0.00

In contrast, all seven Mpros cleaved the Q10-site peptide with similar (Table 2, Supplementary Fig. 6), low catalytic efficiencies, which clustered within a narrow range ( ~ 0.20-0.30 µM^-1^s^-1^) (Table 2). Notably, for the four common cold-associated virus Mpros, their efficiency for the Q10 site was slightly higher than their own efficiency for the Q444 site.

**Table 2:** Kinetics of seven Mpro proteases measured with MCA-TAB1_7-14_-Lys (DNP)

Proteases	*k*_cat_ (min^-1^)	*K*_m_ (μM)	*k*_cat_ /*K*_m_ (μM^-1 ^min^-1^)
SARS-CoV-2 Mpro	43.14 ± 0.92	147.10 ± 14.29	0.29 ± 0.02
SARS-CoV Mpro	50.35 ± 4.77	169.80 ± 41.18	0.30 ± 0.04
MERS-CoV Mpro	21.87 ± 1.38	88.46 ± 20.33	0.25 ± 0.04
HCoV-HKU1 Mpro	47.65 ± 2.81	158.50 ± 18.13	0.30 ± 0.02
HCoV-OC43 Mpro	50.55 ± 1.31	165.50 ± 12.12	0.30 ± 0.01
HCoV-NL63 Mpro	48.24 ± 4.33	165.90 ± 12.82	0.29 ± 0.01
HCoV-229E Mpro	20.27 ± 1.33	101.20 ± 3.48	0.20 ± 0.01

These kinetic data directly explain the cleavage patterns observed in cellular assays (Fig. 4B, C). For severe disease-causing Mpros, their strong kinetic preference for the Q444 site dictates exclusive cleavage at this position in WT-TAB1. For common cold-associated Mpros, their relatively higher efficiency for the Q10 site makes it the dominant cleavage target in WT-TAB1, while their weak basal activity at Q444 is kinetically outcompeted. In the Q10A-TAB1 mutant, removal of this dominant site allows the pre-existing, albeit low, activity of common cold strain Mpros at the Q444 site to become detectable over the reaction time course, explaining the appearance of the high-MW fragment (Fig. 4C).

In summary, the differential ability of coronavirus Mpros to suppress NF-κB signaling stems from their distinct intrinsic catalytic efficiencies for the two TAB1 cleavage sites.

### Structural basis for differential catalytic efficiency at the Q444 site

**Fig. 5:**
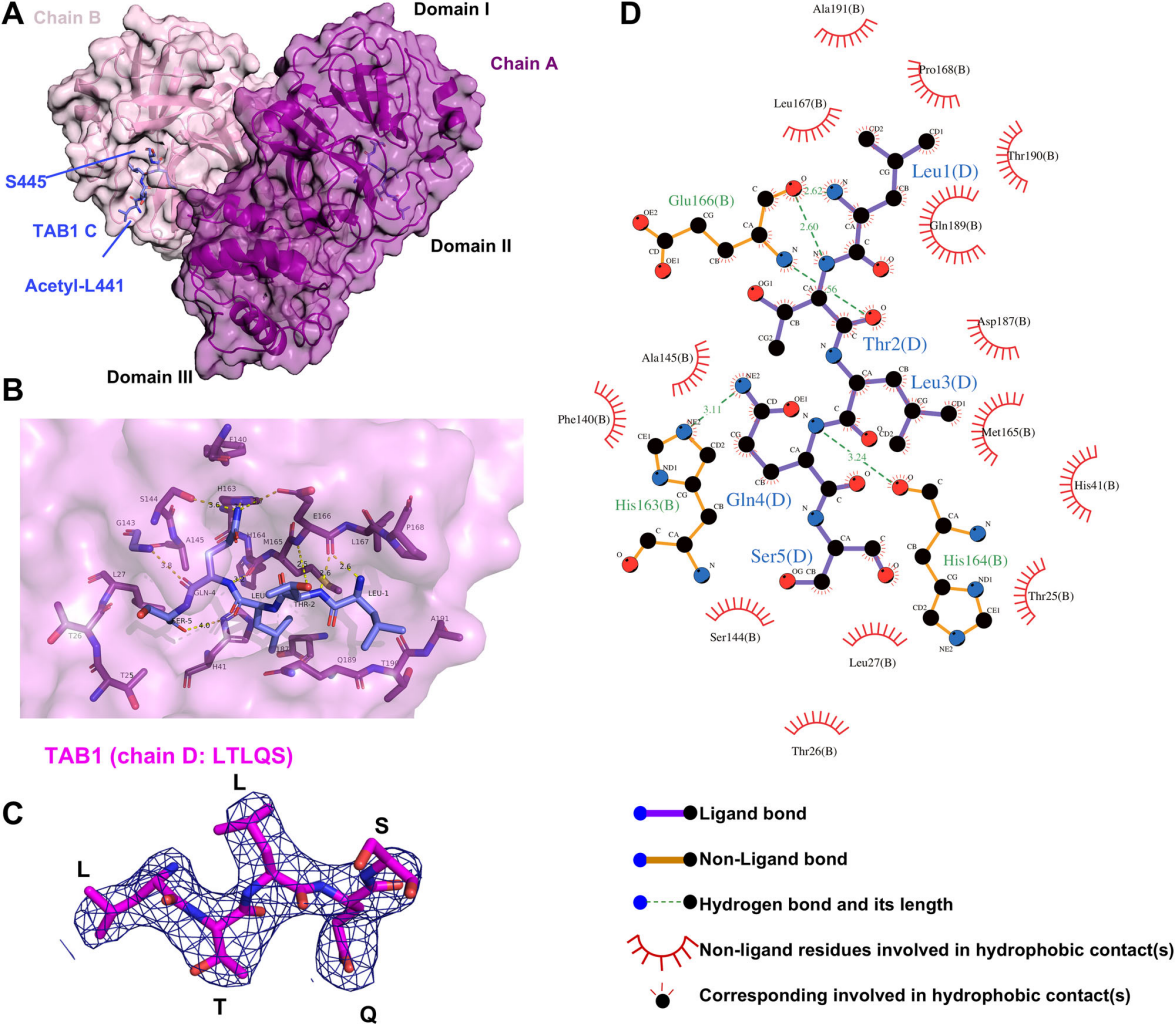
The complex structure of Mpro C145A homodimer bound to TAB1. **A** Overall structure of the Mpro C145A dimer bound to the TAB1 peptide (residues 441-448). The N- and C-termini are labeled in the figure. TAB1_441-448_ (TAB1 -blue) binds to chain B (purple). Lys441 and Tyr445 at the N- and C-termini of the TAB1_441-445_ peptide are labeled. The C-terminal tail of chain D (teal) from a crystallographic symmetry equivalent molecule bind to the substrate-binding site of chain A (light pink). Domains I-III are labeled. **B** Superposition of the co-crystal structures of SARS-CoV-2 Mpro and TAB1 (orange) (LTLQS, cyan, PDB 9U96) in the S4-S1’ pocket. **C** Electron density map for the bound TAB1 peptide. The 2Fo-Fc electron density maps of peptides in the co-crystal structure with Mpro are contoured at 1.0 σ. The structure of TAB1 peptide (LTLQS, PDB 9U96) in complex with Mpro C145A is presented in purple. The electron density maps of residues T(P2’) N(P3’) T (P4’) in TAB1 peptide are not visible **D** Intermolecular interactions of TAB1_441-448_ with SARS-CoV-2 Mpro C145A. The carbon, oxygen, and nitrogen atoms are colored black, red, and blue, respectively. The ligand bonds are shown in purple and Mpro bonds are shown in orange. Hydrogen bonds are represented by green dashed lines. Hydrophobic contacts are presented as radiating lines around the Mpro residues and corresponding ligand atoms. This Figure was prepared using LigPlot.

To elucidate the structural basis for the differential catalytic efficiency of Mpros toward the Q444 site, we determined the crystal structure of the SARS-CoV-2 Mpro C145A mutant in complex with a human TAB1-derived peptide (residues 441-448) at 3.43 Å resolution (Fig. 5A, Table 3). The structure reveals an Mpro homodimer, with one TAB1 peptide bound in the substrate-binding cleft of each protomer. The medium resolution is likely due to the presence of 2% DMSO, which was used to dissolve the peptide for crystallization. Notably, peptide binding induces localized conformational changes in the Mpro active site.

**Table 3:** Statistics of the co-crystal structure for Mpro bound TAB1 (9U96)

PDB entry	Mpro-TAB1_441-445_ (PDB code:9U96)
Data collection	
Space group	C 1 2 1
Cell dimensions	
a, b, c (Å)	123.855, 81.078, 63.651
α, β, γ (°)	90, 90.503, 90
Resolution (Å)	29.87 - 3.43 (3.553 - 3.43)
Unique reflections	8507 (827)
Completeness (%)	95.34 (74.29)
Wilson B-factor	80.29
Reflections used in refinement	8168 (630)
Reflections used for R-free	392 (33)
R-work	0.2173 (0.3725)
R-free	0.2439 (0.3258)
Number of non-hydrogen atoms	4742
macromolecules	4742
ligands	0
solvent	0
Protein residues	615
RMS (bonds)	0.003
RMS (angles)	0.59
Ramachandran favored (%)	95.88
Ramachandran allowed (%)	3.95
Ramachandran outliers (%)	0.16
Rotamer outliers (%)	0.00
Clash score	7.87
Average B-factor	88.03
macromolecules	88.03
Number of TLS groups	1

Analysis of the binding interface reveals that the peptide primarily engages the S4 to S1’ subsites of Mpro (Fig. 5B). Well-defined electron density was observed for the LTLQ ↓ S motif (residues 441-445) of the peptide bound to protomer B, enabling unambiguous modeling (Fig. 5C). The C-terminal residues 446-448 exhibited structural disorder and could not be modeled. The following structural analysis is based on the protomer B complex due to its superior map quality.

A detailed view of the substrate-binding groove shows the atomic interactions stabilizing the Michaelis complex-like configuration (Fig. 5D). The P1 glutamine (Gln444) side chain inserts deeply into the S1 specificity pocket, with its Oε1 atom forming a hydrogen bond with the imidazole Nε2 of His163. The backbone amide nitrogen of Gln444 forms a hydrogen bond with the carbonyl oxygen of Phe140. At the P2 position, the leucine side chain (Leu443) is buried in the hydrophobic S2 pocket, making extensive van der Waals contacts with residues His41, Met49, and Met165. The P3 threonine (Thr442) is stabilized by a hydrogen bond network involving the side chain hydroxyl group and the backbone atoms of Glu166. The P4 leucine (Leu441) occupies the S4 subsite, engaging in hydrophobic interactions with Met165 and Gln189. At the P1’ position, serine (Ser445) is positioned in the S1’ pocket; its side chain hydroxyl group is within hydrogen-bonding distance of the Nε2 atom of His172. The residues at P2’ to P4’ (Thr446-Asn447-Thr448) are located at the periphery of the active site, and their poor electron density precluded precise modeling of their conformations. This structure reveals, at atomic resolution, the canonical mode by which SARS-CoV-2 Mpro recognizes the P4-P4’ segment of its substrate, providing a mechanistic basis for its cleavage specificity.

To understand the structural basis for the differing catalytic efficiencies at the Q444 site, we performed a comparative analysis of the substrate-binding sites of all seven HCoV Mpros, using our SARS-CoV-2 Mpro-TAB1 complex structure as a reference (Supplementary Fig. 7, Supplementary Table 2). While the overall architecture of the substrate-binding cleft is conserved, key variations in residues lining the S2 and S4 subsites distinguish the severe disease-causing Mpros from the common cold-associated coronavirus Mpros. For instance, in SARS-CoV-2 Mpro, the S2 pocket is defined by hydrophobic residues (e.g., His41, Met49) that optimally accommodate the P2 leucine of TAB1. In common cold coronavirus Mpros (e.g., HCoV-229E, HCoV-NL63), this pocket is narrower or contains polar residues, which may disfavor the binding of the hydrophobic P2 side chain. Similarly, variations in the S4 subsite are evident. These structural differences in the substrate-binding environment, particularly those affecting accommodation of the P2 and P4 leucine residues, correlate with the measured kinetic parameters and provide a molecular explanation for the significantly higher catalytic efficiency (*k*_cat _/ *K*_m_) of the highly pathogenic Mpros toward the TAB1 Q444-site peptide.

## Discussion

**Fig. 6:**
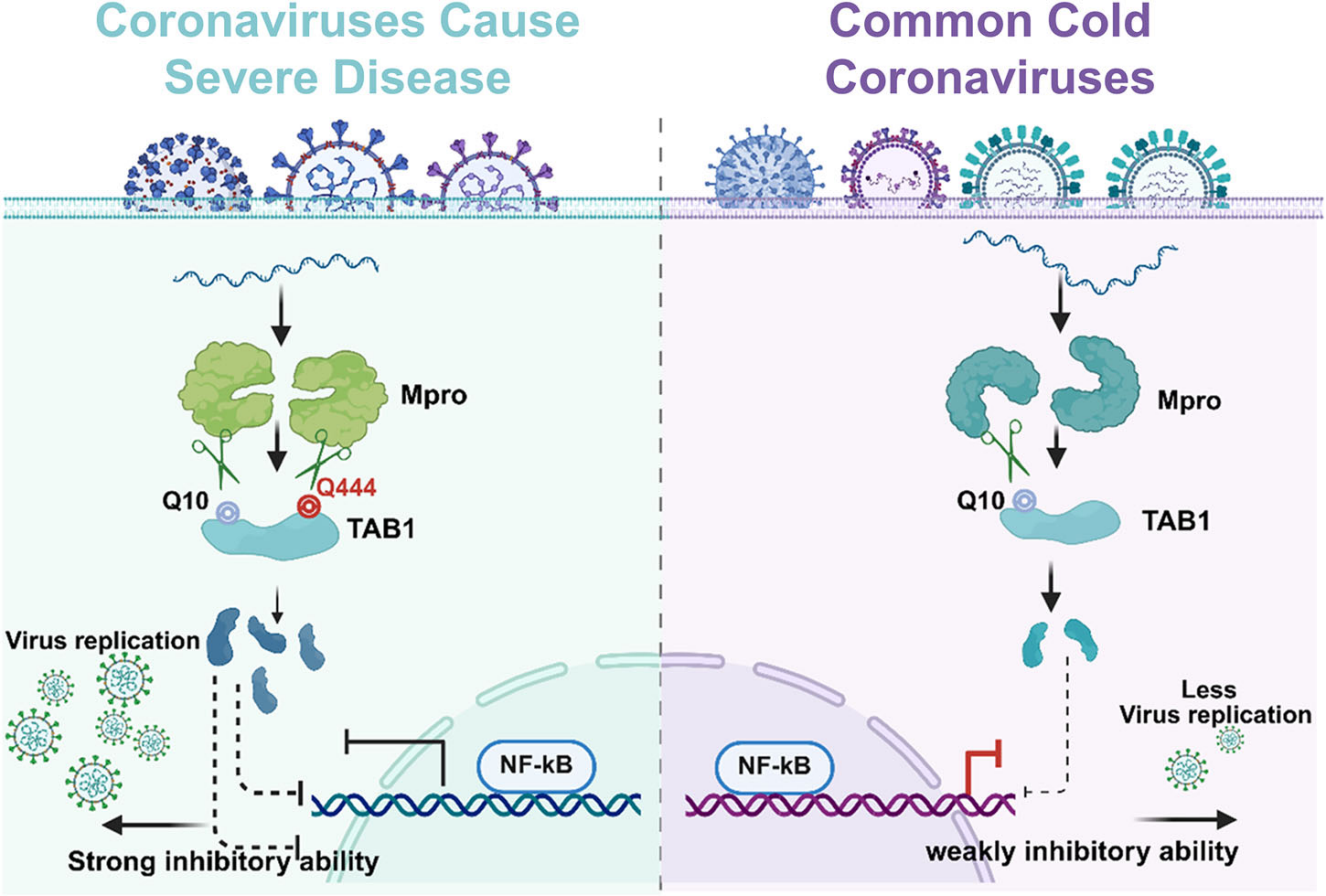
Differential mechanisms of NF‑κB pathway suppression by Mpro‑mediated TAB1 cleavage in human coronaviruses. The schematic summarizes distinct proteolytic strategies employed by Mpro proteases from different human coronaviruses. Left:  For coronaviruses that cause severe disease (SARS-CoV-2, SARS-CoV, MERS-CoV), Mpros cleave TAB1 at both Q10 and Q444 sites. Cleavage at Q444 potently inhibits NF-κB signaling and promotes viral replication. Right:  For common cold-associated coronaviruses. (HCoV-OC43, HCoV-HKU1, HCoV-NL63, HCoV-229E), Mpros primarily cleave TAB1 at Q10. This single site cleavage preserves residual TAB1-TAK1 interaction and NF-κB activation, resulting in only moderate suppression of host signaling. The model highlights Q444 cleavage as a key determinant of strong NF-κB inhibition. Created in BioRender. Fu, X. (2026) https://BioRender.com/t8bjthk.

This study elucidates a conserved yet finely tuned mechanism of immune evasion, wherein the seven human coronavirus Mpros universally cleave the host adapter protein TAB1 to suppress NF-κB signaling (Fig. 6). Mpros from coronaviruses that cause severe disease cleave TAB1 with high efficiency at two sites, Q10 and Q444. The cleavage at Q444 is functionally decisive, as it disrupts the TAB1-TAK1 complex and potently inhibits NF-κB activation. In contrast, Mpros from common cold-associated viruses exhibit a strong preference for the Q10 site, with only a basal, inefficient activity toward Q444. This divergent cleavage selectivity results in a C-terminal TAB1 fragment (residues 11-504) that retains the ability to interact with and activate TAK1.

Mechanistically, the differential cleavage is rooted in the intrinsic catalytic properties of the Mpro enzymes. Detailed enzyme kinetics revealed that Mpros from coronaviruses that cause severe disease cleaves a Q444-site peptide with a catalytic efficiency (*k*_cat _/ *K*_m_) more than an order of magnitude greater than that of their common cold coronaviruses’ counterparts. This stark difference in enzymatic efficiency provides a plausible biochemical basis for the observed phenotypic divergence in immune suppression. Comparative structural analysis suggests that the enhanced Q444-cleaving activity of severe disease-causing Mpros correlates with a more extensive and optimized network of enzyme-substrate interactions.

Our findings refine the existing model of SARS-CoV-2 Mpro substrate specificity, clearly identifying Q10 and Q444 as the primary cleavage sites in TAB1. This work integrates functional virology, enzymology, and structural biology to establish a direct link between Mpro substrate selectivity and catalytic proficiency, it highlights the Mpro-TAB1 interface as a critical nexus in the virus-host conflict and a promising target for the development of broad-spectrum antiviral strategies. Our Mpro-TAB1 complex structure plays a critical role in defining the precise molecular interface and provides a framework for discovering antiviral inhibitors. This mirrors the strategy previously applied to the discovery of drugs targeting the S1’-S3’ pocket^30^ and other pockets^31,32^ of SARS-CoV-2 Mpro. Future studies employing TAB1 cleavage-site knock-in animal models will be crucial to validate the in vivo significance of this mechanism and to explore its therapeutic potential.

## Methods

### Cell culture

HEK293T (ATCC) cells were maintained in Dulbecco’s modified Eagle’s medium (HyClone) supplemented with 10% fetal bovine serum (FBS; Yeasen), 1% penicillin/streptomycin (Basalmedia), 1×non-essential amino acid solution (Basalmedia), and 10 mM HEPES (Solarbio). All cells were cultured at 37 °C in a humidified incubator containing 5% CO_2_.

### Plasmids and transfection

Wild-type (WT) and mutant TAB1 expression constructs were generated for this study. Full-length human TAB1 cDNA was cloned into the pCMV vector with an N-terminal Flag or GFP tag to generate TAB1-WT. Site-directed mutagenesis was performed to generate the single (Q10A, Q108A, Q132A, Q444A) and double (Q10A-Q444A) point mutants. Additionally, a series of truncation mutants were constructed, corresponding to the predicted Mpro cleavage fragments: TAB1(1-10), TAB1(11-504), TAB1(1-444), and TAB1(445-504). For transient transfection, cells at approximately 80% confluence were transfected with plasmid DNA using Hieff Trans Liposomal Transfection Reagent (Yeasen, 40802, Shanghai, China) according to the manufacturer’s protocol.

### Antibodies and reagents

The antibodies used for western blotting included anti-Flag (1:2000, Sigma-Aldrich, F7425; 1:2500, Cell Signaling Technology, 14793), anti-Myc (1:2000, Cell Signaling Technology, 18583), anti-GAPDH (1:10000, Cell Signaling Technology, 5174), anti-rabbit IgG HRP-linked antibody (1:5000, Cell Signaling Technology, 7074), and anti-mouse IgG horseradish peroxidase (HRP)-linked antibody (1:5000, Cell Signaling Technology, 7076), anti-p65(1:2000, Abcam, ab32536), anti-Lamin B1(1:2000, Proteintech, 12987-1-AP).

### Western blot and immunoprecipitation

For western blotting, cells were lysed in lysis buffer (20 mM Tris-HCl (pH 7.4), 150 mM NaCl, 5 mM EDTA, 10% glycerol, 2% Triton X-100, and 0.1% SDS) on ice for 30 min, followed by centrifugation at 15,000 rpm for 10 min. Proteins in the supernatant (20-40 μg) were separated by sodium dodecyl sulfate-polyacrylamide gel electrophoresis (SDS-PAGE; ExpressPlus PAGE Gel, 4-20%, GenScript, M00657) and transferred to a nitrocellulose membrane using the eBlot-L1 system (GenScript, L00686). The membrane was blocked with 5% skim milk in TBST buffer for 1 h and incubated overnight with primary antibodies at 4 °C. Following incubation with horseradish peroxidase (HRP)-conjugated secondary antibodies for 2 h, the proteins were detected using the e-BLOT western blot imager system. Protein levels were quantified from western blot bands using ImageJ software.

For immunoprecipitation, cells were lysed in lysis buffer (50 mM Tris-HCl (pH 7.4), 150 mM NaCl, 1 mM EDTA, and 1% NP-40) by incubation on ice for 30 min, and the lysates were centrifuged at 15,000 rpm for 10 min. The supernatant was incubated with anti-Flag M2 Affinity Gel (Sigma-Aldrich, A2220) and rotated overnight at 4 °C. The beads were washed thrice with lysis buffer, boiled in 2× SDS sample buffer at 95 °C for 5 min, and subjected to SDS-PAGE, followed by western blot analysis.

### Luciferase reporter assays

NF‑κB luciferase reporter plasmids were purchased from YouBio (Changsha, China). For experiments examining the effect of Mpro on TAB1/TAK1‑induced signaling, HEK293T cells were co‑transfected with the NF‑κB reporter plasmid together with plasmids expressing Flag‑TAB1 and Myc‑TAK1 to constitutively activate the pathway. To test the role of Mpro, cells were additionally co‑transfected with either an empty vector control (GFP), WT-Mpro (GFP‑Mpro), or GFP‑Mpro C145A. Twenty‑four hours post‑transfection, cells were harvested and lysed. Luciferase activity in the clarified lysates was measured using the Dual‑Luciferase Reporter Assay System (Promega) according to the manufacturer’s instructions.

HEK-293T cells in six-well plates were co-transfected with GFP- or GFP-Mpro-expressing plasmids. Twenty-four hours post-transfection, the cells were stimulated with TNF-α (10 ng ml^-1^; Sino Biological Inc., China) for 6 h. The cells were lysed, and the luciferase activity of the supernatant was determined using the Dual-Luciferase Reporter Assay System (Promega), according to the manufacturer’s protocol. All cell lysates in this study were assayed by western blotting to detect the expression of transfected proteins. The luciferase activity data were transformed into a fold ratio of the control and statistically analyzed using GraphPad Prism.

### TAB1 cleavage assay

HEK-293T cells were collected, washed with PBS, then lysed in cell lysis buffer (9803, Cell Signaling) on ice for 30 min, the lysates were centrifuged at 17000×*g* for 30 min at 4 °C, and the supernatant was collected. Mpro (2 μM) was co-incubated with cell lysates containing Flag-tagged TAB1, GFP-tagged TAB1 for 2 h at 25 °C. Protein samples were added to 5× loading buffer and heated at 95 °C for 10 min. The samples were then subjected to western blotting analysis.

### SARS-CoV-2 main protease expression and purification

The SARS-CoV-2 Mpro (YP_009742612.1), SARS-CoV Mpro (YP_009944370.1), MERS-CoV Mpro (YP_009047233.1), HCoV-OC43 Mpro (YP_009924323.1), HCoV-HKU1 Mpro (YP_009944273.1), HCoV-NL63 Mpro (AFD98833.1), and HCoV-229E Mpro (AGT21366.1) genes were codon-optimized, synthesized, and subcloned into the pET28a vector with an N-terminal His-sumo-tag (General Biosystems, China). The Mpro C145A mutation was introduced into SARS-CoV-2-Mpro using a QuikChange site-directed mutagenesis kit (Agilent Technologies, U.S.A.) and primers. The plasmids were subsequently transformed into Escherichia coli BL21 (DE3) cells, which were grown in Luria-Bertani (LB) medium. E. coli cells were grown in LB medium with 50 μg/mL kanamycin at 37 °C until the optical density measured at 600 nm (OD600) reached 0.6-1.0, then 0.5 mM Isopropyl β-D-1- thiogalactopyranoside (IPTG) was added, and cells were grown overnight at 17 °C. Next, the cells were centrifuged at 8000×g for 10 min, and the cell pellets were resuspended in buffer A (50 mM HEPES, 500 mM NaCl, 20 mM imidazole, pH 8.0) with the addition of 1 mM phenylmethylsulphonyl fluoride (PMSF) and lysed by sonication. The cell lysate was centrifuged at 40,000×g and 4 °C for 40 min. The supernatant was loaded onto a HisTrap FF column (GE Healthcare) and washed with buffer A. The tagged protease was eluted with buffer B (50 mM HEPES, 450 mM NaCl, 500 mM imidazole, pH 7.5) and digested with ubiquitin-like specific protease 1 (ULP1) to remove the tag. The reaction mixture was passed through a Ni-affinity column to remove undigested protease. The digested protein was run on a Superdex 200 column equilibrated with buffer C (50 mM HEPES and 150 mM NaCl, pH 7.4). The fractions were pooled, concentrated to 35 mg/mL, and stored at -80 °C.

### Multiple sequence alignment of seven Mpros

All Mpro protein sequences from the seven human coronaviruses were obtained from the NCBI website (www.ncbi.nlm.nih.gov/). Multiple sequence alignments of the Mpro proteins were performed using MUSCLE. Figures were generated using the ESPript3.

### Kinetic analysis of seven HCoVs Mpros

Steady-state kinetic parameters (*K*_m_ and *k*_cat_) for the seven HCoV Mpros were determined using fluorogenic peptide substrates corresponding to the TAB1 Q444 and Q10 cleavage sites: MCA-LTLQ ↓ TNT-DNP and MCA-SLLQ ↓ SEQQ-DNP (synthesized by GenScript), respectively. Cleavage at the scissile bond (↓) releases the fluorescent 7-methoxycoumarin-4-acetate (MCA) group. Assays were performed at 25 °C in reaction buffer containing 50 mM HEPES (pH 7.4), 150 mM NaCl, 5 mM DTT, 0.1 mg/mL bovine serum albumin (BSA), and 0.01% (v/v) Triton X-100. Reactions (30 μL final volume) were initiated by adding enzyme, and fluorescence increase (excitation 480 nm, emission 535 nm) was monitored every 2 min for 20-30 min using an EnVision 2104 multilabel plate reader (PerkinElmer). Initial velocities (*v*_0_, μM·min^-1^) were calculated from the linear slope of fluorescence versus time, converted using a standard curve generated from complete hydrolysis of each substrate. Substrate saturation curves were obtained by plotting *v*_0_ against substrate concentration and fitted to the Michaelis-Menten equation: *v*_0_ = (*k*_cat_ [E] [S]) / (*K*_m_ + [S]). where [E] is the total enzyme concentration, [S] is the substrate concentration, *k*_cat_ is the catalytic turnover number, and Km is the Michaelis constant. The catalytic efficiency is expressed as *k*_cat_ / *K*_m_. All measurements were performed in triplicate. Kinetic parameters are reported as mean ± SEM (*n* = 3) and were analyzed using GraphPad Prism 8.0.

### Co-Crystallization of SARS-CoV-2 Mpro C145A with a TAB1-derived peptide

For co-crystallization, freshly purified SARS-CoV-2 Mpro C145A (20 mg/mL) was mixed with a synthetic TAB1 peptide (residues 441-448) at a 1:5 molar ratio (enzyme: peptide). The peptide was first dissolved in dimethyl sulfoxide (DMSO) and then added to the protein solution. The mixture was incubated at 4 °C for 2 h, followed by centrifugation at 16,000 × g for 10 min to remove any precipitate. Crystallization trials were performed using the PACT premier HT-96 screening kit (Molecular Dimensions) with the sitting-drop vapor-diffusion method at 20 °C. Drops were prepared by mixing 0.75 μL of protein-peptide complex with 0.75 μL of reservoir solution and equilibrated against 50 μL of reservoir solution. Crystals suitable for X-ray diffraction grew in a condition containing 0.2 M magnesium formate dihydrate and 20% (w/v) PEG 3350, pH 6.5.

### Collection of diffraction data and structure determination

Crystals were harvested from the drops and cryoprotected with the mother liquor containing various concentrations of glycerol (10-20%). All diffraction data were collected at the BL10U2 beamline of the Shanghai Synchrotron Radiation Facility (SSRF). The diffraction data were indexed, integrated, and scaled using XDS. The collected crystals were flash-cooled in liquid nitrogen. The structures were solved by molecular replacement, using the apo-Mpro structure (PDB: 6Y2E)^13^ as the searching model. The MOLREP software in CCP4 was used for molecular replacements. The structures were refined in PHENIX with a manual model building using Coot. Structural alignments and graphical representations were generated using PyMOL software.

### Statistics and reproducibility

All statistical analyses were performed using GraphPad Prism version 9.5.1 software. Data are presented as mean ± SD of three independent experiments, each performed in triplicate. Statistical significance was determined by one-way ANOVA and is indicated as follows: *P < 0.05, **P < 0.01, ***P < 0.001, ****P < 0.0001; ns, not significant

### Reporting summary

Further information on research design is available in the Nature Portfolio Reporting Summary linked to this article.

## Supplementary information

Supplementary Information

Description of Additional Supplementary files

Supplementary Data 1

Reporting Summary

Transparent Peer Review file

## Data Availability

The coordinates of the Mpro-TAB1_441-448_ complex were deposited in the RCSB Protein Data Bank (www.rcsb.org) with accession number 9U96, respectively. Uncropped blots and gels for all the experiments presented in the manuscript can be found in Supplementary Fig. 8. Source data can be found in Supplementary Data 1. All other data are available from the corresponding author upon reasonable request.
