# Supplementary material for: The seven human coronavirus main proteases exhibit differential modulation of NF-κB suppression via TAB1 cleavage
Source: Commun Biol. 2026 May 21;9:1087. doi: 10.1038/s42003-026-10225-9 (PMC13462985; doi:10.1038/s42003-026-10225-9)
Supplement: Supplementary file 1 [file 42003_2026_10225_MOESM1_ESM.pdf]

Supplementary Information

The seven human coronavirus main proteases exhibit differential modulation of NF-κB suppression via TAB1 cleavage

Authors: Xinming Fu[1], Dan Cao[1], Huan Zhou[2*], Hao Huang[1,3]*

Affiliations

[1]State Key Laboratory of Natural and Biomimetic Drugs, Laboratory of Structural Biology and Drug Discovery, Laboratory of Ubiquitination and Targeted Therapy, School of Chemical Biology and Biotechnology, Peking University Shenzhen Graduate School, Shenzhen, Guangdong 518055, China.

[2]Shanghai Advanced Research Institute, Chinese Academy of Sciences, Shanghai, China.

[3]Institute of Chemical Biology, Shenzhen Bay Laboratory, Shenzhen, Guangdong 518132, China.

*Corresponding authors: Hao Huang, huang.hao@pku.edu.cn; Huan Zhou, zhouhuan@sari.ac.cn

Supplementary Table 1. **Analysis of protein sequences of TAB1 in cleaved sites across species**

Order	Organism	Cleavage site		Reference NCBI sequence
		Q10	Q444	
Primates	Human homo sapiens	SLLQSEQQ	LTLQSTNT	NP_006107.1
	Pan Troglodytes(chimpanzee)	SLLQSEQQ	LTLQSTNT	XP_016794684.1
Galliformes	Gallus	SSLQSEQQ	VTLQSTNT	NP_001006240.2
Rodentia	Mus Musculus	SLLQSEQQ	LTLQSTNT	NP_079885.2
	Rattus Norvegicus	SLLQSEQQ	LTLQSTNT	NP_001103446.1
Artiodactyla	Bos Taurus	SLLQSEQQ	LTLQSTNT	NP_001415199.1

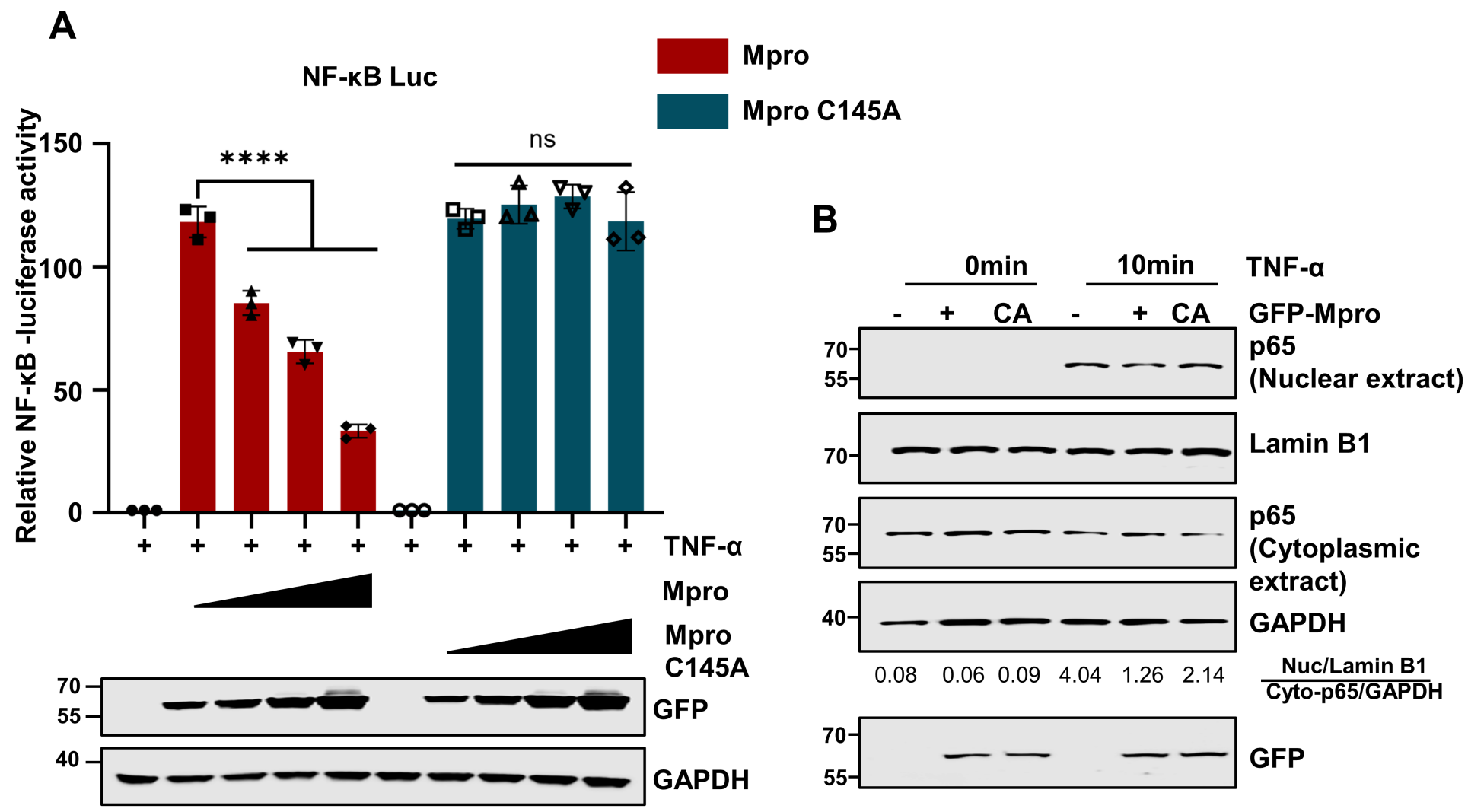

Supplementary Figure 1: SARS-CoV-2 Mpro suppresses immune signaling by cleaving TAB1. (A) Mpro dose-dependently inhibits TNF-α induced NF-κB activation. HEK-293T cells were co-transfected with NF-κB reporter plasmids, and increasing amounts of GFP-Mpro. NF-κB activity was assessed by dual-luciferase assay 24 h post-transfection and treatment TNF-α for 6h. (B) Mpro reduces the nuclear transfer of p65. HEK-293T transfected with GFP-Mpro, GFP-Mpro C145A, followed by western blot analysis nuclear of p65 and cytoplasmic of p65. *Data are presented as mean ± SD of three independent experiments, each performed in triplicate. Statistical significance was determined by one-way ANOVA and is indicated as follows: *P < 0.05, **P < 0.01, ***P < 0.001, ****P < 0.0001.

A

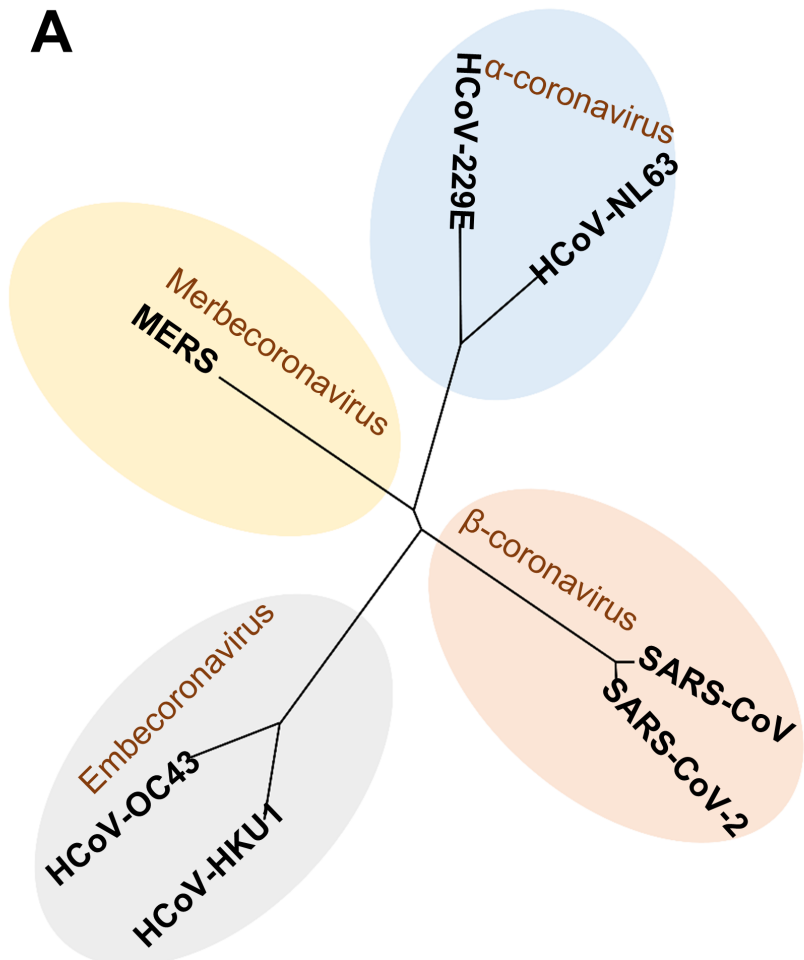

B

Identity (%)	SARS CoV2	SARS CoV	MERS	HCoV OC43	HCoV HKU1	HCoV NL63	HCoV 229E
SARS CoV2	100	96.1	47.7	46.5	47.5	40.9	38.7
SARS CoV	96.15	100	48.4	46.8	46.8	40.2	37.7
MERS	47.7	48.4	100	52.4	54.1	45.5	47.7
HCoV OC43	46.5	46.8	52.4	100	82.5	41.6	43.0
HCoV HKU1	47.5	46.8	54.1	82.5	100	41.5	44.4
HCoV NL63	40.9	40.2	45.5	41.6	41.5	100	69.8
HCoV 229E	38.7	37.7	47.7	43.0	44.4	69.8	100

C

Mpro	Residues	Molecular Weight (MW,kDa)	Isoelectric Point(pI)
SARS-CoV-2	306	33.8	5.95
SARS-CoV	306	33.8	6.22
MERS	306	33.8	5.86
HCoV-OC43	303	33.3	5.78
HCoV-HKU1	303	33.1	5.78
HCoV-NL63	303	32.7	6.39
HCoV-229E	302	33.0	6.38

Supplementary Figure 2: Overview of the seven human coronaviruses and their Mpros. (A) Phylogenetic tree analysis of seven human coronaviruses. SARS-CoV, MERS, SARS-CoV-2, HKU1 and OC43 are β-coronaviruses, while NL63 and 229E are α-coronaviruses (B) Structural comparison of coronavirus Mpro. The structures involved in the comparison were α-coronavirus Viruses, HCoV-NL63 (PDB ID: 5GWY), HCoV-229E (PDB ID: 2ZU2), β-coronavirus SARS-CoV (PDB ID: 5B6O), MERS-CoV (PDB ID: 5WKJ), and HCoV-HKU1 (PDB ID: 3D23) (PDB ID: 2Q6D). (C) Sequence identity of seven human coronaviral Mpros calculated by the maximum-likelihood method using MUSCLE.

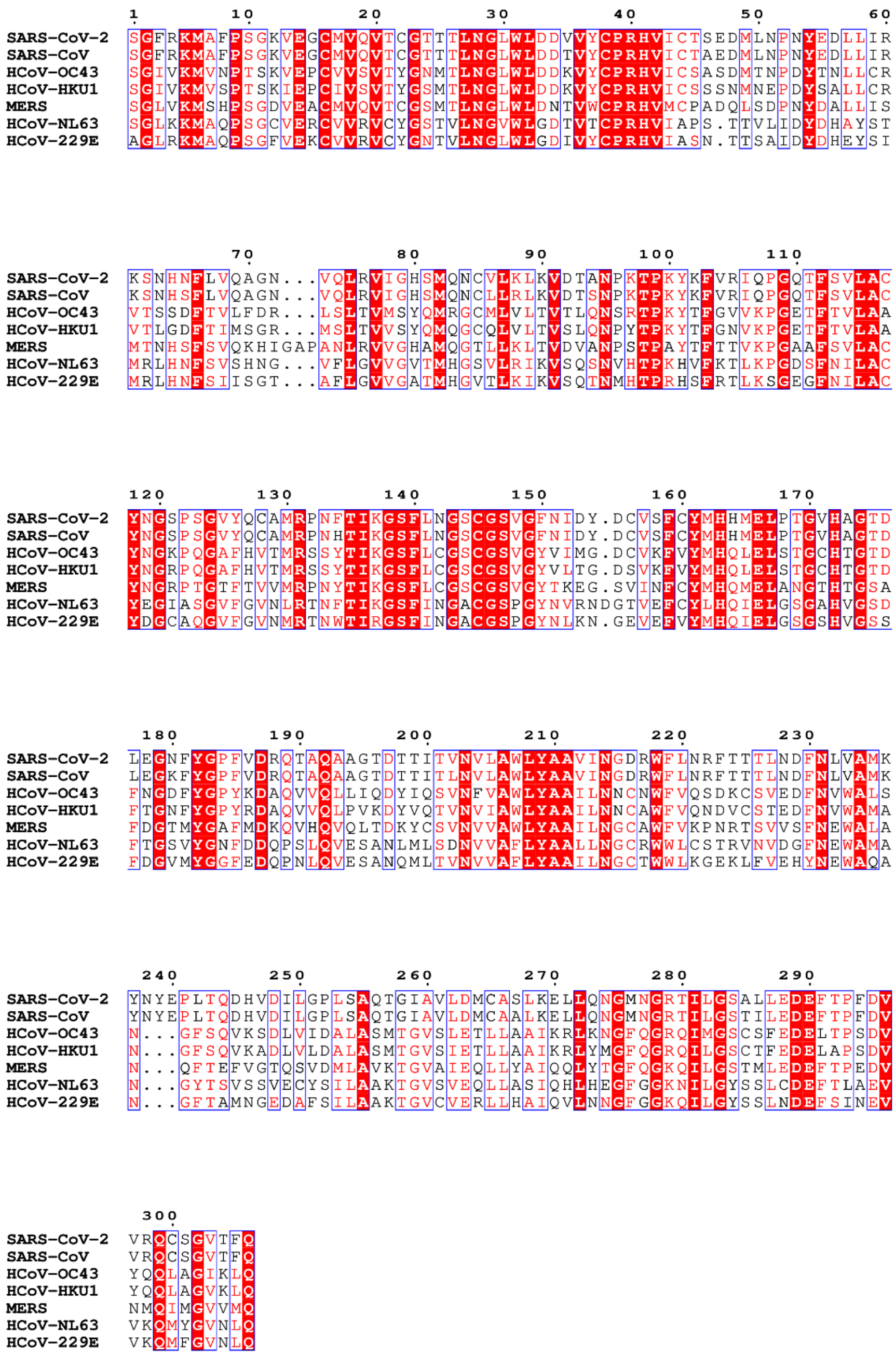

Supplementary Figure 3: Sequence alignment and conservation of Mpro sequences of seven human coronaviruses.

A sequence alignment diagram of the Mpro of seven human coronaviruses, comparing the full-length sequences of the main protease (Mpro) of SARS-CoV-2, SARS-CoV, MERS-CoV, HCoV-OC43, HCoV-HKU1, HCoV-NL63 and HCoV-229E. The comparison was made using the ESPript website (https://espript.ibcp.fr/ESPript/cgi-bin/ESPript.cgi).

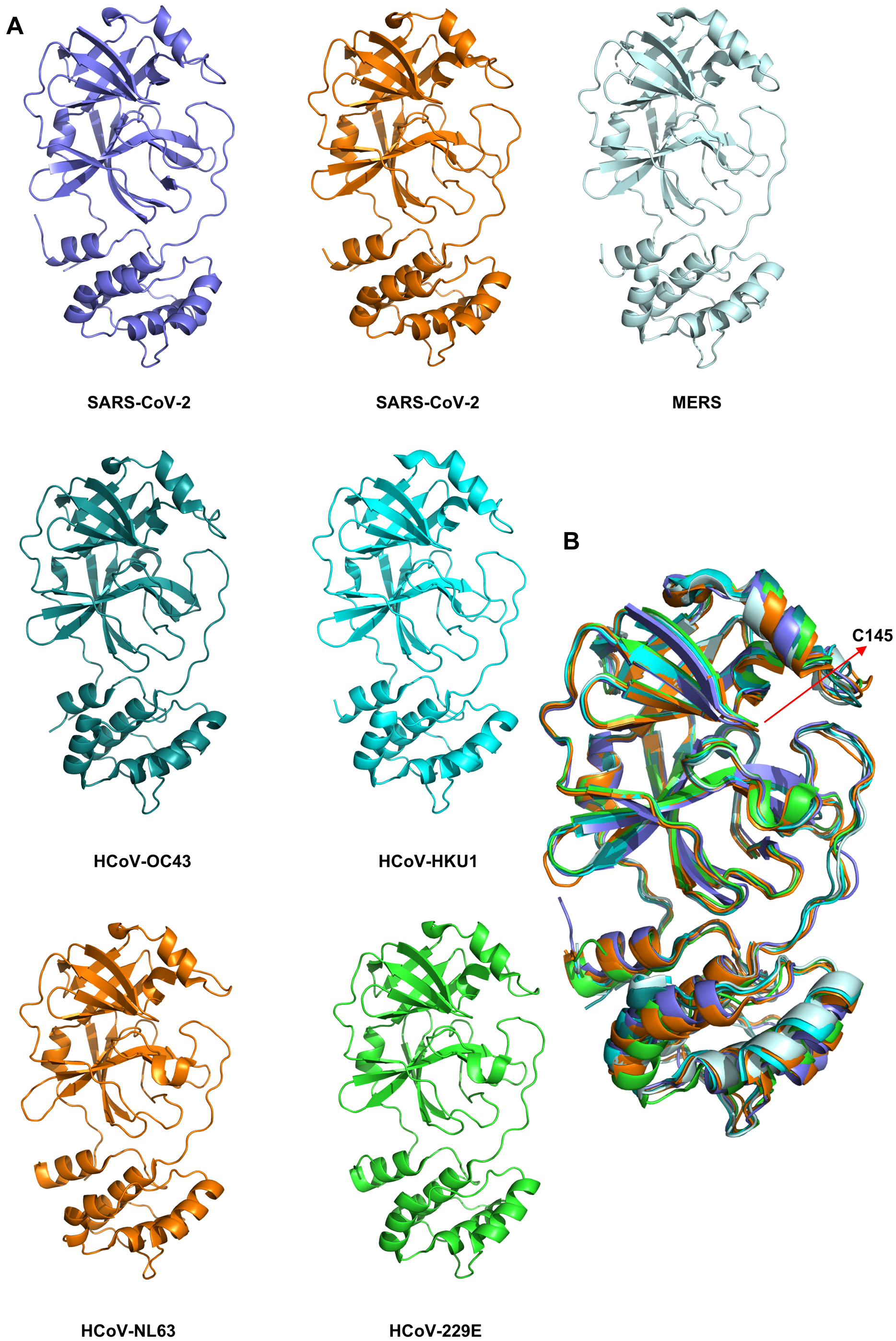

Supplementary Figure 4: Structural alignment of CoVs Mpro.

Red arrows indicate the conserved enzymatic proteolysis residue, Cys145. The three-dimensional structures were derived from the Protein Data Bank with the following accession numbers: SARS-CoV-2 (PDB: 9U96), SARS-CoV (PDB: 7ZQW), MERS (PDB: 5WKK), HCoV-OV43 (PDB: 9C7W), HCoV-HKU1(PDB: 3D23), HCoV-229E (PDB: 2ZU2), NL63 (PDB: 7E6M).

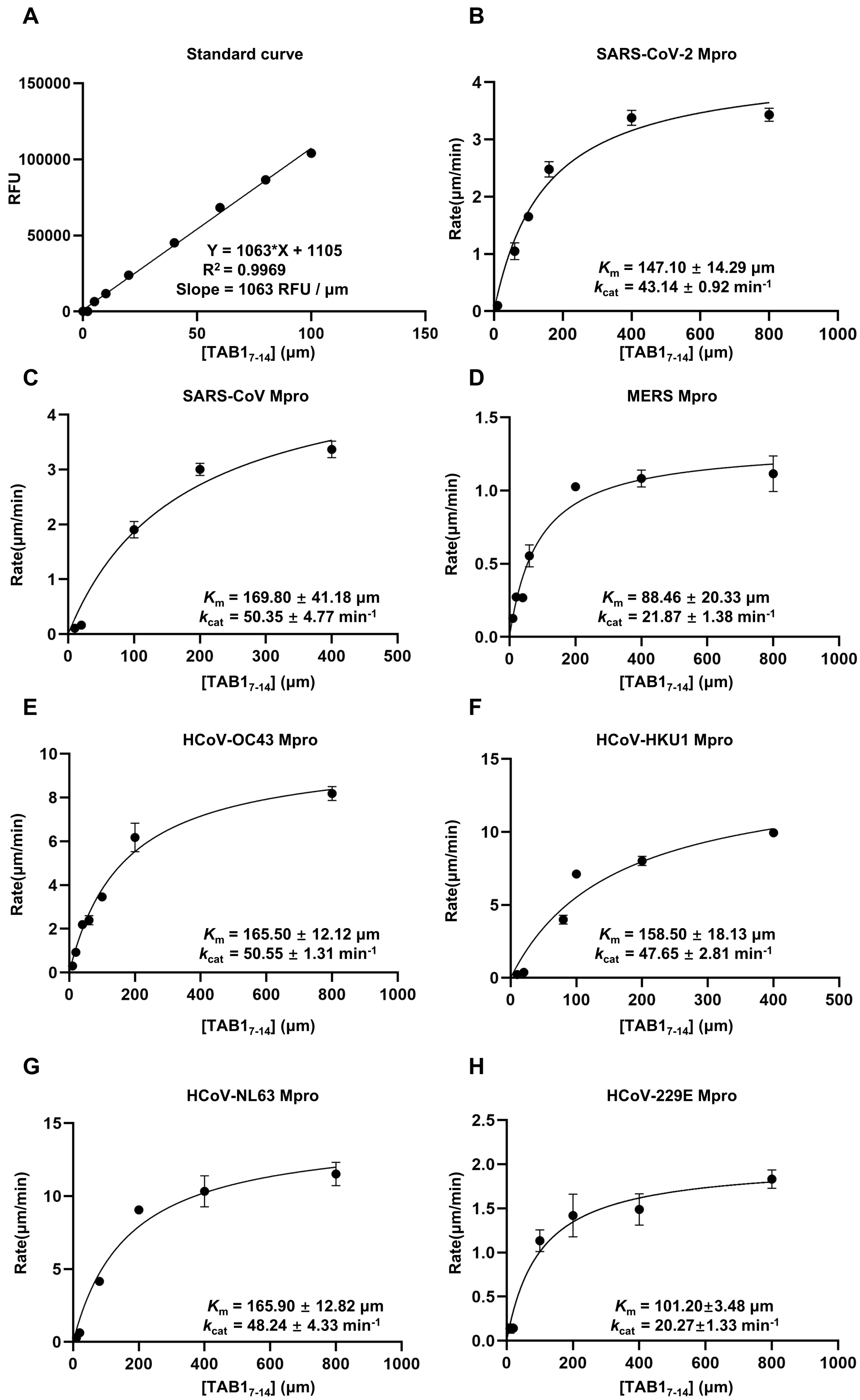
A
Standard curve
RFU
150000
100000
50000
0
0
50
100
150
Y = 1063*X + 1105
R² = 0.9969
Slope = 1063 RFU / μm
[TAB1₇₋₁₄] (μm)
B
SARS-CoV-2 Mpro
Rate(μm/min)
K_m = 147.10 ± 14.29 μm
k_{cat} = 43.14 ± 0.92 min⁻¹
[TAB1₇₋₁₄] (μm)
C
SARS-CoV Mpro
Rate(μm/min)
K_m = 169.80 ± 41.18 μm
k_{cat} = 50.35 ± 4.77 min⁻¹
[TAB1₇₋₁₄] (μm)
D
MERS Mpro
Rate(μm/min)
K_m = 88.46 ± 20.33 μm
k_{cat} = 21.87 ± 1.38 min⁻¹
[TAB1₇₋₁₄] (μm)
E
HCoV-OC43 Mpro
Rate(μm/min)
K_m = 165.50 ± 12.12 μm
k_{cat} = 50.55 ± 1.31 min⁻¹
[TAB1₇₋₁₄] (μm)
F
HCoV-HKU1 Mpro
Rate(μm/min)
K_m = 158.50 ± 18.13 μm
k_{cat} = 47.65 ± 2.81 min⁻¹
[TAB1₇₋₁₄] (μm)
G
HCoV-NL63 Mpro
Rate(μm/min)
K_m = 165.90 ± 12.82 μm
k_{cat} = 48.24 ± 4.33 min⁻¹
[TAB1₇₋₁₄] (μm)
H
HCoV-229E Mpro
Rate(μm/min)
K_m = 101.20±3.48 μm
k_{cat} = 20.27±1.33 min⁻¹
[TAB1₇₋₁₄] (μm)

Supplementary Figure 5. Kinetics of Mpro with MCA-TAB$1_{441-448}$-DNP

(A) Standard curves of MCA-TAB$1_{441-448}$-DNP. Three concentrations of 1, 5, and 10 μM MCA-TAB$1_{441-448}$-DNP substrates were completely digested with excess enzyme, and the maximum fluorescence was recorded thrice. The correlation between fluorescence units and substrate concentrations was fitted with simple linear regression functions in GraphPad Prism. Three independent experiments were performed for all enzymatic assays and are denoted as mean ± SEM. (A-H) based kinetics of Mpro of SARS-CoV 2, SARS-CoV, MERS, HCoV-OC43, HCoV-HKU1, HCoV-NL63, HCoV-229E. The K_m and k_{cat} values were obtained from the Michaelis–Menten equation in GraphPad Prism with the data of initial velocities V/[E] versus the concentrations of substrates [S]. All kinetic assays were performed in triplicates and plotted as means ± SEM (n = 3). Three independent experiments were performed for all enzymatic assays, and are denoted as mean ± SEM.

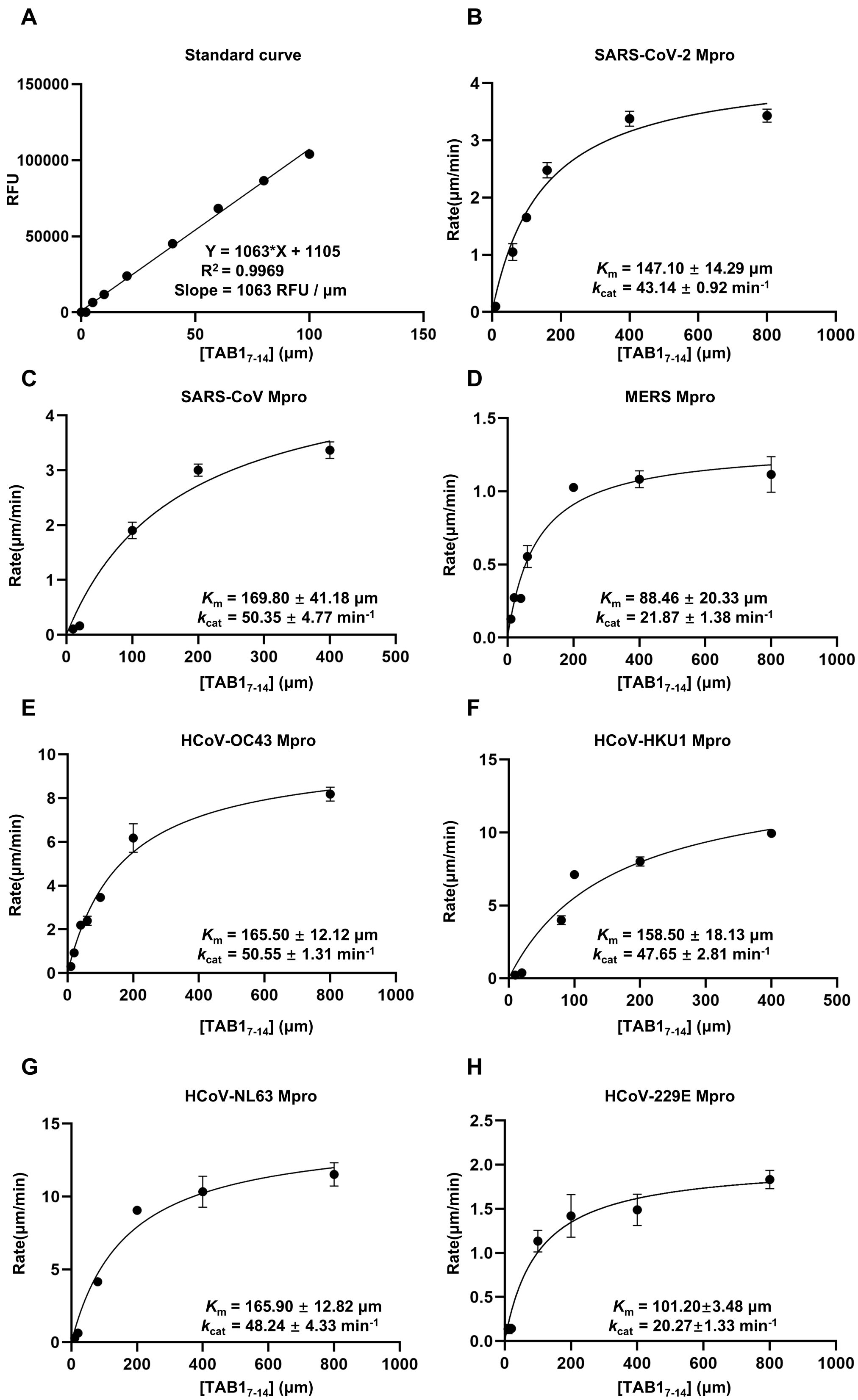
A
Standard curve
150000
100000
50000
0
RFU
0
50
100
150
[TAB1 7-14] (μm)
Y = 1063*X + 1105
R2 = 0.9969
Slope = 1063 RFU / μm
B
SARS-CoV-2 Mpro
Rate(μm/min)
Km = 147.10 ± 14.29 μm
kcat = 43.14 ± 0.92 min-1
[TAB1 7-14] (μm)
C
SARS-CoV Mpro
Rate(μm/min)
Km = 169.80 ± 41.18 μm
kcat = 50.35 ± 4.77 min-1
[TAB1 7-14] (μm)
D
MERS Mpro
Rate(μm/min)
Km = 88.46 ± 20.33 μm
kcat = 21.87 ± 1.38 min-1
[TAB1 7-14] (μm)
E
HCoV-OC43 Mpro
Rate(μm/min)
Km = 165.50 ± 12.12 μm
kcat = 50.55 ± 1.31 min-1
[TAB1 7-14] (μm)
F
HCoV-HKU1 Mpro
Rate(μm/min)
Km = 158.50 ± 18.13 μm
kcat = 47.65 ± 2.81 min-1
[TAB1 7-14] (μm)
G
HCoV-NL63 Mpro
Rate(μm/min)
Km = 165.90 ± 12.82 μm
kcat = 48.24 ± 4.33 min-1
[TAB1 7-14] (μm)
H
HCoV-229E Mpro
Rate(μm/min)
Km = 101.20±3.48 μm
kcat = 20.27±1.33 min-1
[TAB1 7-14] (μm)

Supplementary Figure 6. Kinetics of Mpro with MCA-TAB1$_{7\text{-}14}$-DNP

(A) Standard curves of MCA-TAB1$_{7\text{-}14}$-DNP. Three concentrations of 1, 5, and 10 μM MCA-TAB1$_{7\text{-}14}$-DNP substrates were completely digested with excess enzyme, and the maximum fluorescence was recorded thrice. The correlation between fluorescence units and substrate concentrations was fitted with simple linear regression functions in GraphPad Prism. Three independent experiments were performed for all enzymatic assays and are denoted as mean ± SEM. (A-H) based kinetics of Mpro of SARS-CoV 2, SARS-CoV, MERS, HCoV-OC43, HCoV-HKU1, HCoV-NL63, HCoV-229E. The K_m and k_{cat} values were obtained from the Michaelis–Menten equation in GraphPad Prism with the data of initial velocities V/[E] versus the concentrations of substrates [S]. All kinetic assays were performed in triplicates and plotted as means ± SEM (n = 3). Three independent experiments were performed for all enzymatic assays, and are denoted as mean ± SEM.

Supplementary Table 2. Predicted interactions of seven Mpros bound TAB1441-445 analyzed with PDBePISA (https://www.ebi.ac.uk/pdbe/pisa/)

Residue hTAB1	SARS-CoV-2	SARS-CoV	MERS-CoV	HCoV-OC43	HCoV-HKU1	HCoV-NL63	HCoV-229E
L1	GLU166(N-O) HB (2.62 Å)	GLU166(N-O) HB (2.84 Å)	GLU169(N-O) HB (3.21 Å)	GLU(N-O) HB (3.07 Å)	Glu166(N-O) HB (2.98 Å)	GLU166(N-O) HB (3.43 Å)	GLU165(N-O) HB (2.80 Å)
			Val192(O-N) HB (3.33 Å)			SER190(O-N) HB (2.87Å)	
T2	GLU166(N-O) HB (2.6 Å)	GLU166(N-O) HB (3.92 Å)	GLU169(O-N) HB (3.27 Å)	GLU166(N-O) HB (3.32 Å)	GLU166(N-O) HB (3.56 Å)	GLU166(O-O) HB (3.59 Å)	GLU165(N-O) HB (2.99 Å)
		GLU166(O-N) HB (3.11 Å)	GLU169(N-O) HB (3.52 Å)	GLU166(O-N) HB (3.18 Å)	GLU166(O-N) HB (3.27 Å)	GLU166(O-N) HB (3.45 Å)	GLU166(O-N) HB (2.95 Å)
L3			GLU192(N-OE1) HB (3.07 Å)		GLU189(O-NE2) HB (3.51 Å)		
Q4	HIS164(N-O) HB (3.24 Å)	HIS164(N-O) HB (3.21 Å)	GLN167(N-O) HB (3.33 Å)	GLN164(N-O) HB (3.41 Å)	HIS164(N-O) HB (3.30 Å)	HIS164(N-O) HB (3.56 Å)	GLN163(N-O) HB (3.62 Å)
	GLU166(NE2-OE1) HB (3.72 Å)	SER144(NE2-OG) HB (3.64 Å)	PHE143(NE2-O) HB (3.71 Å)	GLU166(NE2-OE2) HB (3.73 Å)	PHE140(NE2-O) HB (3.78 Å)		
	SER144(NE2-OG) HB (3.55 Å);	PHE140(NE2-O) HB (3.21 Å)	CYS148(O-N) HB (3.46 Å)	PHE140(NE2-O) HB (3.50 Å)	SER144(NE2-OG) HB (3.65 Å)		
	GLY143(O-N) HB (3.82 Å)	HIS41(O-NE2) HB (3.88 Å)			GLY145(O-N) HB (3.32 Å)		
		ASN142(OE1-ND2) HB (3.74 Å)					
S5	HIS41(O-ND1) Salt bridges (3.98 Å)	HIS41(O-NE2) Salt bridges (3.98 Å)	HIS41(O-NE2) Salt bridges (2.40 Å)	CYS142(N-O) HB (3.20 Å)	HIS41(O-NE2) Salt bridges (3.87 Å)	ASN141(N-OD1) HB (3.74 Å)	CYS144(N-SG) HB (3.72 Å)
				CYS142(N-SG) HB (2.59 Å)	HIS41(O-ND1) Salt bridges (2.74 Å)	ASN141(OG-ND2) HB (3.46 Å)	HIS41(O-NE2) Salt bridges (2.51 Å)
				HIS41(O-NE2) Salt bridges (3.95 Å)		HIS41(O-ND1) Salt bridges (3.31 Å)	
				HIS41(O-ON1) Salt bridges (2.42 Å)			

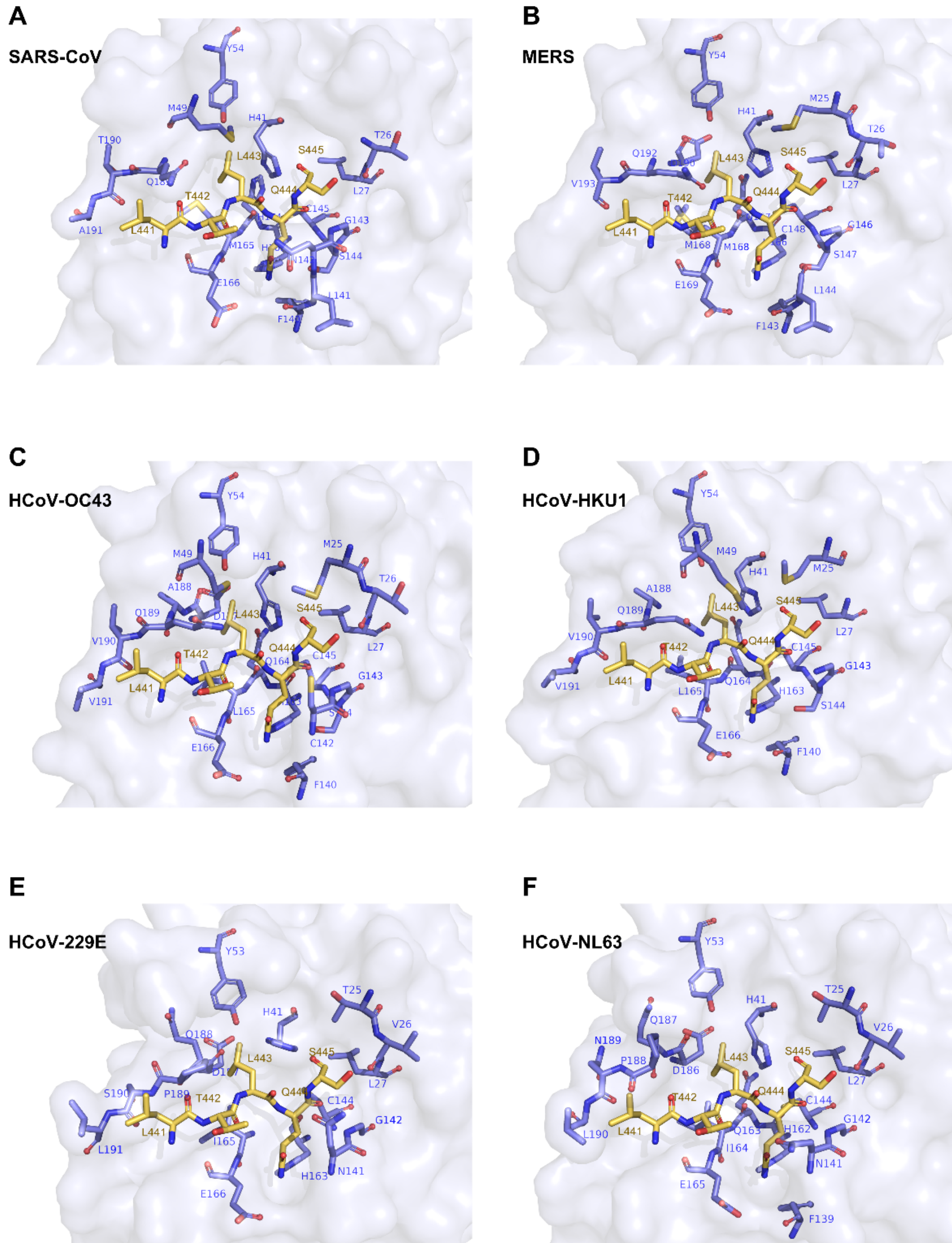

Supplementary Figure 7. Structure models of the TAB1-bound six Mpros

(A)-(F) Structural superposition of Mpro domains from six human coronaviruses aligned onto the SARS-CoV-2 Mpro template (PDB: 9U96). Structures include (A)SARS-CoV (PDB: 7ZQW), (B) MERS-CoV (PDB: 5WKK), (C) HCoV-OV43 (PDB: 9C7W), (D) HCoV-HKU1(PDB: 3D23) (E) HCoV-229E (PDB: 2ZU2), (F) NL63 (PDB: 7E6M)

Fig. 1A

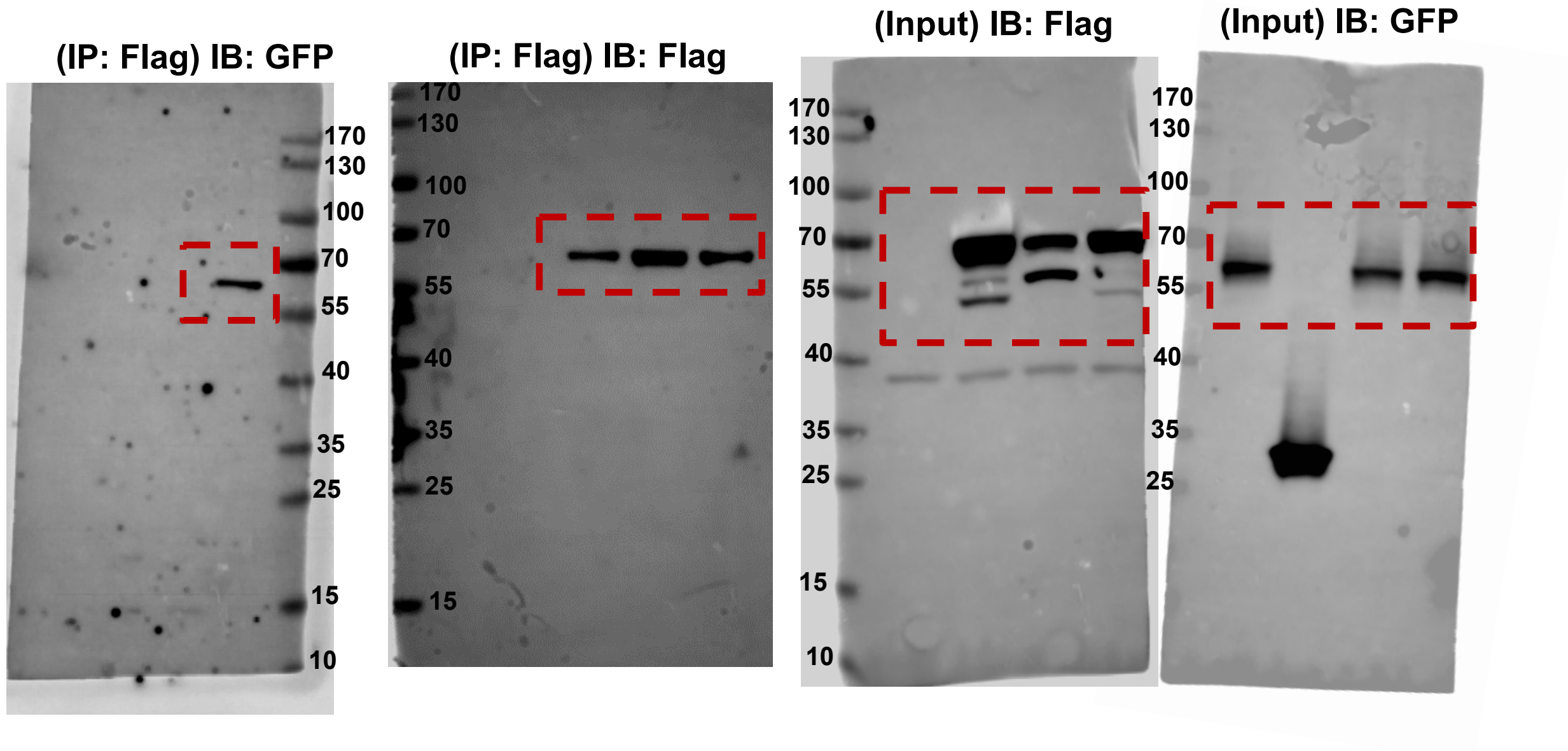

Fig. 1B

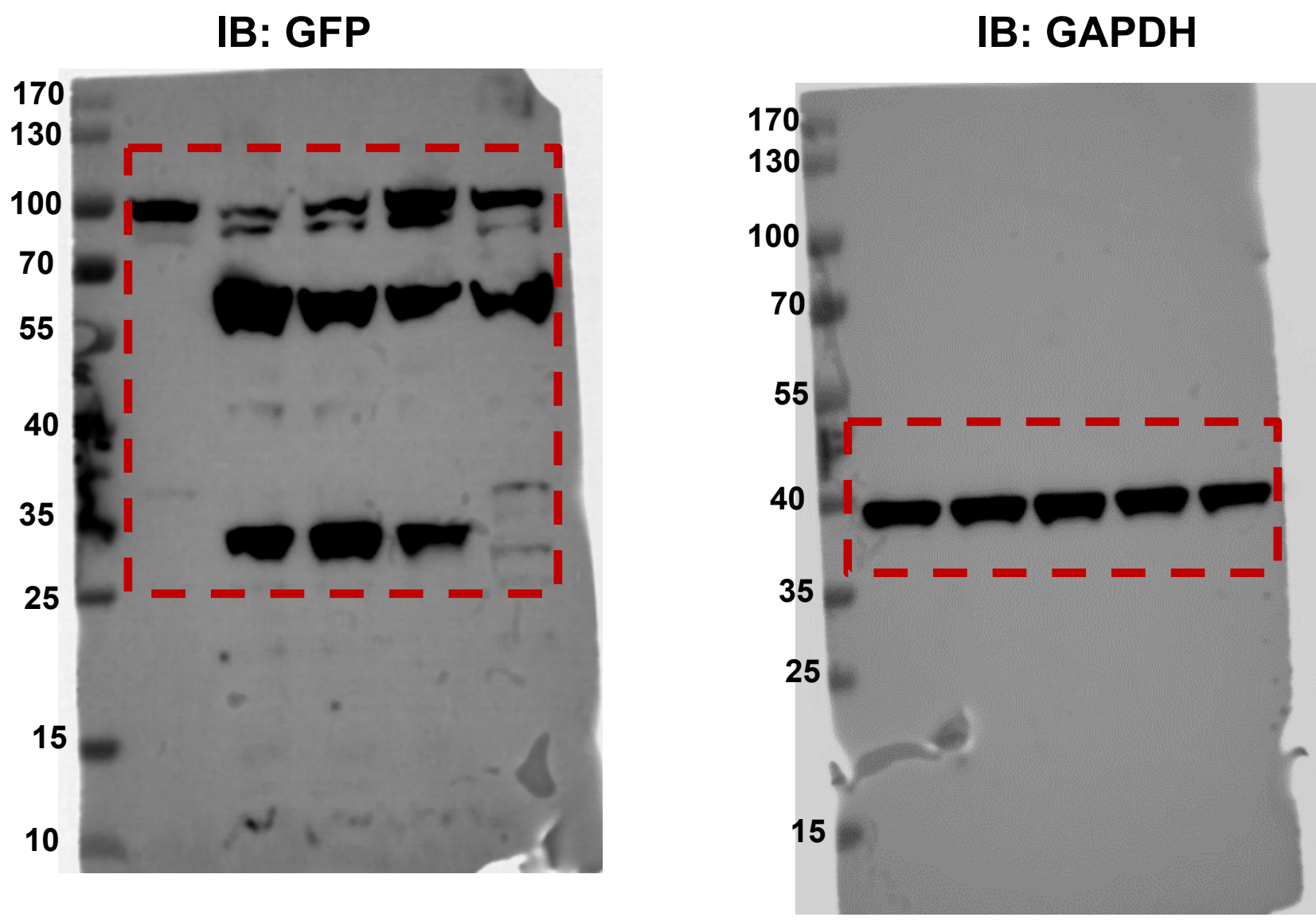

Fig. 1C

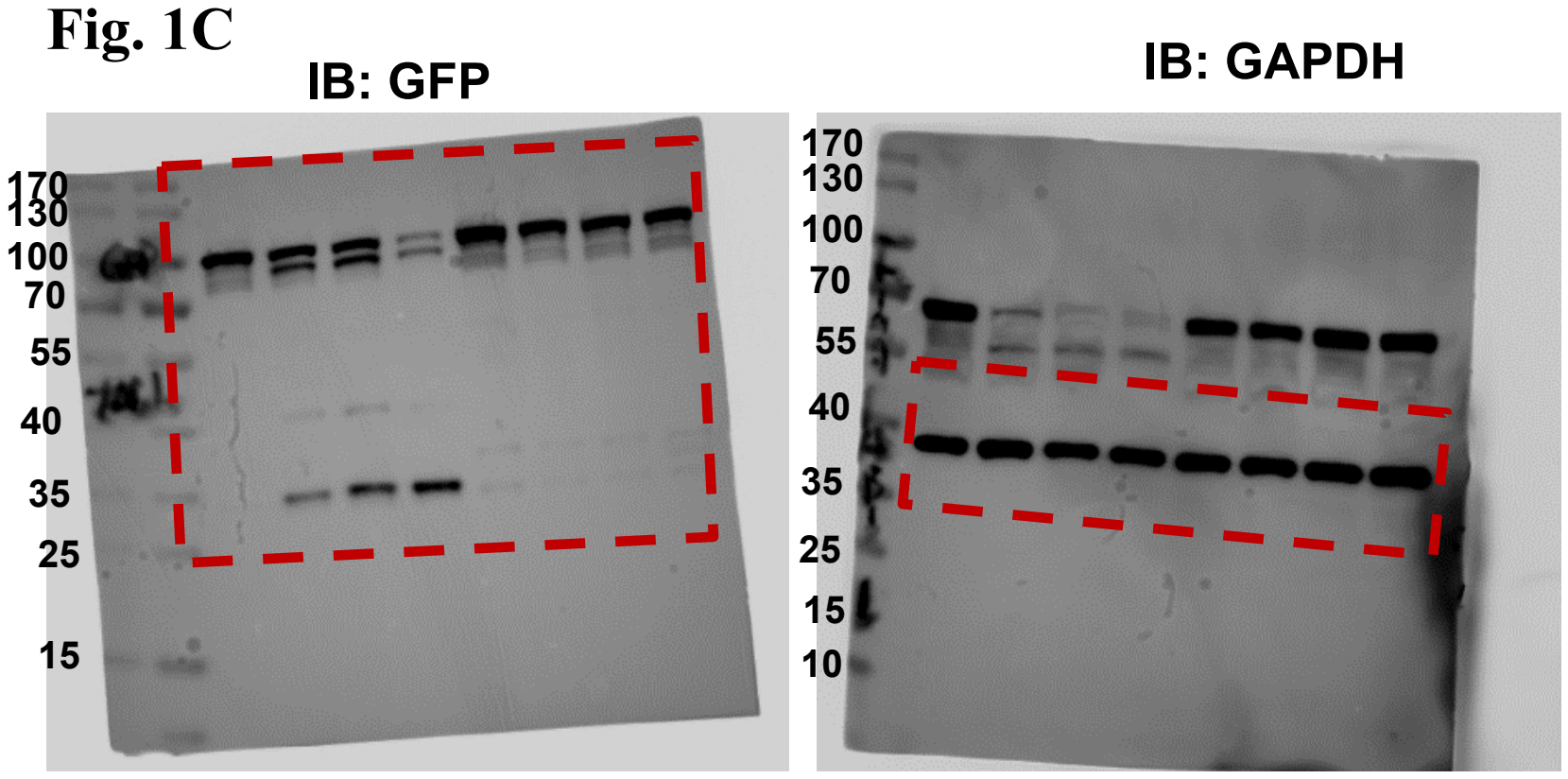

Fig. 1D

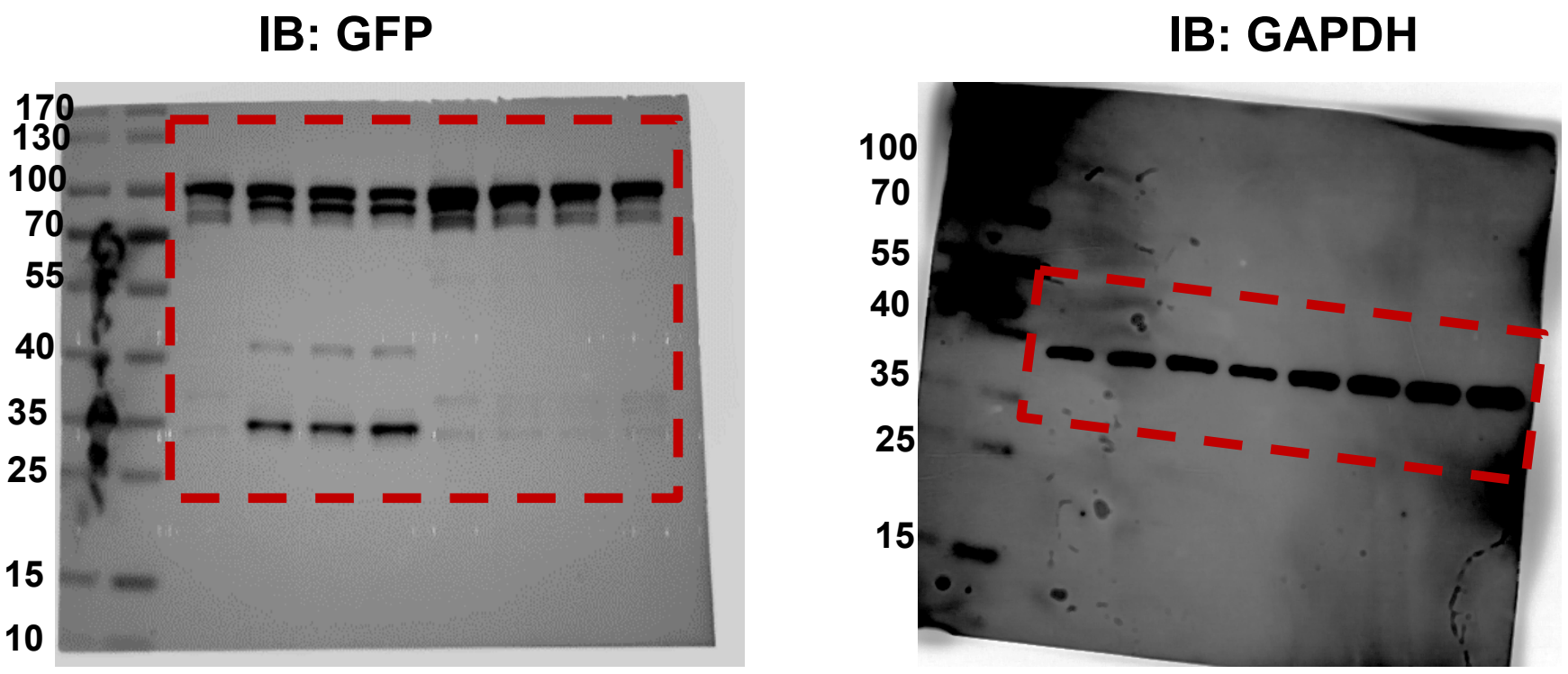

Fig. 2C

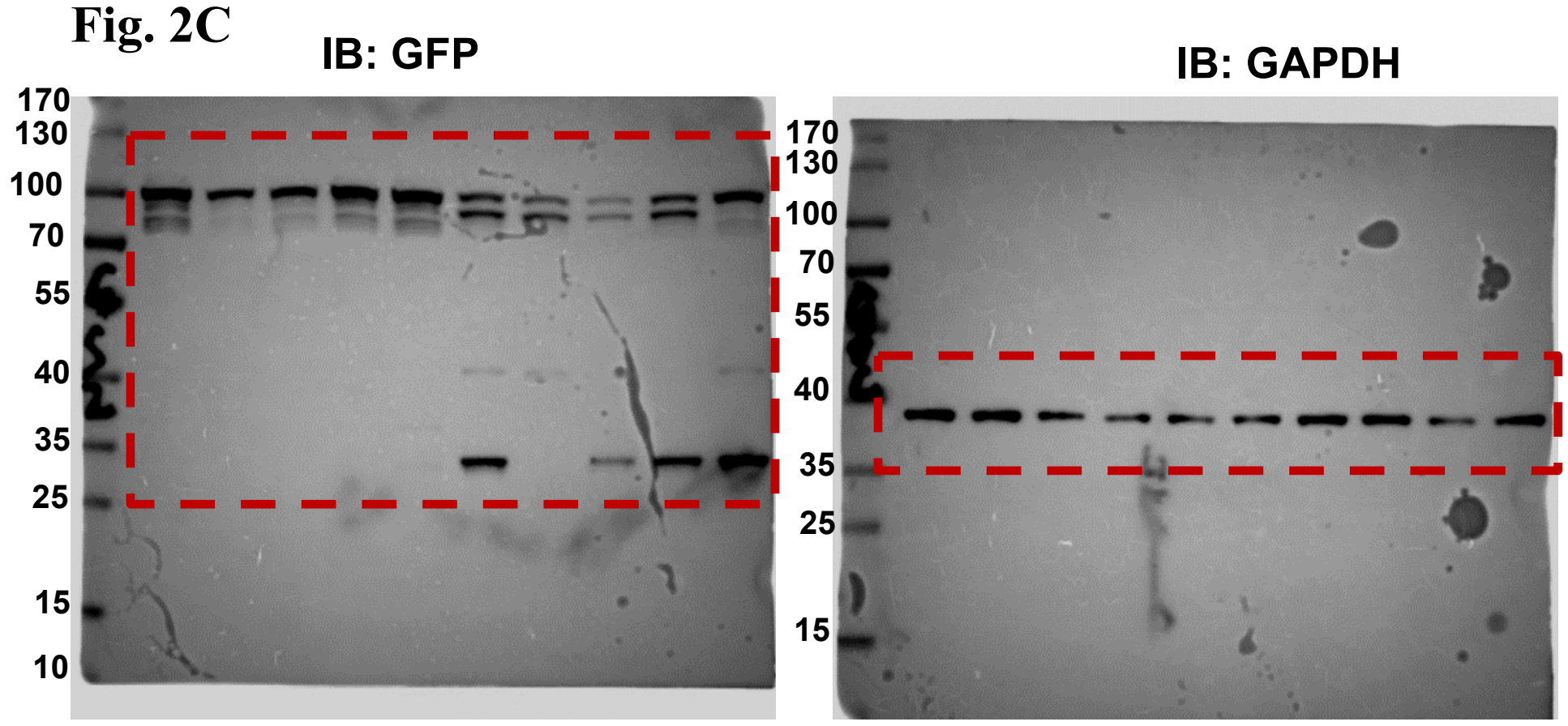

Fig. 2D

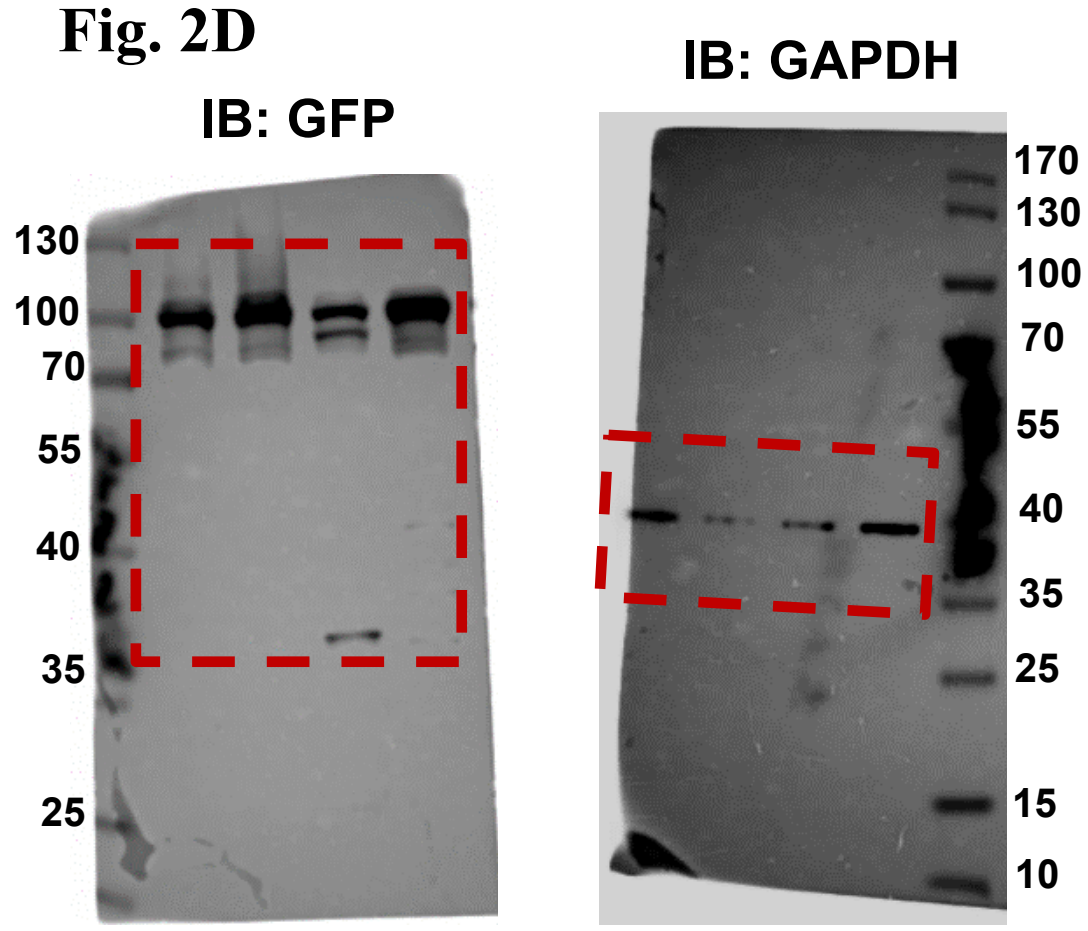

Fig. 2E

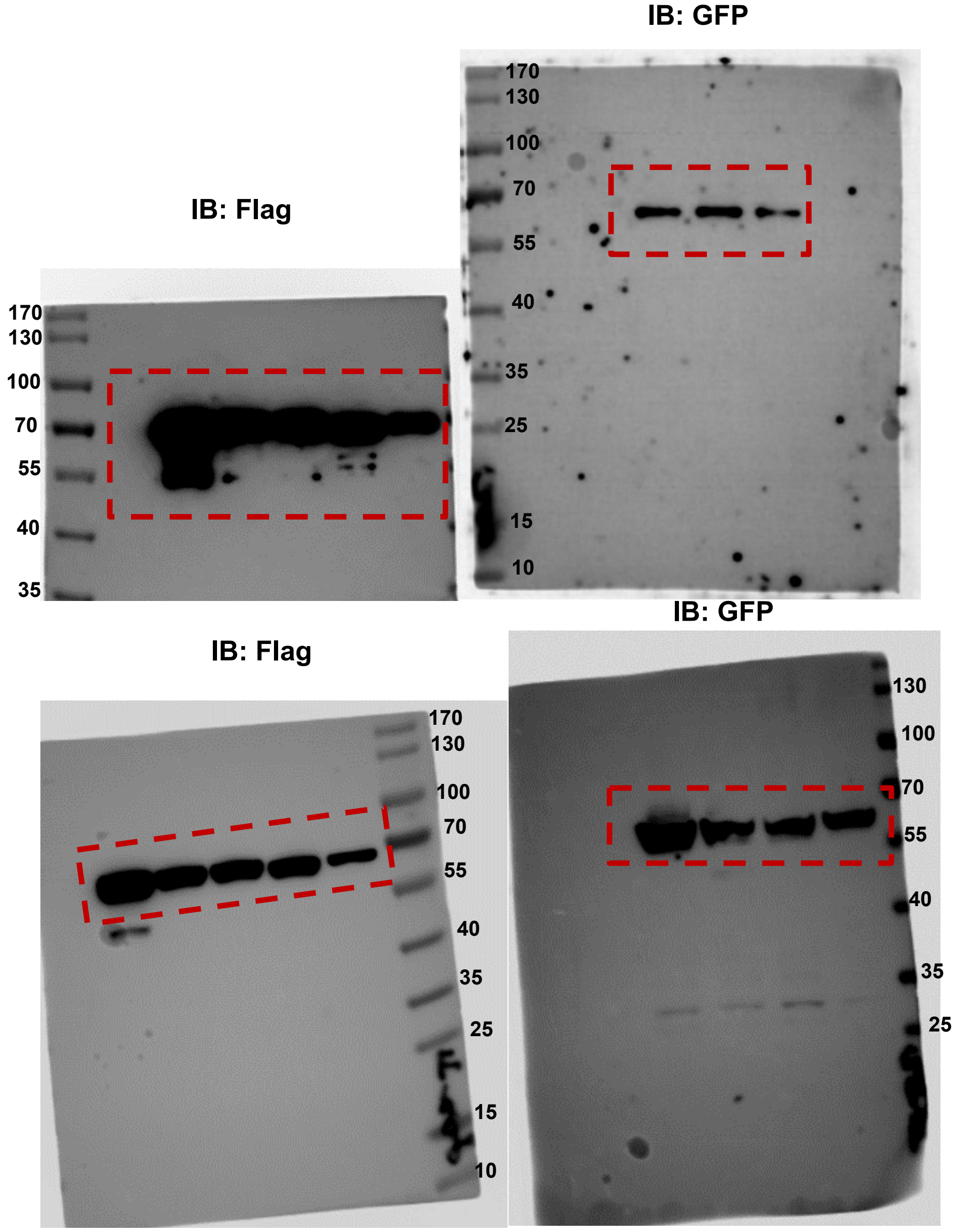
IB: GFP
170
130
100
70
55
40
35
25
15
10
IB: Flag
170
130
100
70
55
40
35
IB: Flag
170
130
100
70
55
40
35
25
15
10
IB: GFP
130
100
70
55
40
35
25

Fig. 3E

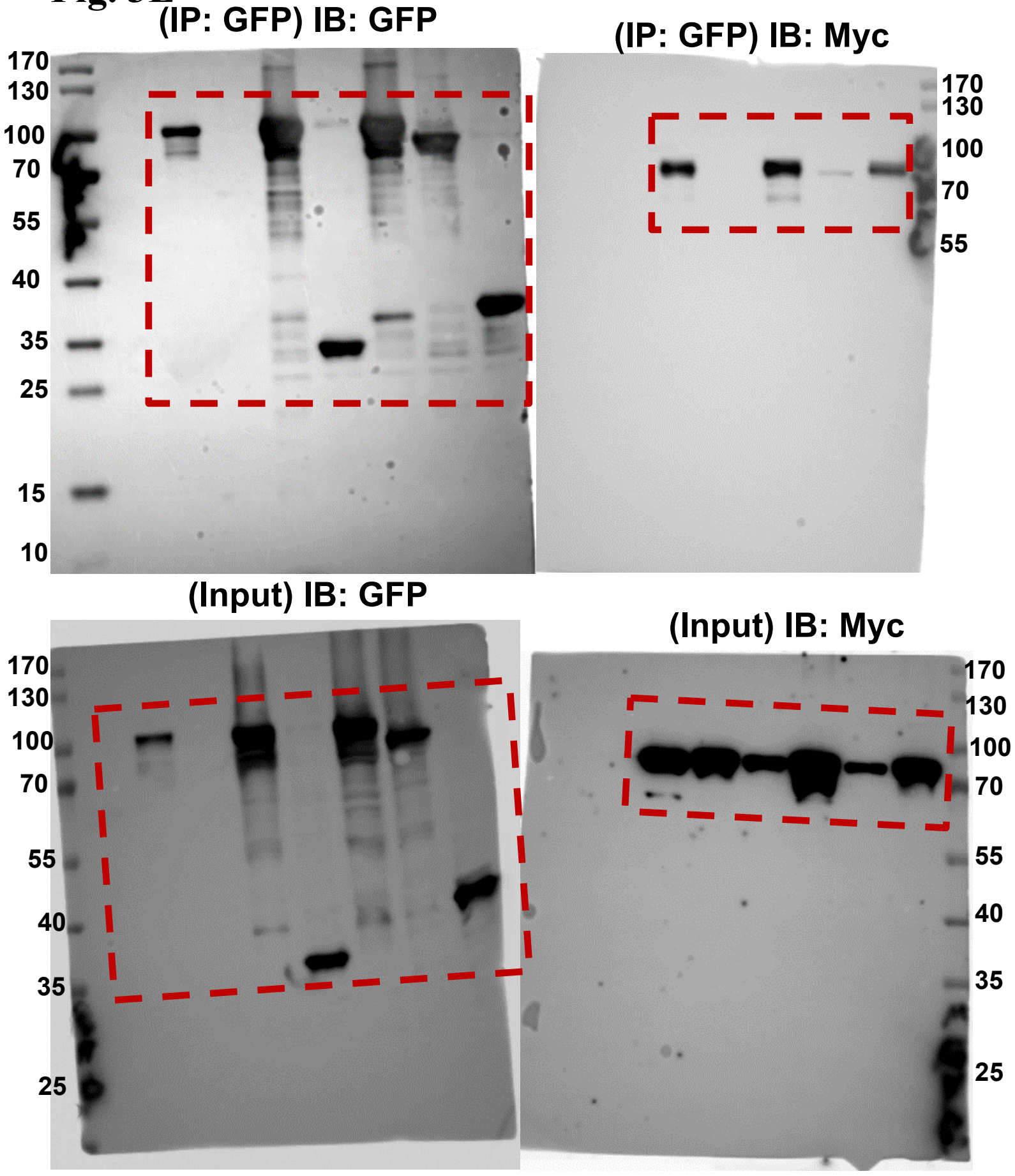
(IP: GFP) IB: GFP
(IP: GFP) IB: Myc
(Input) IB: GFP
(Input) IB: Myc

Fig. 3F

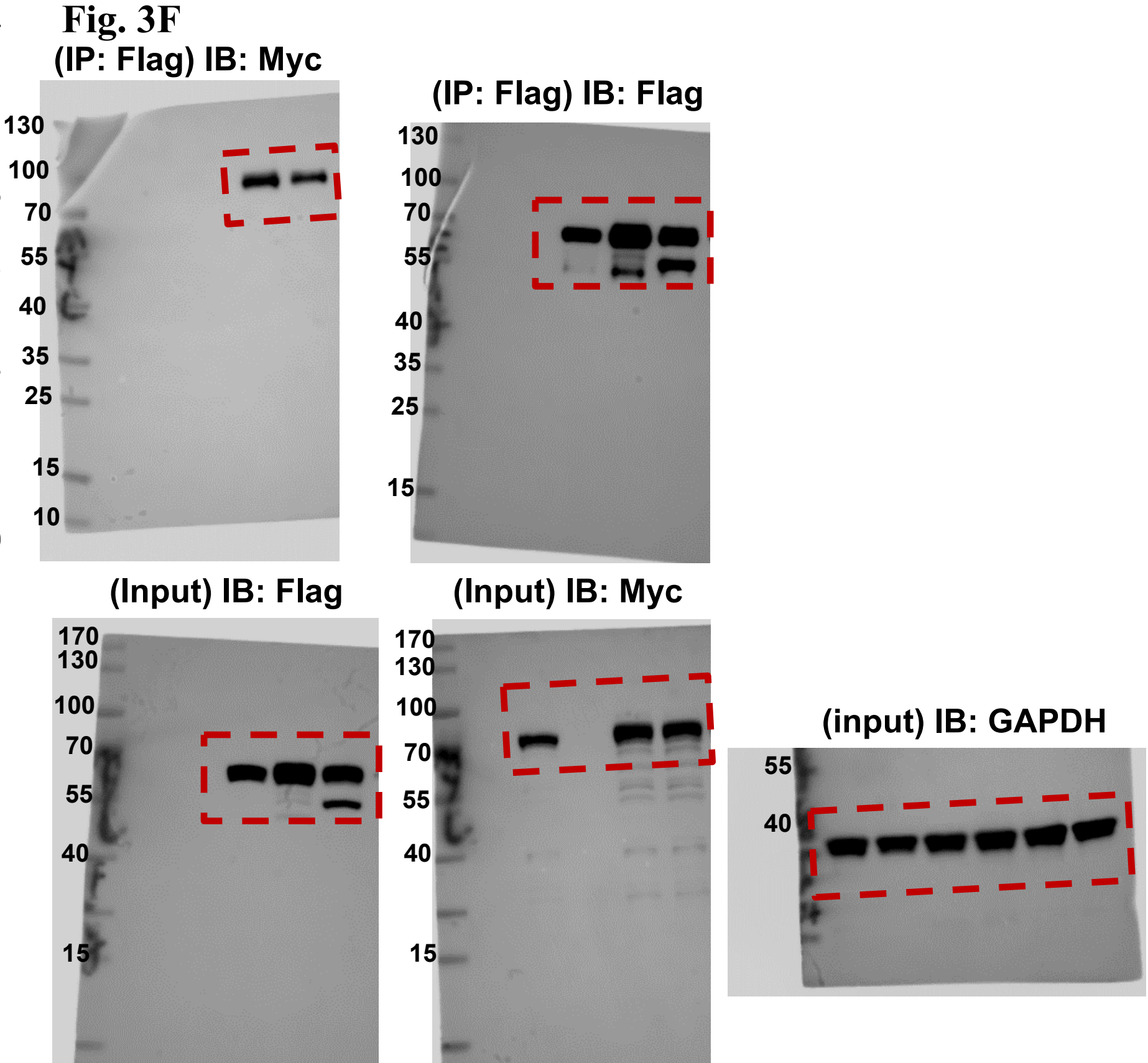
(IP: Flag) IB: Myc
(IP: Flag) IB: Flag
(Input) IB: Flag
(Input) IB: Myc
(input) IB: GAPDH

Fig. 4B

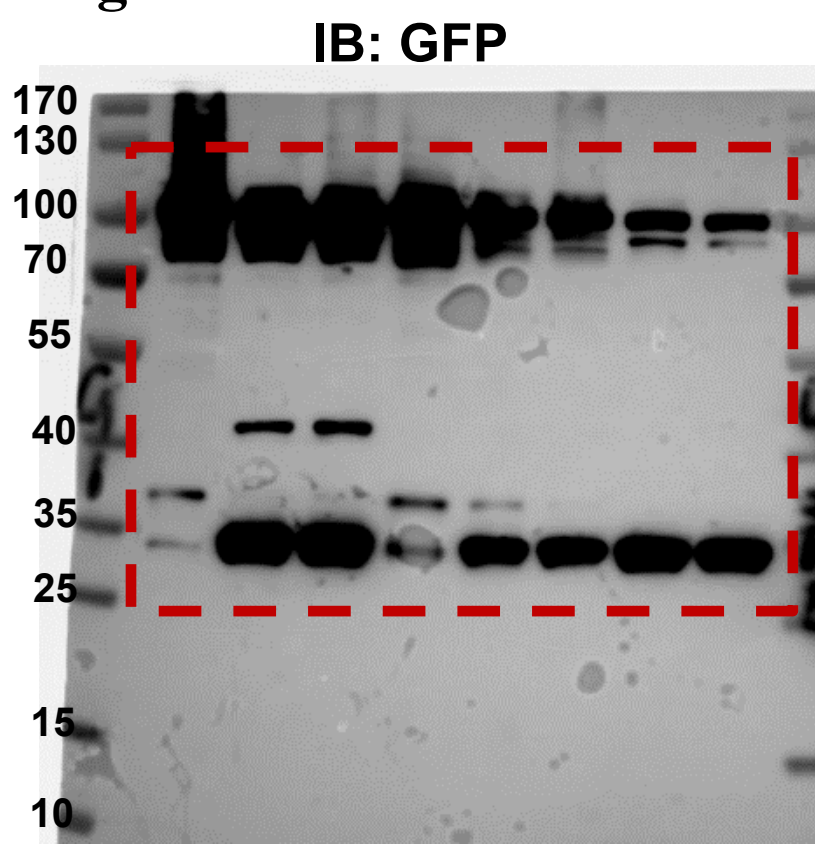

Fig. 4C

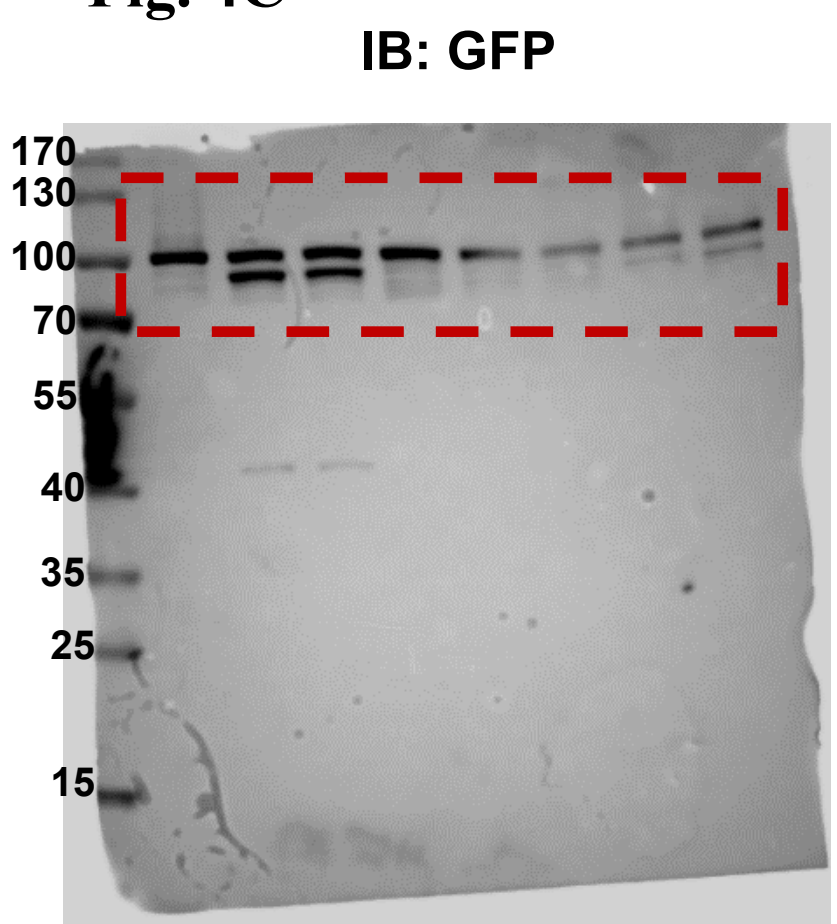

Fig. 4D

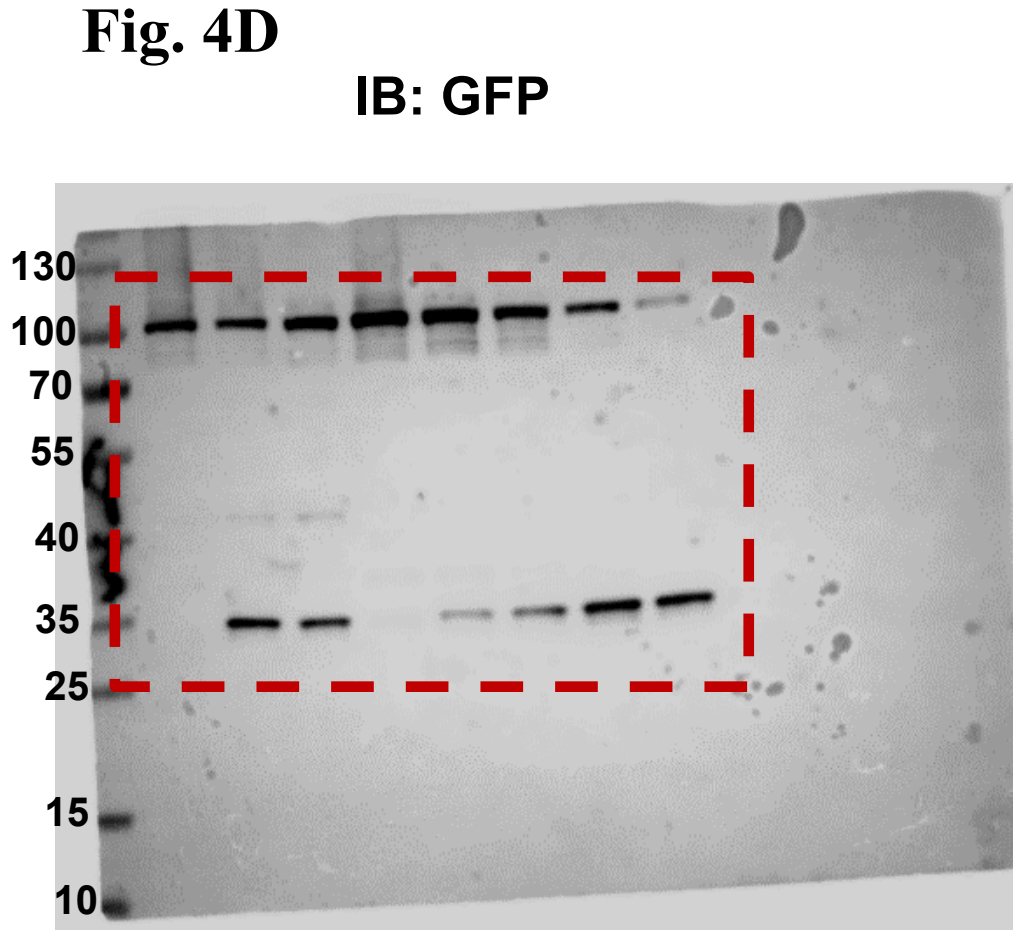

Fig.4E

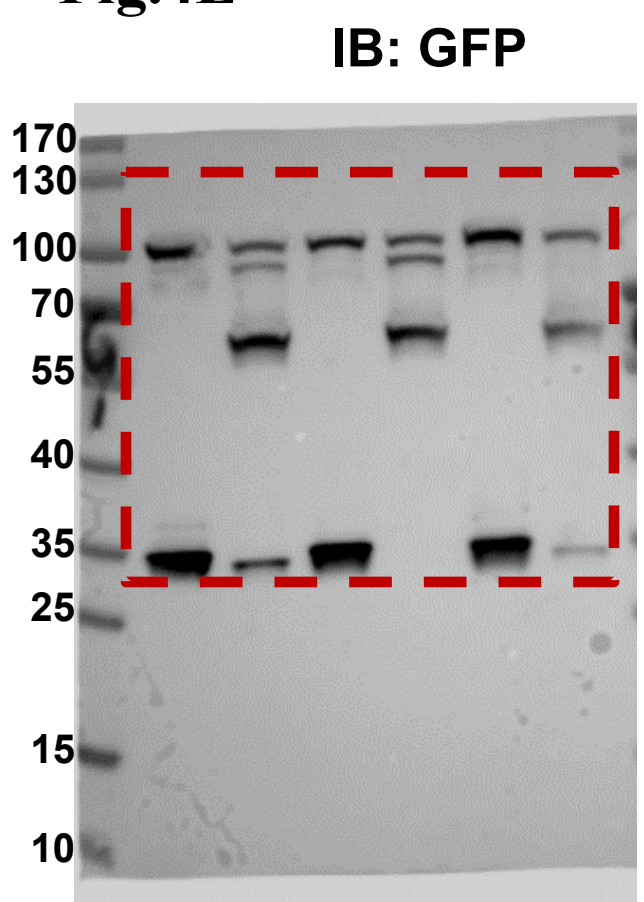
IB: GFP
170
130
100
70
55
40
35
25
15
10

Fig. 4F

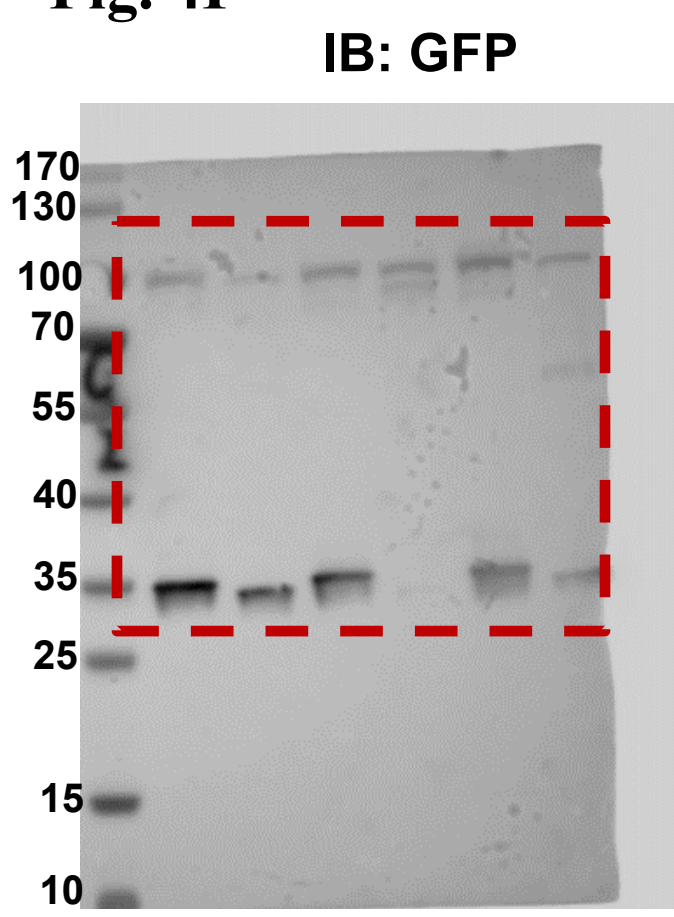
IB: GFP
170
130
100
70
55
40
35
25
15
10

Fig.S1B

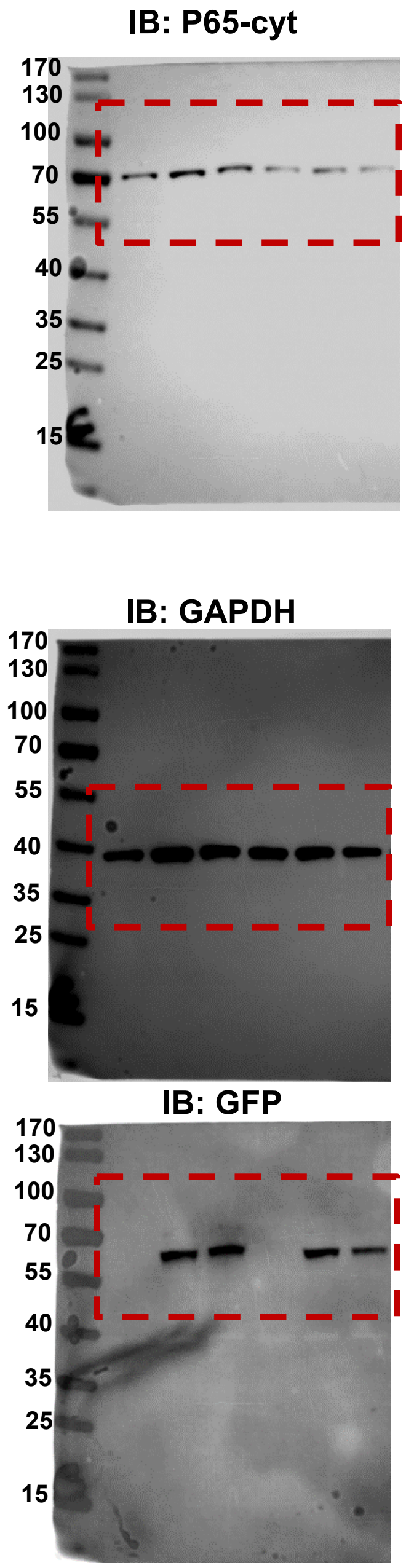
IB: P65-cyt
170
130
100
70
55
40
35
25
15
IB: GAPDH
170
130
100
70
55
40
35
25
15
IB: GFP
170
130
100
70
55
40
35
25
15

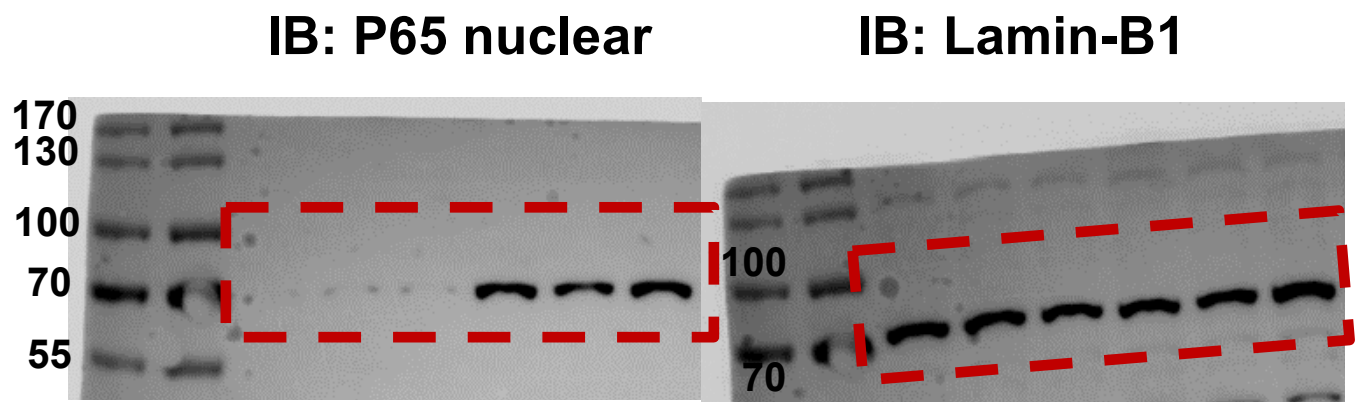

Supplementary Figure 8.

Unedited and uncropped blots from all figures in the paper. Dashed-line box inserts indicate cropped region used in the main figure.
