# Supplementary material for: The seven human coronavirus main proteases exhibit differential modulation of NF-κB suppression via TAB1 cleavage
Source: Commun Biol. 2026 May 21;9:1087. doi: 10.1038/s42003-026-10225-9 (PMC13462985; doi:10.1038/s42003-026-10225-9)
Supplement: Supplementary file 2 [file 42003_2026_10225_MOESM2_ESM.pdf]

Description of Additional Supplementary Files:

File name: Supplementary Data 1

Description: Source data can be found in Supplementary Data 1.
