# Supplementary material for: The seven human coronavirus main proteases exhibit differential modulation of NF-κB suppression via TAB1 cleavage
Source: Commun Biol. 2026 May 21;9:1087. doi: 10.1038/s42003-026-10225-9 (PMC13462985; doi:10.1038/s42003-026-10225-9)
Supplement: Supplementary file 3 [file 42003_2026_10225_MOESM4_ESM.pdf]

nature portfolio

Corresponding author(s): COMMSBIO-25-10053C

Last updated by author(s): Apr 21, 2026

Reporting Summary

Nature Portfolio wishes to improve the reproducibility of the work that we publish. This form provides structure for consistency and transparency in reporting. For further information on Nature Portfolio policies, see our Editorial Policies and the Editorial Policy Checklist.

Statistics

For all statistical analyses, confirm that the following items are present in the figure legend, table legend, main text, or Methods section.

n/a	Confirmed	
☐	☒	The exact sample size (n) for each experimental group/condition, given as a discrete number and unit of measurement
☐	☒	A statement on whether measurements were taken from distinct samples or whether the same sample was measured repeatedly
☒	☐	The statistical test(s) used AND whether they are one- or two-sided<br>*Only common tests should be described solely by name; describe more complex techniques in the Methods section.*
☒	☐	A description of all covariates tested
☒	☐	A description of any assumptions or corrections, such as tests of normality and adjustment for multiple comparisons
☐	☒	A full description of the statistical parameters including central tendency (e.g. means) or other basic estimates (e.g. regression coefficient) AND variation (e.g. standard deviation) or associated estimates of uncertainty (e.g. confidence intervals)
☒	☐	For null hypothesis testing, the test statistic (e.g. F, t, r) with confidence intervals, effect sizes, degrees of freedom and P value noted<br>*Give P values as exact values whenever suitable.*
☒	☐	For Bayesian analysis, information on the choice of priors and Markov chain Monte Carlo settings
☒	☐	For hierarchical and complex designs, identification of the appropriate level for tests and full reporting of outcomes
☒	☐	Estimates of effect sizes (e.g. Cohen's d, Pearson's r), indicating how they were calculated

Our web collection on statistics for biologists contains articles on many of the points above.

Software and code

Policy information about availability of computer code

Data collection	*Provide a description of all commercial, open source and custom code used to collect the data in this study, specifying the version used OR state that no software was used.*
Data analysis	CCP4 (v9.0.003); Coot (v0.9.8.95);Phenix(1.21.2_5419);Pymol(2.3.S);Image lab software (6.0.1);GraphPad Prism (v9.5.1);

For manuscripts utilizing custom algorithms or software that are central to the research but not yet described in published literature, software must be made available to editors and reviewers. We strongly encourage code deposition in a community repository (e.g. GitHub). See the Nature Portfolio guidelines for submitting code & software for further information.

Data

Policy information about availability of data

All manuscripts must include a data availability statement. This statement should provide the following information, where applicable:

- Accession codes, unique identifiers, or web links for publicly available datasets
- A description of any restrictions on data availability
- For clinical datasets or third party data, please ensure that the statement adheres to our policy

All relevant structure data supporting the findings of this study are available in the PDB DOI: https://doi.org/10.2210/pdb9U96/pdb
The datasets generated during this study are fully available within the article and supplementary materials.
The data that support the findings of this study are available from the corresponding author upon reasonable request.

Research involving human participants, their data, or biological material

Policy information about studies with human participants or human data. See also policy information about sex, gender (identity/presentation), and sexual orientation and race, ethnicity and racism.

Reporting on sex and gender	*Use the terms sex (biological attribute) and gender (shaped by social and cultural circumstances) carefully in order to avoid confusing both terms. Indicate if findings apply to only one sex or gender; describe whether sex and gender were considered in study design; whether sex and/or gender was determined based on self-reporting or assigned and methods used.*<br>*Provide in the source data disaggregated sex and gender data, where this information has been collected, and if consent has been obtained for sharing of individual-level data; provide overall numbers in this Reporting Summary. Please state if this information has not been collected.*<br>*Report sex- and gender-based analyses where performed, justify reasons for lack of sex- and gender-based analysis.*
Reporting on race, ethnicity, or other socially relevant groupings	*Please specify the socially constructed or socially relevant categorization variable(s) used in your manuscript and explain why they were used. Please note that such variables should not be used as proxies for other socially constructed/relevant variables (for example, race or ethnicity should not be used as a proxy for socioeconomic status).*<br>*Provide clear definitions of the relevant terms used, how they were provided (by the participants/respondents, the researchers, or third parties), and the method(s) used to classify people into the different categories (e.g. self-report, census or administrative data, social media data, etc.)*<br>*Please provide details about how you controlled for confounding variables in your analyses.*
Population characteristics	*Describe the covariate-relevant population characteristics of the human research participants (e.g. age, genotypic information, past and current diagnosis and treatment categories). If you filled out the behavioural & social sciences study design questions and have nothing to add here, write "See above."*
Recruitment	*Describe how participants were recruited. Outline any potential self-selection bias or other biases that may be present and how these are likely to impact results.*
Ethics oversight	*Identify the organization(s) that approved the study protocol.*

Note that full information on the approval of the study protocol must also be provided in the manuscript.

Field-specific reporting

Please select the one below that is the best fit for your research. If you are not sure, read the appropriate sections before making your selection.

☒ Life sciences ☐ Behavioural & social sciences ☐ Ecological, evolutionary & environmental sciences

For a reference copy of the document with all sections, see nature.com/documents/nr-reporting-summary-flat.pdf

Life sciences study design

All studies must disclose on these points even when the disclosure is negative.

Sample size	No statistical method was used to predetermine sample size prior to the experiments. The sample size (e.g., the number of independent biological replicates, n ≥ 3) for each experiment was chosen based on established practices in the field for similar biochemical and molecular biology assays (such as Western blot and in vitro protease activity assays). This sample size has been demonstrated in our preliminary experiments and previous publications to be sufficient to detect consistent and reproducible effects with statistical significance where applicable. Each experiment was repeated independently to ensure the reliability of the findings.
Data exclusions	No data were excluded from the analyses presented in this study. All experimental replicates and data points are included in the reported results.
Replication	To ensure the reproducibility of our findings across all experimental platforms, the following measures were implemented:<br>1. Biochemical Assays (Western Blot, Co-IP):<br>All Western blot and co-immunoprecipitation experiments were performed with a minimum of three independent biological replicates (n ≥ 3). Each replicate was conducted with separately prepared cell transfections and protein lysates on different days. Key findings were confirmed by repeating experiments using freshly prepared reagents and/or different plasmid preparations.<br>2. Enzymatic Kinetics:<br>All kinetic assays were performed in at least three technical replicates, and the entire experiment was repeated in two or more independent runs to ensure consistency. Data points represent the mean ± SD of the replicates.<br>3. Structural Biology:<br>The reproducibility of structural findings is inherent in the data quality and refinement statistics. The final model is based on electron density maps that are supported by the experimental data. The structure was validated using both the PDB validation server.<br><br>All key findings reported in this study, including protein-protein interactions, enzymatic activities, cellular localization, and structural insights, were successfully replicated across all independent experiments. There were no unexplained failures of replication. Any quantitative data derived from these methods were subjected to statistical analysis to confirm consistency.
Randomization	Randomization is not applicable to this study. The experimental groups were defined by the specific plasmid (e.g., encoding a coronavirus

Randomization	main protease or empty vector) used for transfection into the HEK-293T cell line. As an in vitro study using a homogeneous cell population, all cells were considered genetically identical for the purposes of these experiments. Group allocation was therefore based entirely on the experimental transfection administered, not on random assignment. To control for technical variability, transfections for different experimental groups were performed simultaneously, and all samples were processed in parallel for subsequent analyses (e.g., Western blot, activity assays).
Blinding	Blinding was not applicable to this in vitro study. The assessment of outcomes (e.g., protein cleavage by Western blot, NF-κB activity by reporter assay) relied on quantitative measurements obtained by automated instruments or standardized software, which are not susceptible to observer bias. Group allocation was based on plasmid transfection, and all samples were processed identically.

Reporting for specific materials, systems and methods

We require information from authors about some types of materials, experimental systems and methods used in many studies. Here, indicate whether each material, system or method listed is relevant to your study. If you are not sure if a list item applies to your research, read the appropriate section before selecting a response.

Materials & experimental systems

n/a	Involved in the study
☐	☒ Antibodies
☐	☒ Eukaryotic cell lines
☒	☐ Palaeontology and archaeology
☒	☐ Animals and other organisms
☒	☐ Clinical data
☒	☐ Dual use research of concern
☒	☐ Plants

Methods

n/a	Involved in the study
☒	☐ ChIP-seq
☒	☐ Flow cytometry
☒	☐ MRI-based neuroimaging

Antibodies

Antibodies used	Primary Antibodies:<br>Anti-FLAG: Supplier 1: Sigma-Aldrich (Catalog #F7425; Clone M2). Supplier 2: Cell Signaling Technology (CST) (Catalog #14793; Clone D6W5B).<br>Anti-Myc: Cell Signaling Technology (CST) (Catalog #18583; Clone 71D10).<br>Anti-GAPDH: Cell Signaling Technology (CST) (Catalog #5174; Clone D16H11).<br>Anti-GFP: Cell Signaling Technology (CST)(Catalog #2956; Clone D5.1)<br>Secondary Antibodies:<br>Anti-rabbit IgG, HRP-linked: Cell Signaling Technology (CST) (Catalog #7074).<br>Anti-mouse IgG, HRP-linked: Cell Signaling Technology (CST) (Catalog #7076).
Validation	Validation for each primary antibody is described below, based on validation data provided by the manufacturers (Cell Signaling Technology and Sigma-Aldrich) for use in Western blot (WB) applications.<br><br>Anti-FLAG (Sigma-Aldrich F7425 & CST 14793):Both antibodies are validated by the manufacturers for detection of recombinant FLAG-tagged proteins in Western blotting. Specific validation data, including Western blot results, are provided on the manufacturers' product web pages.<br>Anti-Myc (CST 18583): Validated by CST for specific detection of recombinant Myc-tagged proteins in Western blot applications. Validation data is available on the CST website.<br>Anti-GFP (CST 2956): Validated by CST for detection of GFP-tagged proteins. According to the manufacturer's validation data, this antibody is expected to recognize GFP, eGFP, and YFP, and is suitable for Western blotting.<br>Anti-GAPDH (CST 5174): Validated by CST for detecting endogenous levels of GAPDH as a loading control in Western blot. Specificity is confirmed by the manufacturer.<br><br>The secondary antibodies (Anti-rabbit IgG, HRP-linked #7074 and Anti-mouse IgG, HRP-linked #7076) are highly cross-adsorbed and validated for use with the corresponding host species' primary antibodies in Western blot.

Eukaryotic cell lines

Policy information about cell lines and Sex and Gender in Research

Cell line source(s)	The HEK-293 cell line used in this study was obtained from the American Type Culture Collection. This cell line is derived from the kidney tissue of a female human embryo.
Authentication	None of the cell lines used in this study, including HEK-293T, were specifically authenticated for this project. However, the cell line was obtained directly from a reputable cell bank (e.g., ATCC) and used at a low passage number to minimize the risk of misidentification.
Mycoplasma contamination	All cell lines used, including HEK-293T, have tested negative for mycoplasma contamination.

Commonly misidentified lines
(See ICLAC register)

No commonly misidentified cell lines, as listed by the ICLAC, were used in this study.

Plants

Seed stocks

Report on the source of all seed stocks or other plant material used. If applicable, state the seed stock centre and catalogue number. If plant specimens were collected from the field, describe the collection location, date and sampling procedures.

Novel plant genotypes

Describe the methods by which all novel plant genotypes were produced. This includes those generated by transgenic approaches, gene editing, chemical/radiation-based mutagenesis and hybridization. For transgenic lines, describe the transformation method, the number of independent lines analyzed and the generation upon which experiments were performed. For gene-edited lines, describe the editor used, the endogenous sequence targeted for editing, the targeting guide RNA sequence (if applicable) and how the editor was applied.

Authentication

Describe any authentication procedures for each seed stock used or novel genotype generated. Describe any experiments used to assess the effect of a mutation and, where applicable, how potential secondary effects (e.g. second site T-DNA insertions, mosiacism, off-target gene editing) were examined.
