# Supplementary material for: The seven human coronavirus main proteases exhibit differential modulation of NF-κB suppression via TAB1 cleavage
Source: Commun Biol. 2026 May 21;9:1087. doi: 10.1038/s42003-026-10225-9 (PMC13462985; doi:10.1038/s42003-026-10225-9)
Supplement: Supplementary file 4 [file 42003_2026_10225_MOESM5_ESM.pdf]

nature portfolio

Peer Review File

The seven human coronavirus main proteases exhibit differential modulation of NF-κB suppression via TAB1 cleavage

Corresponding Author: Professor Hao Huang

This file contains all reviewer reports in order by version, followed by all author rebuttals in order by version.

Version 0:

Reviewer comments:

Reviewer #1

(Remarks to the Author)
Overall, this is a very good paper that clearly shows the cleavage of TAB1 by coronavirus MPro, with molecular detail and also differential cleavage by different coronaviruses.

There are a couple of points it would be nice to get clarification on (or suggestions to improve the paper):
1. Figure 1: it is odd that panels A and C are next to each other, rather than following A,B, then C.
2. Figure 1, panels D and E: unclear why ponceau staining was used instead of Western blot for MPro
3. Figure 2D: there is c an apparent cleavage band in the Q10AQ44A lane - despite a claim that cleavage is completely blocked
4. Figure 3: Is it possible to determine whether the 445-504 fragment still binds TAK1 (as implied in 3G) - this may be the mechanism, as this might then block further binding of full-length TAB1?
5. Figure 3D and 3E appear to the same output, can these be merged together?
6. Figure 4: Can the mutants from C and D be used in the luciferase assay in G? This would show a more direct link between cleavage and pathogenicity - which at the moment is (as acknowledged by the authors) quite speculative (based on correlation)? Or else, it may be worth toning down (or removing) the link to pathogenicity as an output (I don't think this is really needed).

Stylistic points:
1. Inconsistent labelling style across figures - e.g. sometime using arrows and sometimes diamonds to label bands.
2. Random labels - such as "in vitro" under figures. These can be removed.

Overall - I think minor corrections are required.

Reviewer #2

(Remarks to the Author)
While previous studies have reported the cleavage of TAB1 by Mpro, I still appreciate the authors' systematic comparative analysis of the cleavage efficiency of TAB1 by Mpro proteins from 7 human coronaviruses. Overall, the quality of the data in the manuscript needs improvement, with specific issues outlined below.
1. FIG 1A
Flag-TAB1 was able to pull down the mutant GFP-Mpro C145A, but not GFP-Mpro itself. How did the authors conclude that "Co-immunoprecipitation (Co-IP) assays confirmed a direct physical interaction between SARS-CoV-2 Mpro and TAB1"? Additionally, no information about the GFP-Mpro C145A mutant was mentioned in the main text at this point. Furthermore, the interaction between GFP-Mpro C145A and TAB1 contradicts the lack of colocalization between GFP-Mpro C145A and TAB1 in FIG 1B (the colocalization data itself is also confusing, as described below). Given the focus of the manuscript, I suspect that the authors may have mislabeled the lanes corresponding to Mpro and Mpro C145A in FIG 1A. If this was the case, Mpro would interact with TAB1, while the Mpro C145A mutant would not. However, this speculation is inconsistent with subsequent content in Fig5: if the Mpro C145A mutant does not interact with TAB1, why was the structure of the complex formed by this inactive mutant and the TAB1 peptide detected (Fig 5)?

2. FIG 1B
The GFP-Mpro fusion protein and TAB1-Flag were distributed in both the cytoplasm and nucleus, with seemingly stronger signals in the nucleus. Is this consistent with the inherent localization of viral Mpro and endogenous TAB1? Moreover, the colocalization of GFP-Mpro and the TAB1-Flag fusion protein appeared to be concentrated in the nucleus (the area indicated by the blue dye), rather than in the cytoplasm as described by the authors. Given the differences between overexpressed tagged proteins and endogenous proteins, it is also necessary to verify the interaction and colocalization between Mpro and endogenous TAB1 under viral infection conditions. Additionally, the images are blurry and the magnification is insufficient to observe the detailed features of colocalization. Additionally, no information regarding the GFP-Mpro C145A mutant and TAB1 is provided here (the images in the fourth row are not described at all).

3. The authors consistently used GFP-fused TAB to study the cleavage of TAB by Mpro. Given that WB analysis relies on observing protein molecular weights to determine cleavage, Fig 2B should show the molecular weights of the truncated fragments of the GFP-TAB fusion protein generated after cleavage at different cleavage sites, to facilitate readers' interpretation of the WB data regarding cleavage.

4. If the single-site mutation in GFP-Mpro C145A affects its interaction and colocalization with TAB1 (Fig 1), does the Q-to-A mutant of TAB1 affect the interaction between TAB1 and Mpro? If such an impact exists, Mpro would not be able to cleave the TAB1 Q-A mutants under these circumstances, making it impossible to conclude that Q is a key cleavage site. Therefore, Fig 2 needs to supplement experiments to verify the binding between Mpro and the indicated Q-to-A mutants of TAB1.

5. In FIG 3A-B, "NF-κB" refers to the reporter plasmid for NF-κB promoter, which differs in nature from TAB1/TAK1. This should be clearly labeled or unlabeled (it was unlabeled in FIG 3C-D), otherwise, it may cause confusion (For instance, it led me to mistakenly assume that the expression plasmids for NF-κB subunits were also co-transfected). Additionally, in the results presented by the authors, NF-κB signaling is not activated when TAB or TAK is overexpressed alone, but activated when co-expression. Are there similar literature reports to support this finding? Or are there other alternative ways to activate NF-κB?

6. Given that the cleavage of TAB1 by Mpro has been previously reported, and reporter gene assays may have certain inaccuracies, the authors should consider supplementing experiments to detect the inhibitory effect of Mpro expression on key events in the NF-κB signaling pathway (such as IκB phosphorylation and degradation, as well as p65 phosphorylation and nuclear translocation).

7. "P" value is missing in FIG 3B, and the labeling of statistical significance in the bar graphs throughout the manuscript is incorrect.

8. In the presence of Mpro, both termini of TAB1—including its N-terminal Flag tag—undergo cleavage. Consequently, when FLAG-TAB1 is used as the bait protein, the abundance of intact Flag-TAB1 is diminished as a direct result of Mpro-mediated cleavage; this reduction, in turn, lowers the likelihood of precipitating TAK1 (a TAB1-interacting protein). As such, the data cannot substantiate the conclusion that "Mpro markedly reduced TAB1–TAK1 binding".

9. Is there a statistical difference in the inhibitory effect on the NF-κB promoter between Mpro from low-pathogenic coronaviruses and that from high-pathogenic coronaviruses? Additionally, similar to the issue mentioned above, the presentation of statistical analysis in this figure is incorrect.

10. The substrate peptide (TAB1 residues 441-448) exhibits high affinity for SARS-CoV-2 Mpro. Can this peptide act as a competitive binder of Mpro to inhibit Mpro-mediated cleavage of TAB1, thereby suppressing SARS-CoV-2 infection?

11. In line L316 of the Discussion, the claim is overstated. No data supporting a "complete shutdown of the NF-κB signaling pathway" was observed.

Reviewer #3

(Remarks to the Author)
In this manuscript, Fu et al. attempt to address the question of how CoV Mpros cleave the innate immune protein TAB1. They provide evidence that TAB1 is a conserved substrate for seven human coronavirus (HCoV) Mpros. Specifically, Mpros from SARS-CoV-2, SARS-CoV, and MERS cleave TAB1 at two distinct sites (Q10 and Q444), while Mpros from HCoV-OC43, HCoV-HKU1, HCoV-NL63, and HCoV-229E primarily cleave at Q10. This cleavage likely disrupts the TAB1-TAK1 complex, thereby blocking downstream signaling. This study builds on a previous report that demonstrated SARS-CoV-2 Mpro can cleave TAB1 at Q132 and Q444. Overall, this work offers novel and comprehensive insights that are useful to the field.

Concerns:
1. Line 169: "Interestingly, mutation of the Q10 site led the Mpro of HCoV-OC43, -229E, -NL63, and -HKU1 to adaptively cleave at the Q444 site, producing high-MW cleavage bands similar to those of Mpro from SARS-CoV-2, SARS-CoV, and MERS (Fig. 4C)." It is confusing why Q10 mutation causes Mpros from low-pathogenicity viruses to cleave at Q444 site as the authors previously claim they “selectively cleaved the Q10 site, yielding a single low-MW fragment (Fig. 4B)“ in WT TAB1. The authors should repeat this experiment and provide a reasonable explanation for the observed discrepancy. To

address this issue more effectively, I suggest the authors synthesize a fluorescent-labeled peptide derived from the Q10 region, test the enzyme activity, and compare the results with those obtained from Q444.

2. The authors determined the structure of SARS-CoV-2 Mpro C145A in complex with human TAB1 (residues 441–448) at a resolution of 3.42 Å. In comparison with hundreds of high-resolution Mpro structures deposited in the PDB, this resolution is relatively low and may not provide sufficient detail for a thorough structural analysis. The authors should consider optimizing the crystals and improving the resolution to obtain more precise structural information.

3. Given the low-resolution crystal structure, the discussion in the section titled "Comparison of the Mpro and TAB1 Structures of Seven Human Coronaviruses" is not convincing for detailed structural comparison.

Version 1:

Reviewer comments:

Reviewer #1

(Remarks to the Author)
The authors have responded very well to all of the previous comments and I do not see any reason that this can now not be accepted.

I make two minor points:
1. While I appreciate that they do not have an antibody to Mpro - they do exist, so I feel the Ponceau stain in figure 1 could be replaced.
2. The authors state that the Ponceau shows equal loading - however, there is clearly less protein present in the mutant version than the wild-type in figure 1D

However, given all of the other evidence of the interaction they propose - these are minor and not essential to fix. I would almost be tempted to remove the Ponceau stain - though I do appreciate the need for it

Reviewer #2

(Remarks to the Author)
Regarding the revised version, I consider it necessary to repeat some of the Mpro–TAB1 interaction data. Moreover, given that TAB1 antibodies are commercially available, it would be advisable to detect TAB1 using antibodies against the protein (polyclonal antibodies recognizing multiple epitopes might be more appropriate) rather than relying on tag antibodies,given that the N terminal tag is cleaved off.
Specific comments:
1 Considering the transient nature of many enzyme-substrate interactions, the authors' rebuttal could explain why uncleaved FLAG-TAB1 fails to pull down Mpro. However, I still have concerns regarding Figure 1A. First, why are cleaved TAB1 fragments detected by the FLAG antibody (two bands) in the input samples, but only the full-length form (a single band) remains in the pull-down (PD) samples? Second, there is a serious inconsistency between the protein amounts in the input and the pull-down: the group with the highest input shows the least amount pulled down. Furthermore, comparable—if not higher—levels of full-length TAB1 were enriched in the wild-type group (where TAB1 cleavage occurs) compared to the mutant group (where no cleavage occurs).

2 Considering that the N-terminal FLAG tag on FLAG-TAB1 is cleaved by Mpro, using a FLAG antibody to represent TAB1 localization in Figure 1C is also inaccurate. Moreover, given the transient nature of the enzyme-substrate intermediate complex, live-cell dynamic imaging might be more suitable than imaging fixed samples.

3 In Figure 2E, the Q10A mutation does not show a substantial reduction in the Mpro-TAB1 interaction. This contradicts the authors' description: "Mutation of either Q10 or Q444 significantly weakened the binding affinity between TAB1 and Mpro C145A. Moreover, the Q10A/Q444A double mutant failed to co-immunoprecipitate with Mpro C145A, indicating a complete loss of binding (Fig. 2E). Collectively, these data establish Q10 and Q444 as critical residues on TAB1 for both recognition and cleavage by SARS-CoV-2 Mpro."

4 In Figure 3F, the use of an N-terminally tagged TAB1 (which is cleaved by Mpro) likely overestimates the effect of Mpro cleavage at the TAB1 C-terminus on its binding to TAK1. Although the authors touched on this point in the rebuttal, it is not reflected in the main text. Perhaps an alternative approach would be to insert the tag after the Q10 site, or to use Q10 mutants, to exclude the influence of TAB1 cleavage at the Q10 site on this experiment.

Reviewer #3

(Remarks to the Author)
The authors have addressed the reviewer's concerns.

Open Access This Peer Review File is licensed under a Creative Commons Attribution 4.0 International License, which permits use, sharing, adaptation, distribution and reproduction in any medium or format, as long as you give appropriate credit to the original author(s) and the source, provide a link to the Creative Commons license, and indicate if changes were made.
In cases where reviewers are anonymous, credit should be given to 'Anonymous Referee' and the source.
The images or other third party material in this Peer Review File are included in the article's Creative Commons license, unless indicated otherwise in a credit line to the material. If material is not included in the article's Creative Commons license and your intended use is not permitted by statutory regulation or exceeds the permitted use, you will need to obtain permission directly from the copyright holder.
To view a copy of this license, visit https://creativecommons.org/licenses/by/4.0/

PEKING UNIVERSITY

1299 LiShui Road F-401, Shenzhen University Town
Xili, Nanshan District, Shenzhen, Guangdong, China
Tel:+86-0755-2603-2321
Email: huang.hao@pku.edu.cn
http://web.pkusz.edu.cn/huanghao/

Manuscript ID: COMMSBIO-25-10053A

Title: The seven human coronavirus main proteases exhibit differential modulation of NF-κB suppression via TAB1 cleavage

Dear Editors and Reviewers,

We appreciate the opportunity to revise our manuscript and are grateful for the insightful comments provided by the editor and reviewers. In the following, we have provided point-by-point responses to each of the reviewers' comments. In this response letter, the reviewers' comments are presented in italics, and our corresponding changes and additions to the manuscript are highlighted in blue text.

Reviewre#1:

Overall, this is a very good paper that clearly shows the cleavage of TAB1 by coronavirus MPro, with molecular detail and also differential cleavage by different coronaviruses.

Response: We thank Reviewer 1 for the positive comment on our work.

Comment 1: *Figure 1: it is odd that panels A and C are next to each other, rather than following A, B, then C.*

Response 1: We thank Reviewer 1 for this helpful suggestion. In the revised manuscript, we have rearranged the figure panels accordingly.

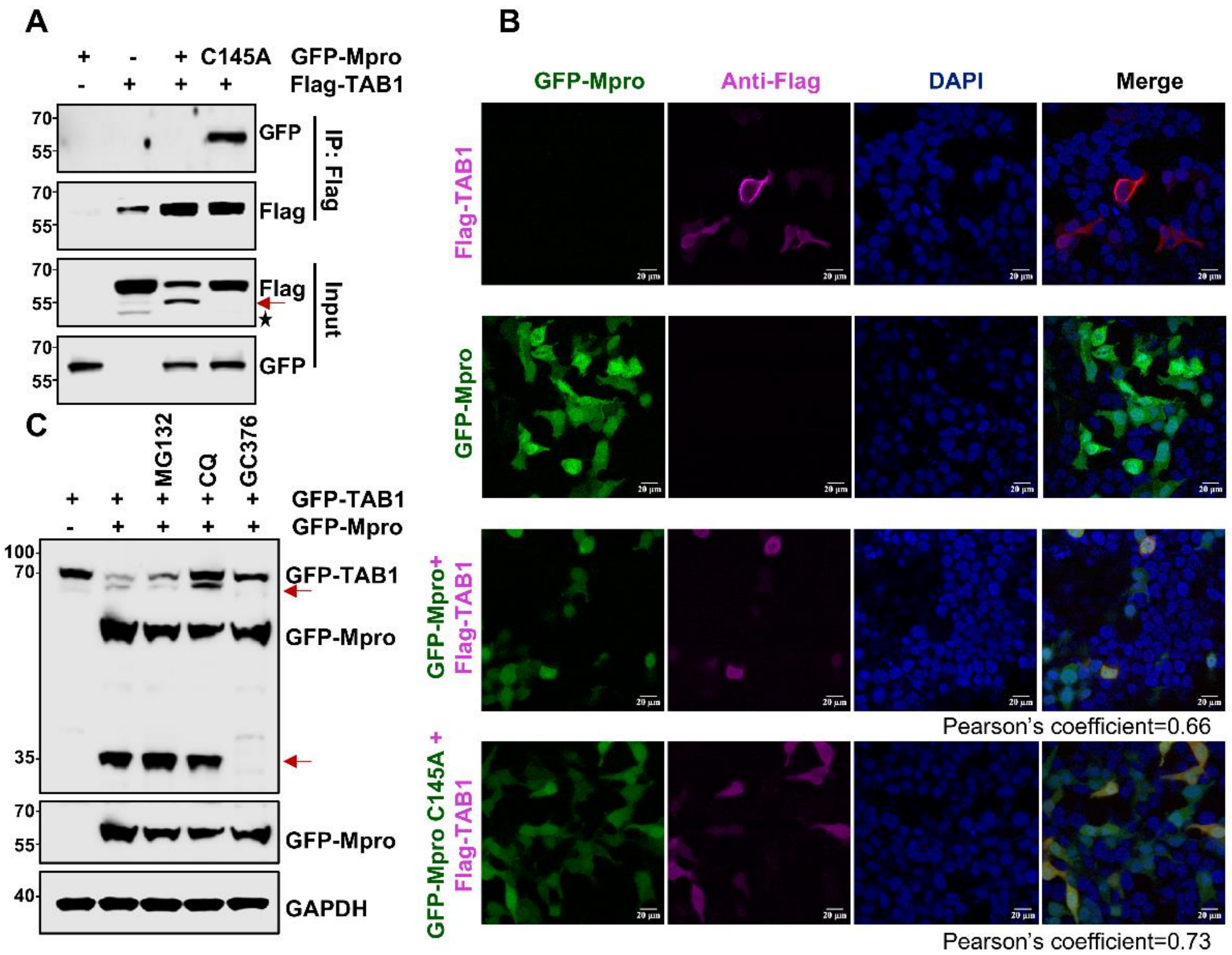

Fig. 1A: Co-immunoprecipitation of SARS-CoV-2 Mpro/C145A and TAB1

Fig. 1B: Intracellular colocalization of SARS-CoV-2 Mpro/C145A and TAB1.

Fig. 1C: Catalytic activity-dependent cleavage of TAB1 by SARS-CoV-2 Mpro.

Comment 2: *Figure 1, panels D and E: unclear why ponceau staining was used instead of Western blot for Mpro.*

Response 2: We thank Reviewer 1 for this question. In the in vitro cleavage assays (Figure 1D and 1E), purified recombinant SARS-CoV-2 Mpro was used. Since we did not have a specific antibody suitable for quantitative detection of this recombinant Mpro, we employed Ponceau S staining as a standard and direct method to verify equal protein loading across all reaction lanes. This ensures that the observed cleavage differences in TAB1 are either due to different concentrations of Mpro or Mpro C145A mutation.

Comment 3: *Figure 2D: there is c an apparent cleavage band in the Q10AQ44A lane - despite*

a claim that cleavage is completely blocked.

Response 3: Thank you for this careful observation regarding Figure 2D. The faint, higher molecular weight band is a non-specific signal, not a cleavage product, as it is also present in the control sample (lane 2) where no Mpro is present. We have separately labeled the non-specific bands and the cleavage products in revised Figure 2D.

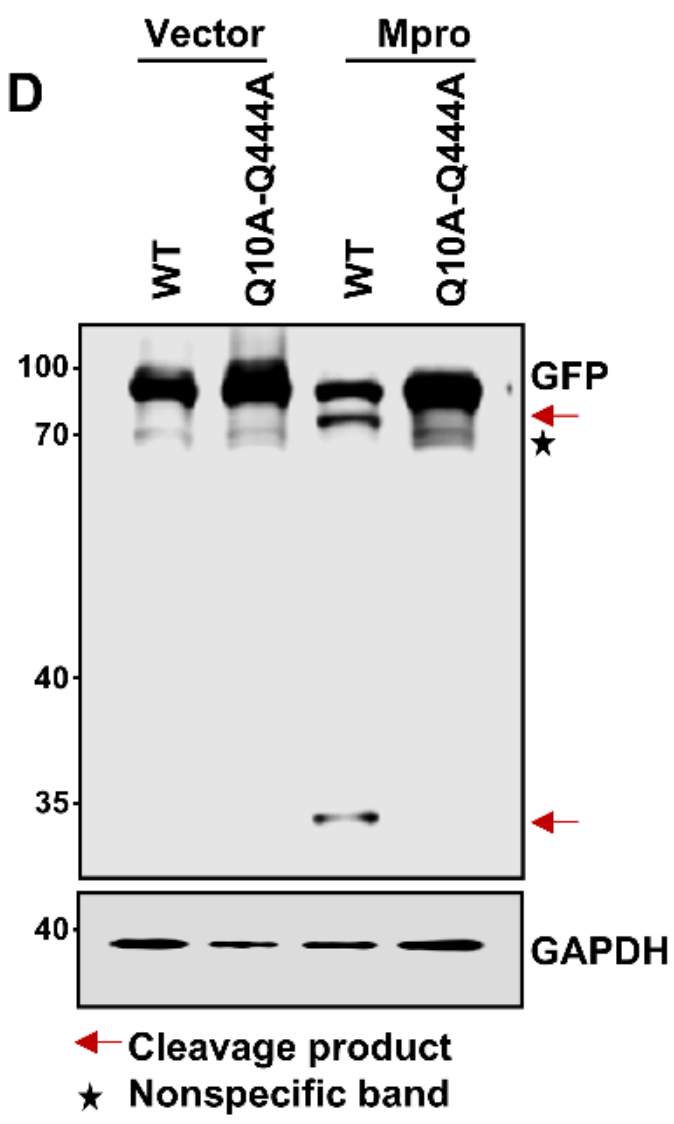

Fig. 2D: Essential role of Q444 and Q10 in TAB1 processing.

Comment 4: *Figure 3: Is it possible to determine whether the 445-504 fragment still binds TAK1 (as implied in 3G) - this may be the mechanism, as this might then block further binding of full-length TAB1?*

Response 4: Thank you for raising this insightful question regarding the potential mechanistic role of the C-terminal *445-504* fragment. To directly test whether the 445-504 fragment retains TAK1-binding ability, we additionally performed co-immunoprecipitation (co-IP) experiments using a panel of TAB1 truncation constructs.

Our results (new Figure 3E) clearly show that the 445-504 fragment efficiently binds TAK1, comparable to FL-TAB1 and the larger C-terminal fragment 11-504. In contrast, the N-terminal

fragments (1-10 and 1-444) showed no detectable interactions. This finding is consistent with the previously mapped TAK1-binding domain (residues 437–504) (PMID: 28106845) and confirms that the Mpro-cleaved product 445-504 fragment is fully capable of interacting with TAK1.

Therefore, as Reviewer 1 suggests, a plausible mechanism emerges: the released 445-504 fragment may competitively occupy the TAK1-binding interface, thereby preventing recruitment of FL-TAB1 and subsequent activation of the TAK1/NF-κB pathway. We have incorporated these new data (new Fig 3E and 3G) and the proposed mechanism [lines 163-166, and 172-173 in the revised manuscript] into the revised manuscript.

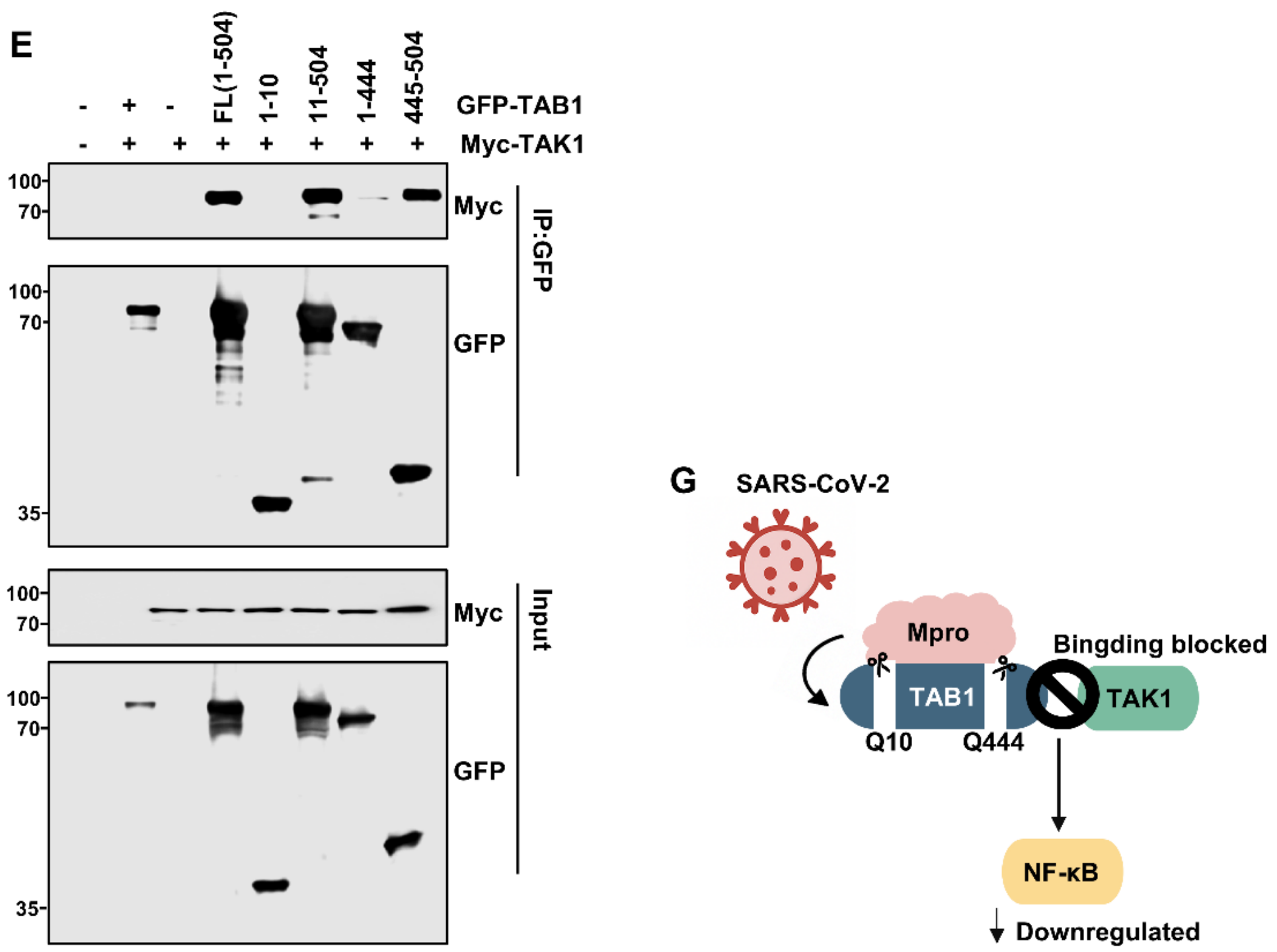

Fig. 3E: The binding of the truncated TAB1 fragments to TAK1.

Fig. 3G: The model of Mpro-mediated disruption of TAB1-TAK1 complex.

Comment 5: *Figure 3D and 3E appear to the same output, can these be merged together?*

Response 5: We thank the Reviewer for the suggestion. Accordingly, we re-conducted the experiment to fit the data to a panel (revised Fig. 3D), which provides a clearer and more concise visualization of the results.

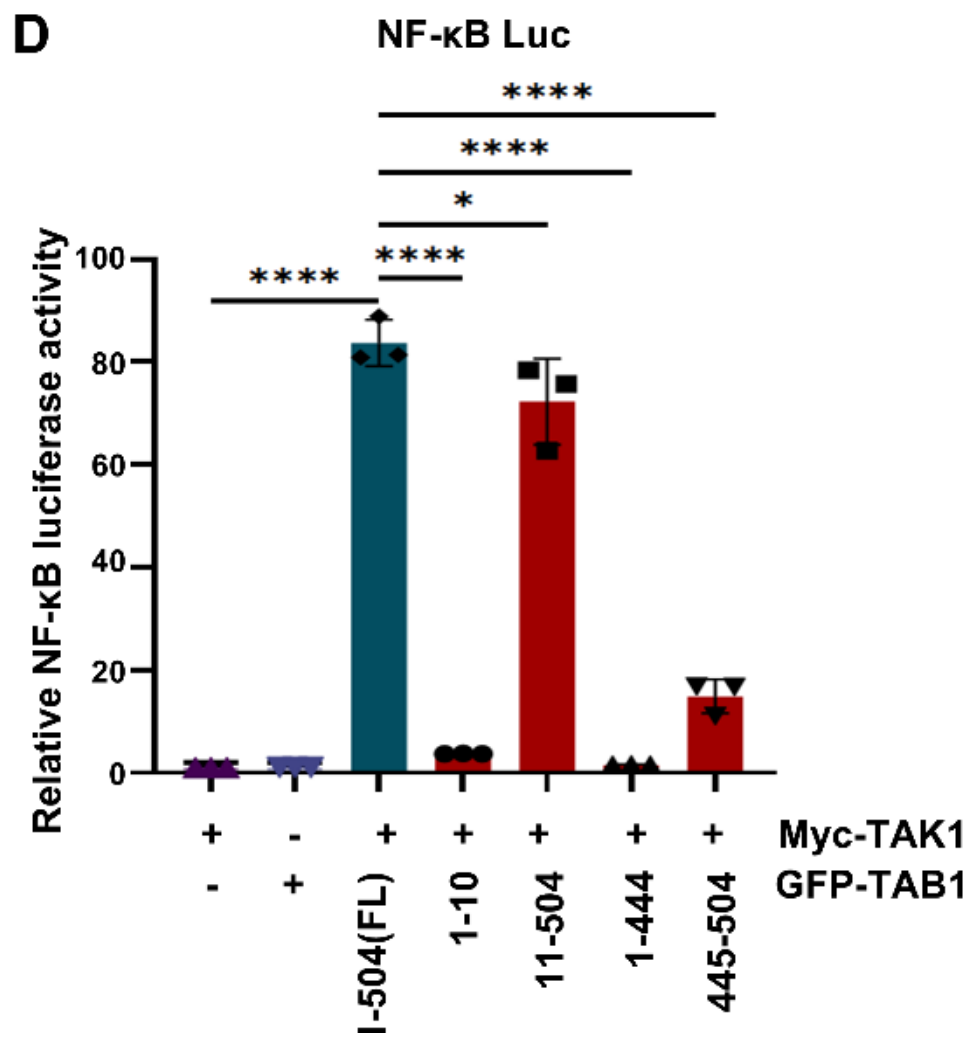

Fig. 2D: Truncated TAB1 mutants show impaired ability to activate NF-κB signaling.

Comment 6: *Figure 4: Can the mutants from C and D be used in the luciferase assay in G? This would show a more direct link between cleavage and pathogenicity - which at the moment is (as acknowledged by the authors) quite speculative (based on correlation)? Or else, it may be worth toning down (or removing) the link to pathogenicity as an output (I don't think this is really needed).*

Response 6: We thank the Reviewer for raising this important point regarding the link between TAB1 cleavage and viral pathogenicity. In line with Reviewer 1's suggestion, we have chosen to significantly tone down the narrative regarding pathogenicity in the revised manuscript. Throughout the revised manuscript, we have replaced the term 'high pathogenicity' with 'severe respiratory coronaviruses' (SARS-CoV-2, SARS-CoV, and MERS-CoV). Similarly, we have replaced 'low pathogenicity' with 'seasonal human coronaviruses' (HCoV-OC43, HCoV-HKU1, HCoV-NL63, and HCoV-229E). We believe this nomenclature is more accurate.

In addition, we conducted the new experiments suggested by Reviewer 1: we used cleavage-resistant TAB1 mutants in the luciferase assay. As newly presented in Fig. 4G-J: WT-TAB1-driven NF-κB activation is suppressed by all seven coronaviral Mpros (Fig. 4G). Single-site mutants (Q10A or Q444A) only partially rescue the NF-κB signaling, as Mpros can still cleave at the remaining site (Fig. 4G-I). Critically, the double mutant TAB1-Q10A/Q444A,

which is completely resistant to cleavage fully abrogates the ability of every tested Mpro to inhibit NF-κB activation (Fig. 4J). Thess new experiments were descried in line 204-209 in the revised manuscript.

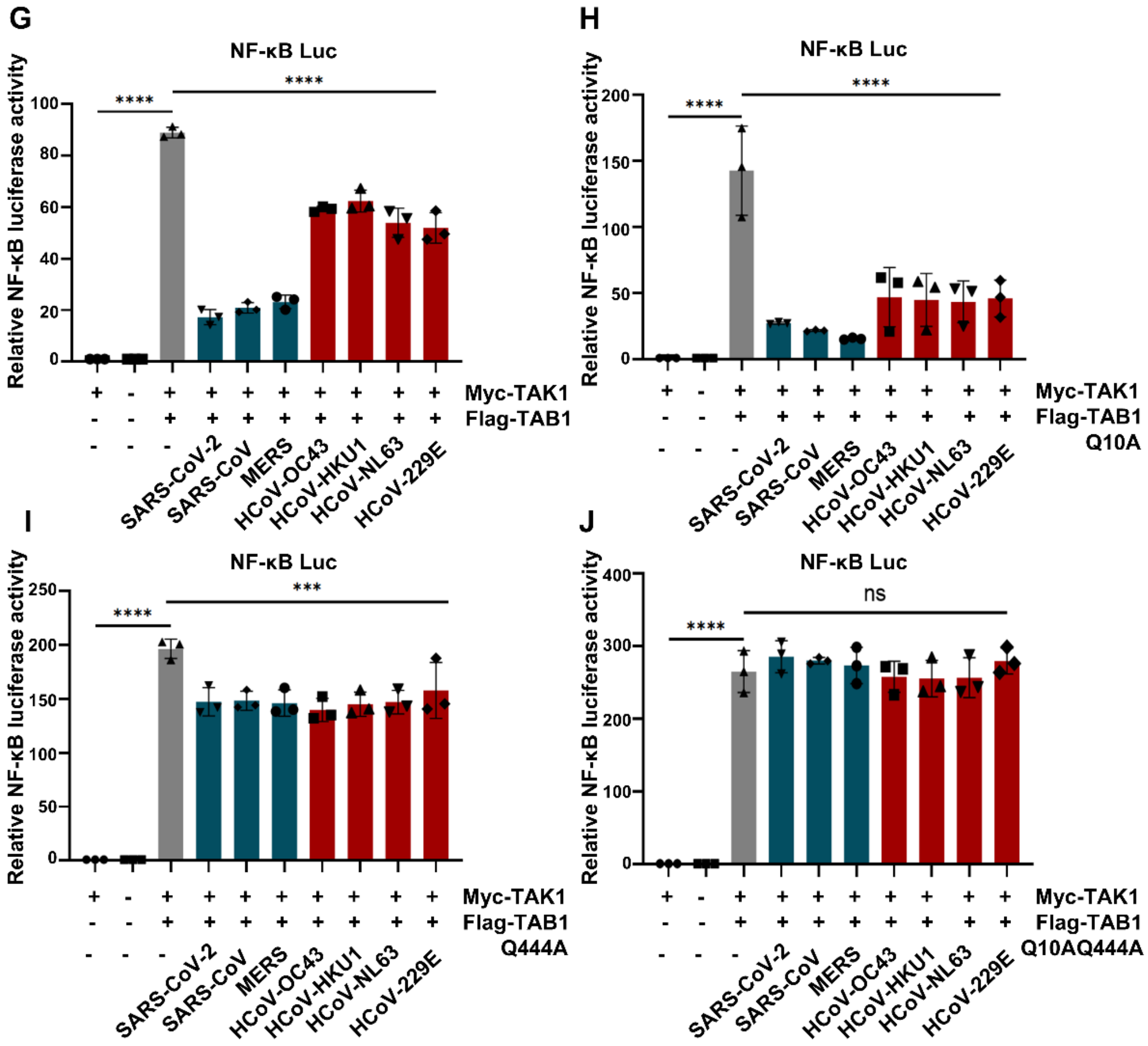

Fig. 4G-J: Inhibition of TAB1/WT/Q10A/Q444A/Q10A-Q444Amutant-TAK1-induced NF-κB activation by seven coronaviral Mpro.

Comment 7: *Stylistic points: Inconsistent labelling style across figures - e.g. sometime using arrows and sometimes diamonds to label bands.*

Response 7: We thank the Reviewer for pointing out the inconsistency in labelling style across the figures. We have carefully reviewed all figure panels and have unified symbols as arrows to indicate protein bands.

Comment 8: *Stylistic points: Random labels - such as "in vitro" under figures. These can be removed.*

Response 8: We thank the Reviewer for highlighting this inconsistency. We have reviewed all figure labels and removed the extraneous descriptors, such as "*in vitro*," from the figure panels.

Overall - I think minor corrections are required.

Reviewer #2

While previous studies have reported the cleavage of TAB1 by Mpro, I still appreciate the authors' systematic comparative analysis of the cleavage efficiency of TAB1 by Mpro proteins from 7 human coronaviruses. Overall, the quality of the data in the manuscript needs improvement, with specific issues outlined below.

Response: We appreciate the Reviewer's critical assessment and helpful suggestions and comments.

Comment 1: *FIG 1A. Flag-TAB1 was able to pull down the mutant GFP-Mpro C145A, but not GFP-Mpro itself. How did the authors conclude that "Co-immunoprecipitation (Co-IP) assays confirmed a direct physical interaction between SARS-CoV-2 Mpro and TAB1"? Additionally, no information about the GFP-Mpro C145A mutant was mentioned in the main text at this point. Furthermore, the interaction between GFP-Mpro C145A and TAB1 contradicts the lack of colocalization between GFP-Mpro C145A and TAB1 in FIG 1B (the colocalization data itself is also confusing, as described below). Given the focus of the manuscript, I suspect that the authors may have mislabeled the lanes corresponding to Mpro and Mpro C145A in FIG 1A. If this was the case, Mpro would interact with TAB1, while the Mpro C145A mutant would not. However, this speculation is inconsistent with subsequent content in Fig5: if the Mpro C145A mutant does not interact with TAB1, why was the structure of the complex formed by this inactive mutant and the TAB1 peptide detected (Fig 5)?*

Response 1: We sincerely thank the reviewer for carefully reading our manuscript and raising these important points.

First, Mpro C145A is a catalytically inactive mutant of Mpro. We added this information to the revised manuscript, line 91-92.

Second, WT-Mpro functions as a protease that binds and efficiently cleaves TAB1; this cleavage causes the complex to dissociate, resulting in the loss of FL-TAB1 and the apparent

absence of interaction in the Co-IP assay. In contrast, Mpro C145A is catalytically inactive. It retains the structural ability to bind the TAB1 substrate but cannot cleave it. This ‘substrate trapping’ allows the Mpro-TAB1 complex to remain stable and intact during the immunoprecipitation process, thereby confirming the physical interaction between the two proteins.

Third, as reviewer 2 notes that the co-localization image in Fig. 1B appears to show signal for both WT-Mpro and Mpro C145A with TAB1. This observation is consistent with the ability of both proteins to interact with TAB1 in cellular context.

Fourth, the structural study in Fig. 5 used the Mpro C145A mutant in complex with a TAB1-derived peptide. This is a common practice in protease–substrate structural studies, as the catalytically inactive mutant stabilizes the bound state and avoids cleavage during crystallization (PMID: 36075915; PMID: 27799534; PMID: 35380892; PMID: 37609788)

The use of the same mutant in both the co-IP and the structural analysis is therefore methodologically consistent and reinforces the conclusion that Mpro directly binds the cleavage region of TAB1.

We apologize for any confusion caused by the initial presentation and thank the reviewer for prompting us to clarify these critical points.

Comment 2: *FIG 1B. The GFP-Mpro fusion protein and TAB1-Flag were distributed in both the cytoplasm and nucleus, with seemingly stronger signals in the nucleus. Is this consistent with the inherent localization of viral Mpro and endogenous TAB1? Moreover, the colocalization of GFP-Mpro and the TAB1-Flag fusion protein appeared to be concentrated in the nucleus (the area indicated by the blue dye), rather than in the cytoplasm as described by the authors. Given the differences between overexpressed tagged proteins and endogenous proteins, it is also necessary to verify the interaction and colocalization between Mpro and endogenous TAB1 under viral infection conditions. Additionally, the images are blurry and the magnification is insufficient to observe the detailed features of colocalization. Additionally, no*

information regarding the GFP-Mpro C145A mutant and TAB1 is provided here (the images in the fourth row are not described at all).

Response 2: We sincerely thank the reviewer for insightful suggestions. To address this concern, we strengthened the manuscript in the following three ways:

1) We have replaced Figure 1B with newly obtained high-resolution confocal images to address the clarity issue. We also explicitly included and analyzed the GFP-Mpro C145A mutant as requested. In addition, we performed quantitative co-localization analysis. The results show high Pearson's correlation coefficients for both Mpro-WT (~0.65) and Mpro-C145A (~0.78) with TAB1, confirming a significant co-localization rather than random overlap.

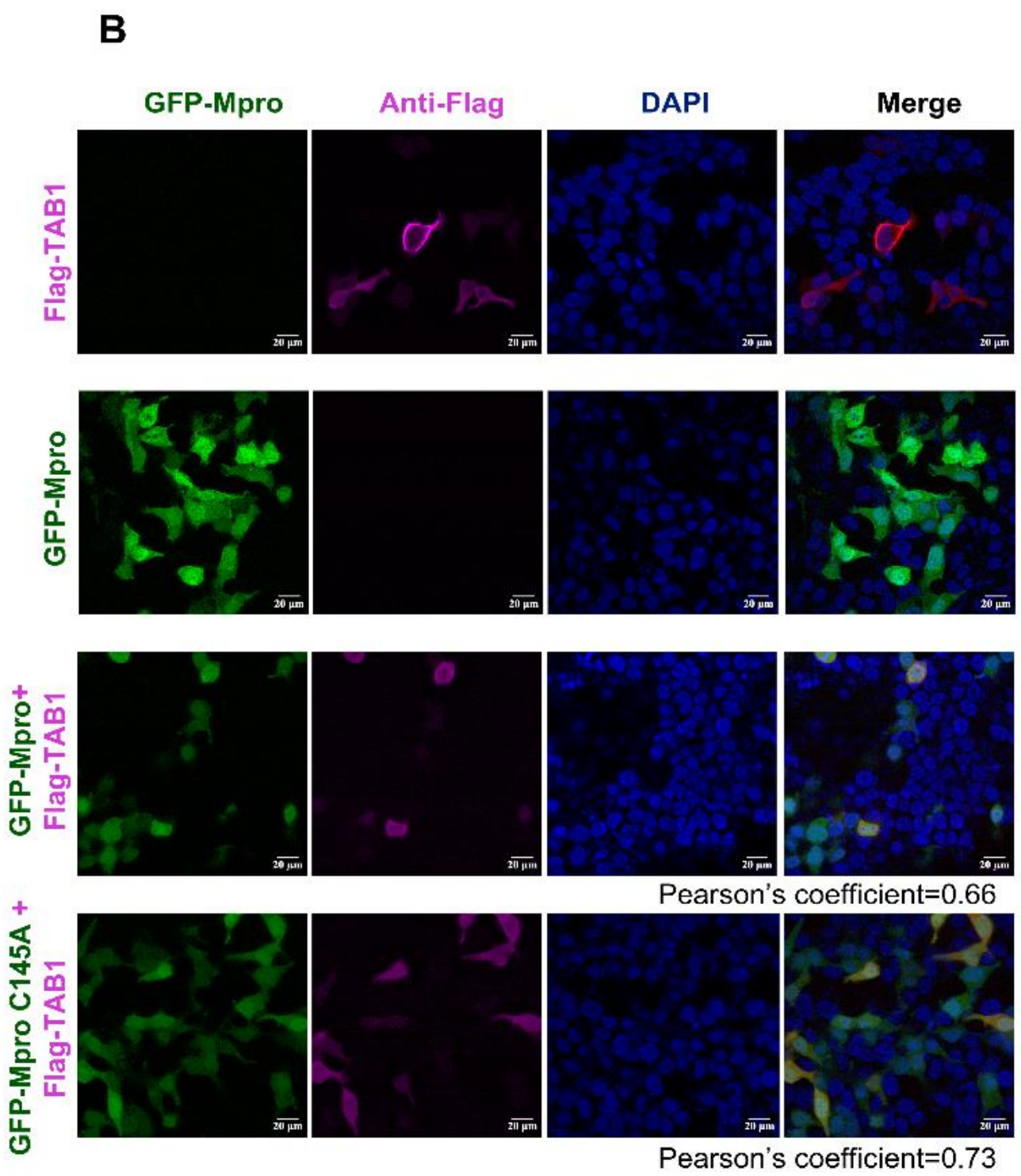

Fig. 1B: Intracellular colocalization of SARS-CoV-2 Mpro/C145A and TAB1.

2) Regarding the signal in both the nucleus and cytoplasm, previous studies have confirmed that SARS-CoV-2 Mpro is distributed in both compartments (PMID: 33986250; PMID: 35075101) and can influence the subcellular localization of host proteins (PMID: 41289387; PMID: 35654983; PMID: 36853058). Our observation of Mpro/TAB1

colocalization in both the nucleus and cytoplasm is consistent with these reported features of Mpro.

3) Following your suggestion, we added the description of the fourth row for Mpro C145A and TAB1 in the revised manuscript (line 96-98): '*Indeed, indirect immunofluorescence microscopy showed pronounced cytoplasmic and nuclear colocalization of GFP-WT-Mpro, GFP-Mpro-C145A with Flag-TAB1 (Fig. 1B), supporting their interaction in a cellular context.*'

We appreciate the reviewer's comments, which have significantly strengthened the rigor and clarity of this figure. Regarding the suggested co-localization experiment under viral infection conditions, which requires a Biosafety Level 3 (BSL-3) Lab or above equipped with confocal microscope, we don't have access to this kind of facilities at this time. Besides, the above mentioned Mpro and substrates colocalization experiments in literature were all done with overexpression of SARS-CoV-2 Mpro (PMID: 41289387; PMID: 35654983; PMID: 36853058) to achieve high enough protein levels for clear visualization.

Comment 3: *The authors consistently used GFP-fused TAB to study the cleavage of TAB by Mpro. Given that WB analysis relies on observing protein molecular weights to determine cleavage, Fig 2B should show the molecular weights of the truncated fragments of the GFP-TAB fusion protein generated after cleavage at different cleavage sites, to facilitate readers' interpretation of the WB data regarding cleavage.*

Response 3: We thank the reviewer for this helpful suggestion. In the revised Figure 2B, we have now explicitly labeled the sizes of both the FL-GFP-TAB1 fusion protein and its expected cleavage products corresponding to the Q10 and Q444 sites. This addition directly addresses your point and will aid readers in following the cleavage patterns presented.

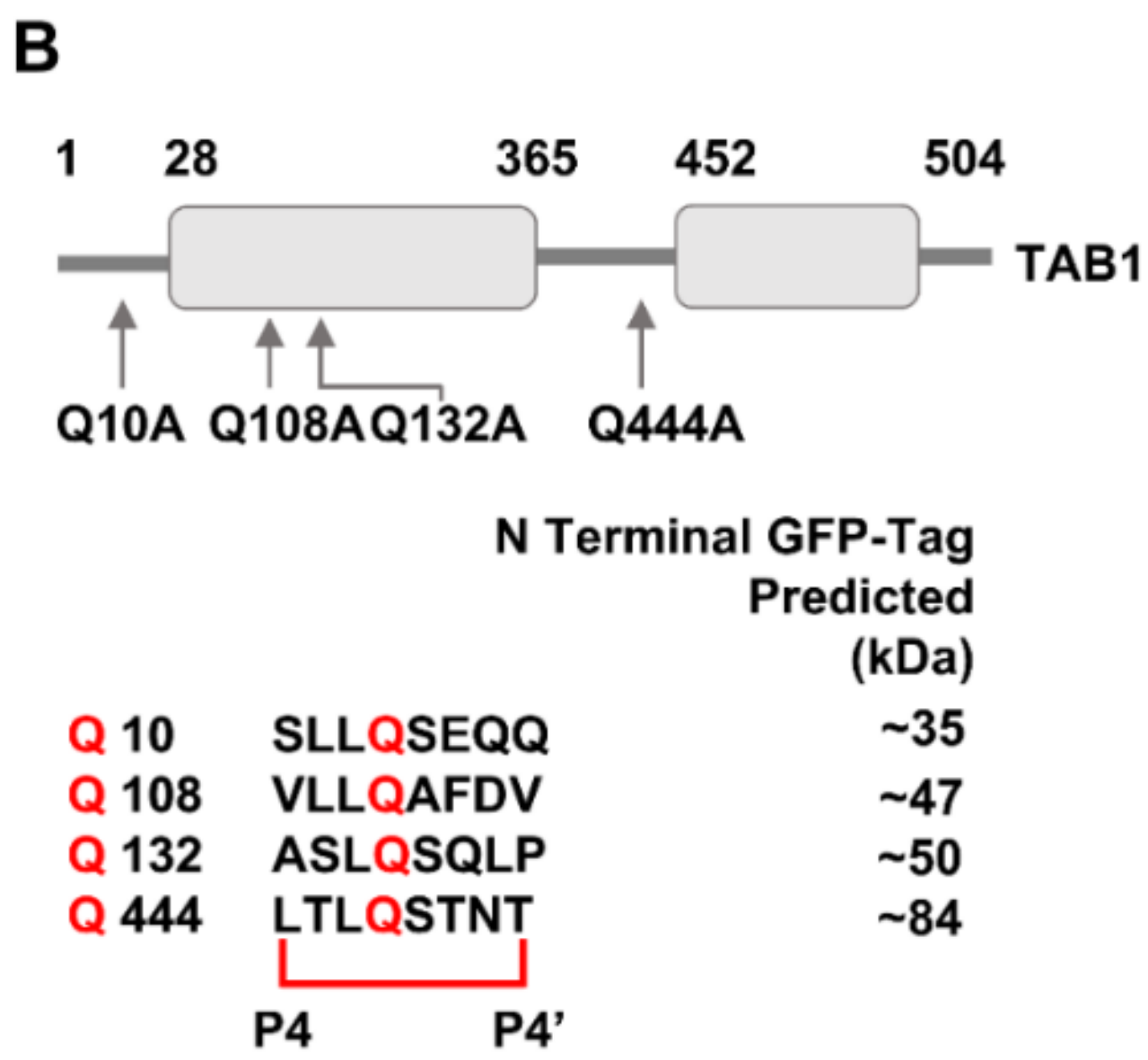

Fig. 2B: Domain architecture and cleavage site mutants of TAB1.

Comment 4: *If the single-site mutation in GFP-Mpro C145A affects its interaction and colocalization with TAB1 (Fig 1), does the Q-to-A mutant of TAB1 affect the interaction between TAB1 and Mpro? If such an impact exists, Mpro would not be able to cleave the TAB1 Q-A mutants under these circumstances, making it impossible to conclude that Q is a key cleavage site. Therefore, Fig 2 needs to supplement experiments to verify the binding between Mpro and the indicated Q-to-A mutants of TAB1.*

Response 4: We thank Reviewer 2 for this critical point. To verify this view, we additionally performed co-IP assays using the substrate-stabilizing mutant Mpro-C145A to verify the binding affinity of the TAB1 mutants (Q10A, Q444A, Q10A-Q444A). The new data (Fig.2E) reveal:

The Q10A mutant binds Mpro similarly to WT, whereas Q444A shows moderately reduced binding. The double mutant (Q10A/Q444A) exhibits significantly impaired interaction. These results confirm the reviewer 2's hypothesis that the P1 Glutamine (Q) residue is a critical determinant for Mpro substrate recognition. This is fully consistent with the canonical specificity of Mpro.

Crucially, this also validates our cleavage model, even with reduced binding (as seen in Q444A), Mpro still efficiently cleaves the remaining site (Q10), proving that the interaction remains sufficient for catalysis. The complete resistance of the double mutant Q10A-Q444A caused the loss of both bands and the disruption of the SARS-CoV-2 Mpro-TAB1 complex. We thank reviewer 2 for prompting this important clarification, which has strengthened the mechanistic depth of our study. In addition, we described the results in line 126-132 in the revised manuscript.

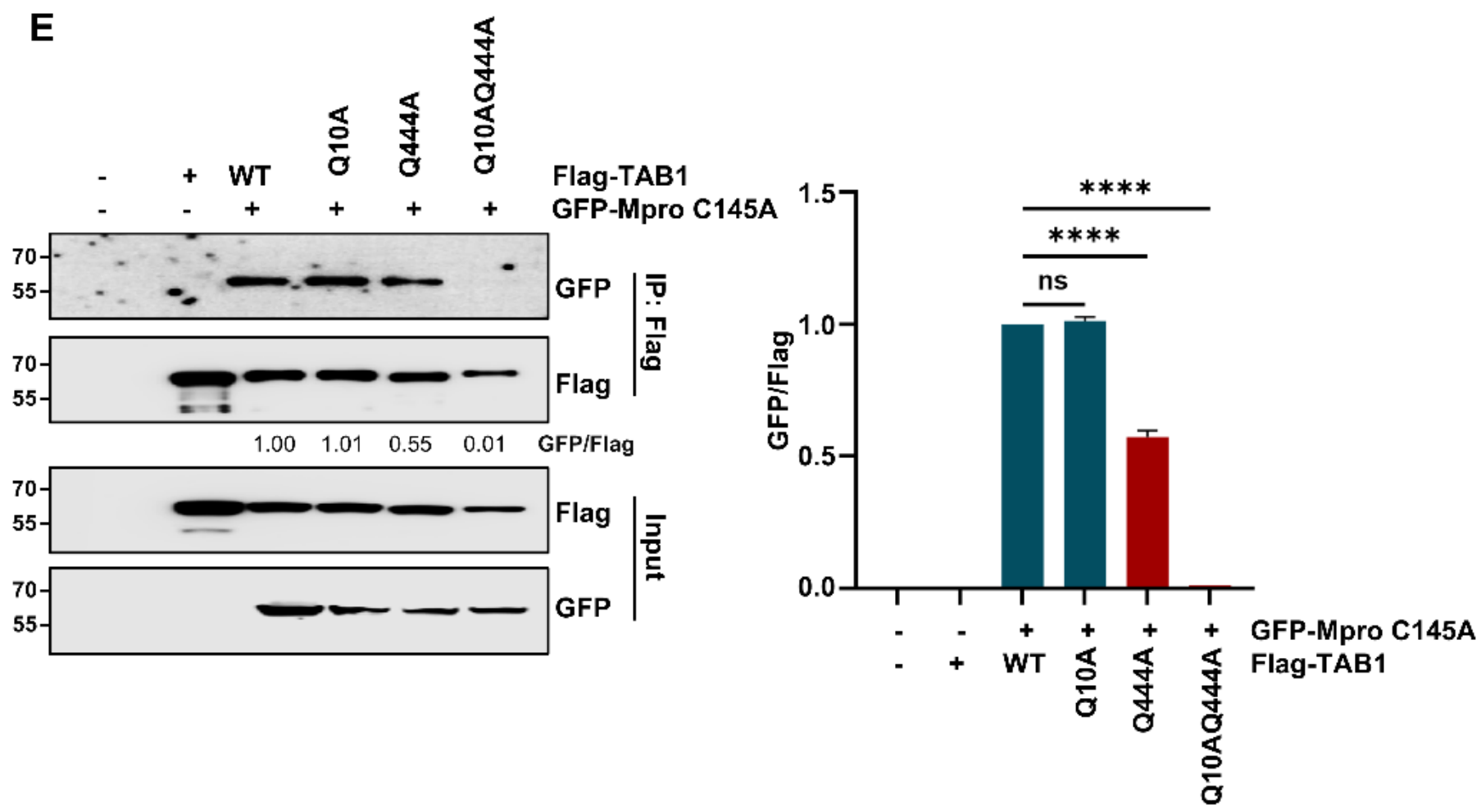

Fig. 2E: The binding of TAB (Q10A, Q444A and A10AQ444A) to Mpro C145A

Comment 5: *In FIG 3A-B, "NF-κB" refers to the reporter plasmid for NF-κB promoter, which differs in nature from TAB1/TAK1. This should be clearly labeled or unlabeled (it was unlabeled in FIG 3C-D), otherwise, it may cause confusion (For instance, it led me to mistakenly assume that the expression plasmids for NF-κB subunits were also co-transfected). Additionally, in the results presented by the authors, NF-κB signaling is not activated when TAB or TAK is overexpressed alone, but activated when co-expression. Are there similar literature reports to support this finding? Or are there other alternative ways to activate NF-κB?*

Response 5: We thank the reviewer for these important observations regarding the clarity of the figure presentation and the mechanistic rationale for the NF-κB activation assay.

(1) Clarification of Figure Labeling: The reviewer is correct that the label “NF-κB” in the original Figures 3A and 3B could be misinterpreted. It referred to the co-transfected NF-κB luciferase reporter plasmid, not to expression plasmids for NF-κB subunits. We have removed the standalone “NF-κB” label, making the presentation consistent with Figures 3C and 3D. The figure legend has been updated to explicitly state that “NF-κB activation” was measured using a luciferase reporter assay, clarifying the nature of the data.

(2) Mechanism of NF-κB Activation by TAB1/TAK1 Co-expression: The reviewer notes that in our system, NF-κB signaling is robustly activated only upon co-expression of TAK1 and TAB1, and not by either component alone. This observation is consistent with the established molecular mechanism of TAK1 activation. TAK1 is a kinase that requires upstream signals and binding partners for its full activation. TAB1 acts as a critical adaptor and activator that binds to TAK1, which are essential for downstream signaling, including NF-κB activation (PMID: 12372426; PMID: 10187861).

As suggested by the reviewer, we have included an additional control experiment to further validate our assay system. We treated cells with Tumor Necrosis Factor-alpha (TNF-α), a canonical and potent activator of the NF-κB pathway. As expected, TNF-α treatment led to strong NF-κB reporter activation. This result confirms the responsiveness and specificity of our reporter assay. The data for the TNF-α positive control has been added to Fig. S1A. This new experiment has been descried in line 142-144 in the revised manuscript.

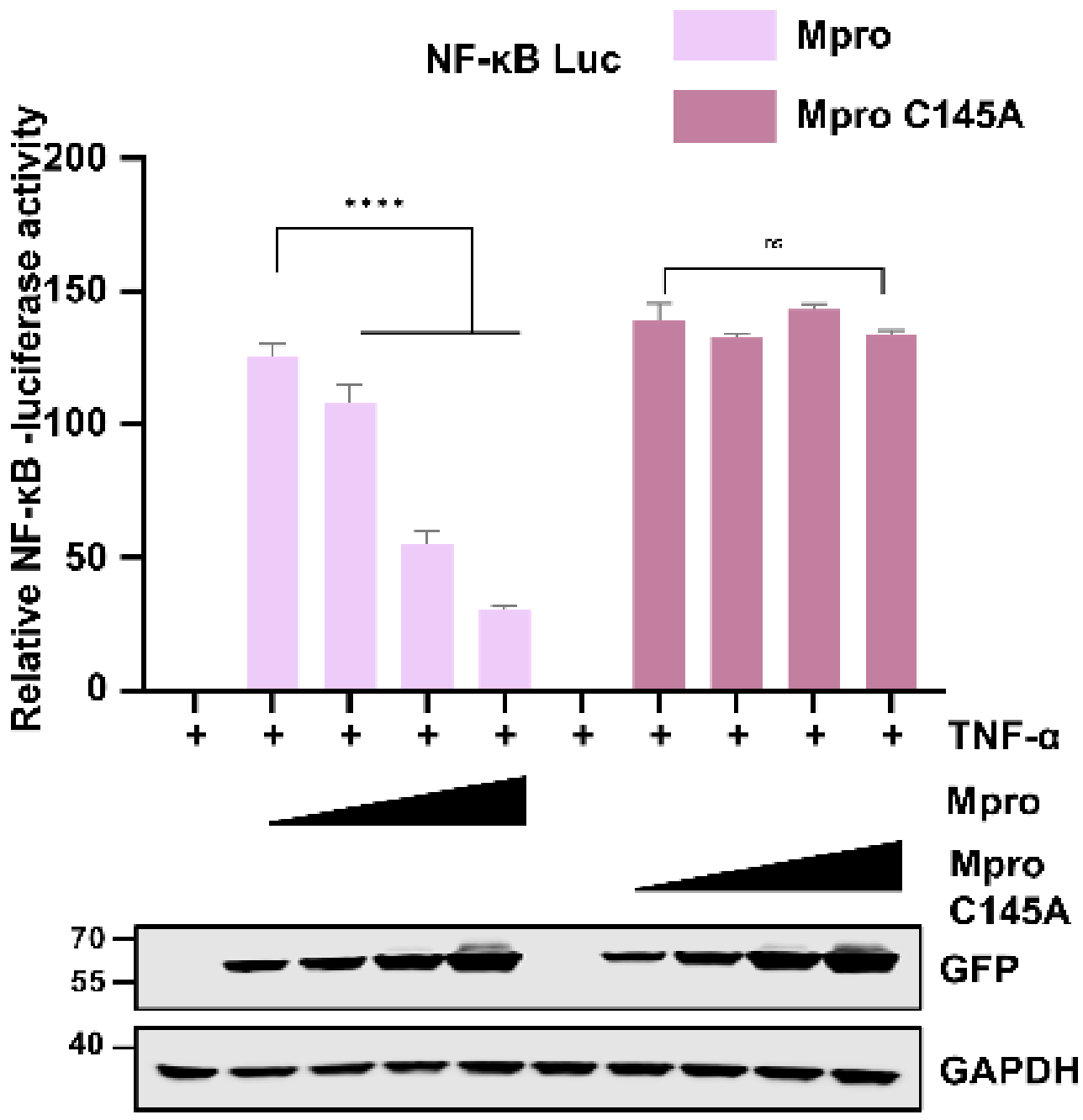

Fig.S1A: Mpro dose-dependently inhibits TNF-α induced NF-κB activation.

Comment 6: *Given that the cleavage of TAB1 by Mpro has been previously reported, and reporter gene assays may have certain inaccuracies, the authors should consider supplementing experiments to detect the inhibitory effect of Mpro expression on key events in the NF-κB signaling pathway (such as IκB phosphorylation and degradation, as well as p65 phosphorylation and nuclear translocation).*

Response 6: We thank the reviewer for this critical and constructive suggestion. In a direct response, we have performed a series of new experiments to assess the impact of Mpro on established hallmarks of canonical NF-κB activation.

We further investigated the key downstream effector p65 using cellular fractionation followed by Western blotting, we found that WT-Mpro expression inhibited the TNF-α-induced nuclear accumulation of p65, whereas the C145A mutant did not (Fig.S1B) . This new experiment has been descried in line 149-150 in the revised manuscript.

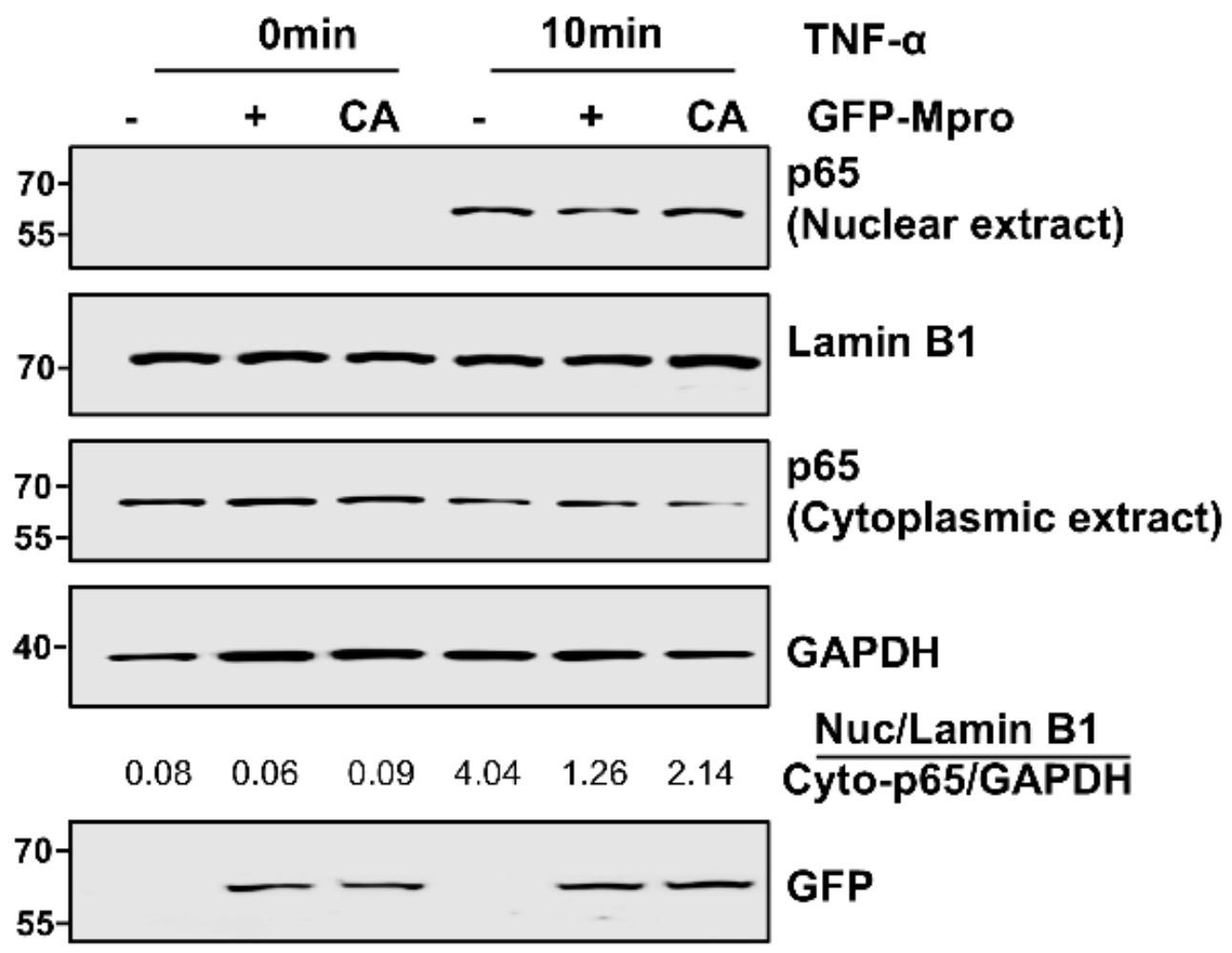

Fig.S1B: Mpro reduces the nuclear transfer of p65.

Therefore, we have based our conclusions on the robust and reproducible Western blot analyses of p65 nuclear translation, which are established and quantitative metrics of pathway inhibition.

Comment 7: *"P" value is missing in FIG 3B, and the labeling of statistical significance in the bar graphs throughout the manuscript is incorrect.*

Response 7: We thank the reviewer for pointing out the oversight regarding the statistical significance labels.

We have revised the figure legends throughout the manuscript to explicitly define the thresholds for statistical significance. **Data are presented as mean ± SD of three independent experiments, each performed in triplicate. Statistical significance was determined by one-way ANOVA and is indicated as follows: *P < 0.05, **P < 0.01, ***P < 0.001, ****P < 0.0001.*

Comment 8: *In the presence of Mpro, both termini of TAB1—including its N-terminal Flag tag—undergo cleavage. Consequently, when FLAG-TAB1 is used as the bait protein, the abundance of intact Flag-TAB1 is diminished as a direct result of Mpro-mediated cleavage; this reduction, in turn, lowers the likelihood of precipitating TAK1 (a TAB1-interacting protein). As such, the data cannot substantiate the conclusion that "Mpro markedly reduced TAB1–TAK1 binding".*

Response 8: We thank the reviewer for raising this critical point regarding the interpretation of our co-immunoprecipitation (co-IP) data on the TAB1-TAK1 interaction in the presence of Mpro. To verify this idea, we conducted the following new experiments.

Accordingly, we constructed and tested the individual TAB1 fragments generated by Mpro cleavage (1-10, 11-504, 1-444, and 445-504) for their ability to bind TAK1. The results in new Fig. 3E clearly demonstrate:

1) Full-length TAB1 (1-504) binds TAK1 efficiently, as expected.
2) The N-terminal fragments (1-10 and 1-444) show negligible binding to TAK1.
3) The C-terminal fragments (11-504 and 445-504) retain partial, albeit substantially reduced, binding for TAK1 compared to the full-length protein. This confirms that the primary TAK1-binding domain resides in the C-terminal region of TAB1.

Therefore, the observed decrease in TAK1 co-precipitation in the presence of Mpro can be explained by a combination of factors: Mpro cleavage converts intact FLAG-TAB1 into a mixture of fragments. Critically, the C-terminal fragments (11-504, 445-504), which retain the key TAK1-binding interface. Consequently, the signal reduction is not primarily caused by that Mpro directly occludes the TAB1-TAK1 binding interface, but rather caused by the cleavage drastically reduces the local concentration of precipitable, fully functional TAB1 bait, leading to an indirect decrease in detectable complex formation.

This new experiment has been described in line 161-164 in the revised manuscript.

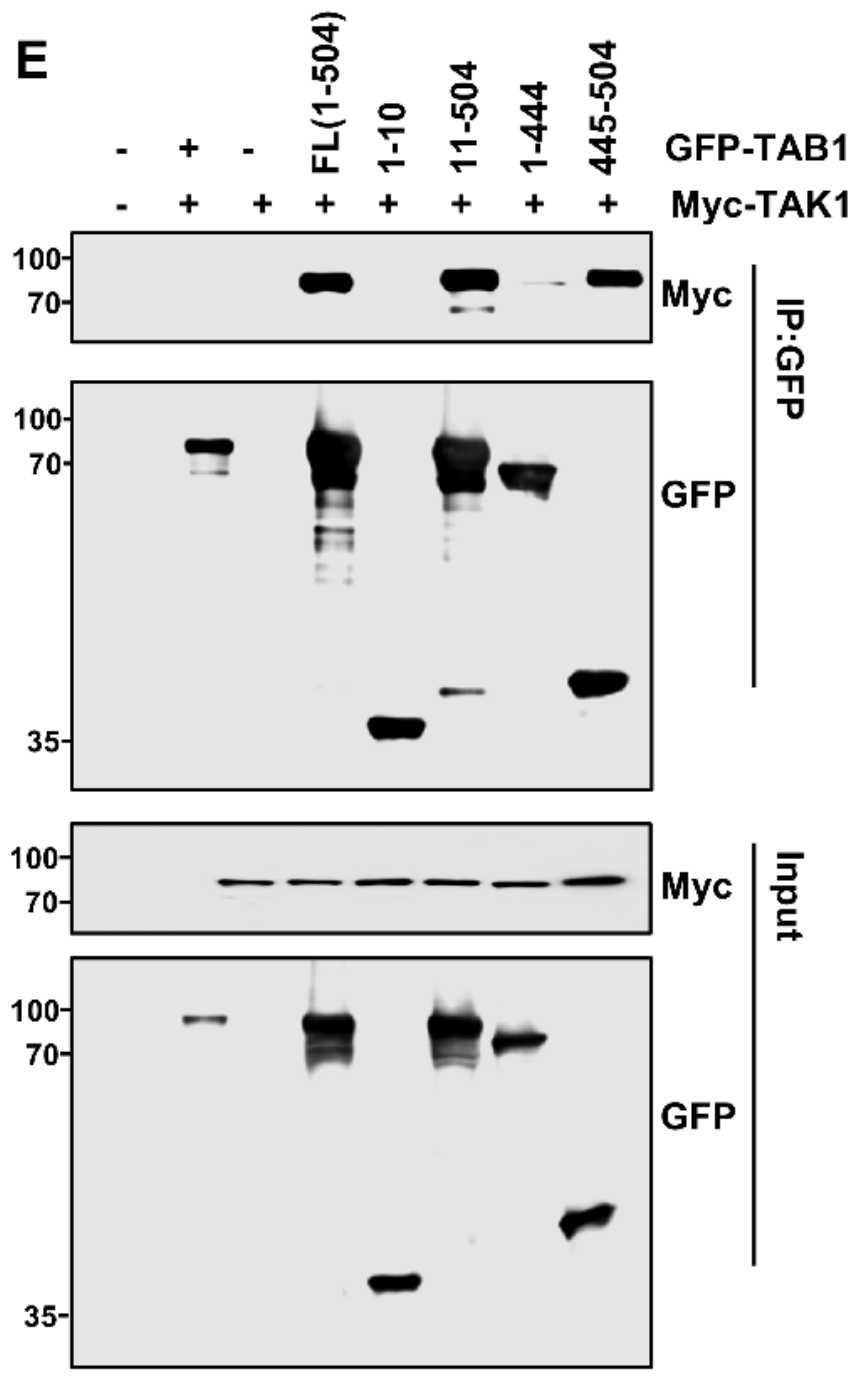

Fig. 3E: The binding of the truncated TAB1 to TAK1.

Comment 9: *Is there a statistical difference in the inhibitory effect on the NF-κB promoter between Mpro from low-pathogenic coronaviruses and that from high-pathogenic coronaviruses? Additionally, similar to the issue mentioned above, the presentation of statistical analysis in this figure is incorrect.*

Response 9: We thank the reviewer for this critical question and for identifying the error in statistical presentation, which we have corrected.

1. **Statistical comparison between Mpros from coronaviruses of differing pathogenicity**:

To address the reviewer's insightful question directly, we performed a post hoc analysis of the data from Figure 4G. We grouped the Mpro proteins accordingly (High-pathogenicity: SARS-CoV-2, SARS-CoV, and MERS-CoV; Low-pathogenicity: 229E, NL63, HKU1, and OC43) and conducted an unpaired t-test. This exploratory analysis indicated a trend toward greater NF-κB inhibition by the high-pathogenicity group, although the difference did not reach statistical significance under the current sample size ($p = 0.0571$). This result suggests a potential link worthy of further investigation. As such, throughout the revised manuscript, we have replaced the term 'high pathogenicity' with 'severe respiratory coronaviruses' (SARS-CoV-2, SARS-CoV,

and MERS-CoV). Similarly, we have replaced 'low pathogenicity' with 'seasonal human coronaviruses' (HCoV-OC43, HCoV-HKU1, HCoV-NL63, and HCoV-229E). We believe this nomenclature is more accurate.

Mann-Whitney test	
Table Analyzed	Data 2
Column B	Data Set-B
vs.	vs.
Column A	Data Set-A
**Mann Whitney test**	
P value	0.0571
Exact or approximate P value?	Exact
P value summary	ns
Significantly different (P < 0.05)?	No
One- or two-tailed P value?	Two-tailed
Sum of ranks in column A,B	6 , 22
Mann-Whitney U	0
**Difference between medians**	
Median of column A	20.92, n=3
Median of column B	56.64, n=4
Difference: Actual	35.72
Difference: Hodges-Lehmann	36.38

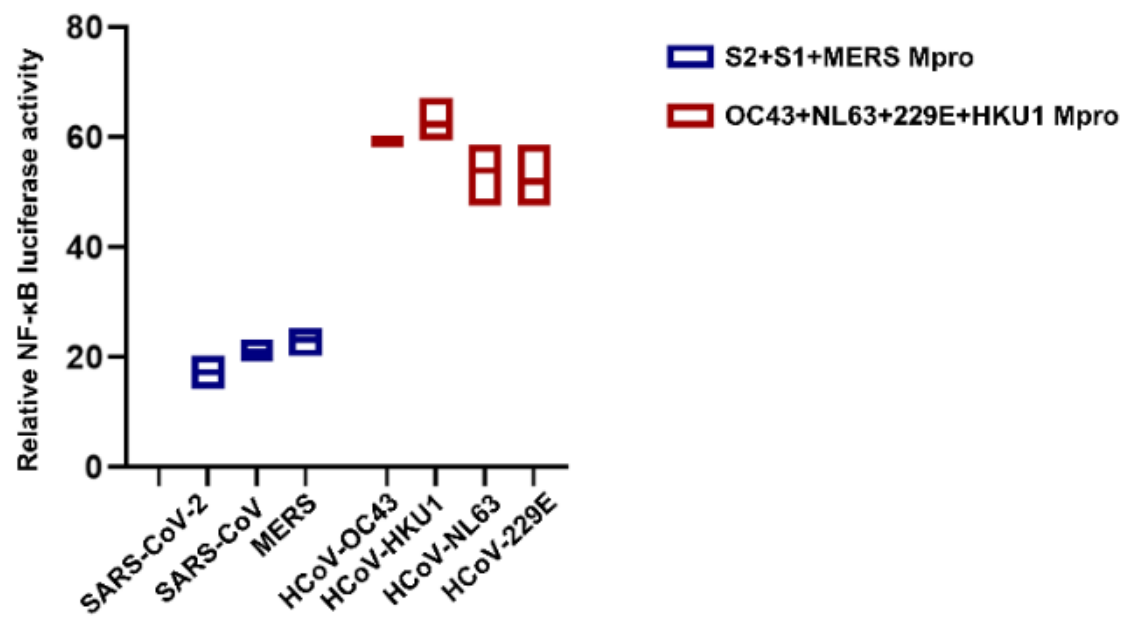

2. **Correction of statistical presentation**: This has been corrected. The revised legend now explicitly states **Data are presented as mean ± SD of three independent experiments, each performed in triplicate. Statistical significance was determined by one-way ANOVA and is indicated as follows: *P < 0.05, **P < 0.01, ***P < 0.001, ****P < 0.0001.*

Comment 10: *The substrate peptide (TAB1 residues 441-448) exhibits high affinity for SARS-CoV-2 Mpro. Can this peptide act as a competitive binder of Mpro to inhibit Mpro-mediated cleavage of TAB1, thereby suppressing SARS-CoV-2 infection?*

Response 10: We thank the reviewer for this insightful and forward-looking question.

Yes, our co-crystal structure (Fig. 5) provides a strong mechanistic basis for this possibility. We have commented on this in line 311-312 in the revised the manuscript.

Following a similar strategy of drug discovery, we previously developed a novel substrate-derived covalent peptidic inhibitor occupying the S1'-S3' substrate-binding pocket of SARS-CoV-2 Mpro (Liu et al., Angew Chem, 2023, PMID: 37609788).

Comment 11: *In line L316 of the Discussion, the claim is overstated. No data supporting a "complete shutdown of the NF-κB signaling pathway" was observed.*

Response 11: We thank the reviewer for this comment. We have therefore revised the text to more accurately reflect the level of suppression observed in our experiments. In line 290 in the revised manuscript: *Mpros from severe symptom coronaviruses cleave TAB1 with high efficiency at two sites, Q10 and Q444. The cleavage at Q444 is functionally decisive, as it disrupts the TAB1-TAK1 complex and potently inhibits NF-κB activation.*

Reviewer #3

In this manuscript, Fu et al. attempt to address the question of how CoV Mpros cleave the innate immune protein TAB1. They provide evidence that TAB1 is a conserved substrate for seven human coronavirus (HCoV) Mpros. Specifically, Mpros from SARS-CoV-2, SARS-CoV, and MERS cleave TAB1 at two distinct sites (Q10 and Q444), while Mpros from HCoV-OC43, HCoV-HKU1, HCoV-NL63, and HCoV-229E primarily cleave at Q10. This cleavage likely disrupts the TAB1-TAK1 complex, thereby blocking downstream signaling. This study builds on a previous report that demonstrated SARS-CoV-2 Mpro can cleave TAB1 at Q132 and Q444. Overall, this work offers novel and comprehensive insights that are useful to the field.

Response: We sincerely thank the Reviewer for positive and insightful evaluation of our manuscript.

Comment 1: *Line 169: "Interestingly, mutation of the Q10 site led the Mpro of HCoV-OC43, -229E, -NL63, and -HKU1 to adaptively cleave at the Q444 site, producing high-MW cleavage bands similar to those of Mpro from SARS-CoV-2, SARS-CoV, and MERS (Fig. 4C)." It is confusing why Q10 mutation causes Mpros from low-pathogenicity viruses to cleave at Q444 site as the authors previously claim they "selectively cleaved the Q10 site, yielding a single low-MW fragment (Fig. 4B) "in WT TAB1. The authors should repeat this experiment and provide a reasonable explanation for the observed discrepancy. To address this issue more effectively, I suggest the authors synthesize a fluorescent-labeled peptide derived from the Q10 region, test the enzyme activity, and compare the results with those obtained from Q444.*

Response 1: Thank you for raising this insightful and critical point. Your observation highlights a key mechanistic question regarding Mpro substrate preference and kinetic competition. We appreciate the opportunity to clarify this apparent discrepancy. Following reviewer 3's suggestion, we newly conducted enzymology experiment using a Q10-flurescent-labeled peptide (residues 7-14). With the newly obtained and our previous enzymology results, we believe that the results in Figures 4B and 4C are not contradictory but are, in fact, consistent and can be explained by the intrinsic kinetic competition between the two cleavage sites (Q10 and Q444) within the TAB1.

The differing cleavage patterns directly reflect the kinetic competition between the two sites, governed by the inherent catalytic efficiency (k_{cat}/K_m) of each Mpro variant for each site. In WT-TAB1, the Q10 and Q444 sites are both present and compete for cleavage by Mpro.

The data from enzymology experiments using fluorescent-labeled peptides corresponding to the Q10 region (residues 7-14) and the Q444 region (residues 441-448) provide a clear quantitative basis:

1) For the Q444-site peptide (Table 1): The Mpros from SARS-CoV-2, SARS-CoV, and MERS-CoV show high catalytic efficiency (k_{cat}/K_m within 0.92 -1.32 $\mu M^{-1}min^{-1}$). In contrast, the Mpros from HCoV-OC43, -229E, -NL63, and -HKU1 show markedly lower efficiency ($k_{cat}/K_m < 0.11$ $\mu M^{-1}min^{-1}$), a difference of over 8-fold.
2) For the Q10-site peptide (newly added Table 2): All seven Mpros exhibit catalytic efficiencies within a similar range (0.20–0.30 $\mu M^{-1}min^{-1}$). Notably, for the low-pathogenic HCoVs, their efficiency for the Q10 site (0.2-0.3 $\mu M^{-1}min^{-1}$) is slightly higher than their own efficiency for the Q444 site (0.05-0.08 $\mu M^{-1}min^{-1}$).
3) In WT-TAB1 (Fig. 4B): For severe symptom Mpros, the strong preference for Q444 drives the primary cleavage at this site, yielding the high-MW fragment. For seasonal strain Mpros, their relatively higher efficiency for Q10 makes it the dominant cleavage site, yielding the low-MW fragment. The very weak activity towards Q444 is effectively outcompeted and thus undetectable.
4) In Q10A-TAB1 mutant (Fig. 4C): Q10A mutation removes the preferred/dominant site for the seasonal strain Mpros. This alters the kinetic landscape. While their inherent activity on Q444 is low, with the competing Q10 site absent, this basal activity can accumulate over the reaction time and become detectable, producing the observed (likely weaker) high-MW band. The pattern for severe symptom Mpros remains unchanged because their primary target (Q444) is still present.

Thanks for this insightful question, we added the following explanation in the Results section in the revised manuscript (near Line 229-240):

'In contrast, all seven Mpros cleaved the Q10-site peptide with similar (Table 2, Fig.S6), low catalytic efficiencies, which clustered within a narrow range (~0.20–0.30 μM $^{-1}$s $^{-1}$) (Table 1). Notably, for the four seasonal strain Mpros, their efficiency for the Q10 site was slightly higher than their own efficiency for the Q444 site.

These kinetic data directly explain the cleavage patterns observed in cellular assays (Fig. 4B, 4C). For severe symptom Mpros, their strong kinetic preference for the Q444 site dictates exclusive cleavage at this position in WT-TAB1. For seasonal strain Mpros, their relatively higher efficiency for the Q10 site makes it the dominant cleavage target in WT-TAB1, while their weak basal activity at Q444 is kinetically outcompeted. In the Q10A-TAB1 mutant, removal of this dominant site allows the pre-existing, albeit low, activity of seasonal strain Mpros at the Q444 site to become detectable over the reaction time course, explaining the appearance of the high-MW fragment (Fig. 4C). '

Table 2. Kinetics of seven Mpro proteases measured with MCA-TAB1$_{7\text{-}14}$-Lys (DNP)

Proteases	k_{cat} (min^{-1})	K_m (μM)	K_{cat} / K_m (μM $^{-1}$ min $^{-1}$)
SARS-CoV 2 Mpro	43.14 ± 0.92	147.10 ± 14.29	0.29 ± 0.02
SARS-CoV Mpro	50.35 ± 4.77	169.80 ± 41.18	0.30 ± 0.04
MERS Mpro	21.87 ± 1.38	88.46 ± 20.33	0.25 ± 0.04
HCoV-HKU1 Mpro	47.65 ± 2.81	158.50 ± 18.13	0.30 ± 0.02
HCoV-OC43 Mpro	50.55 ± 1.31	165.50 ± 12.12	0.30 ± 0.01
HCoV-NL63 Mpro	48.24 ± 4.33	165.90 ± 12.82	0.29 ± 0.01
HCoV-229E Mpro	20.27±1.33	101.20±3.48	0.20± 0.01

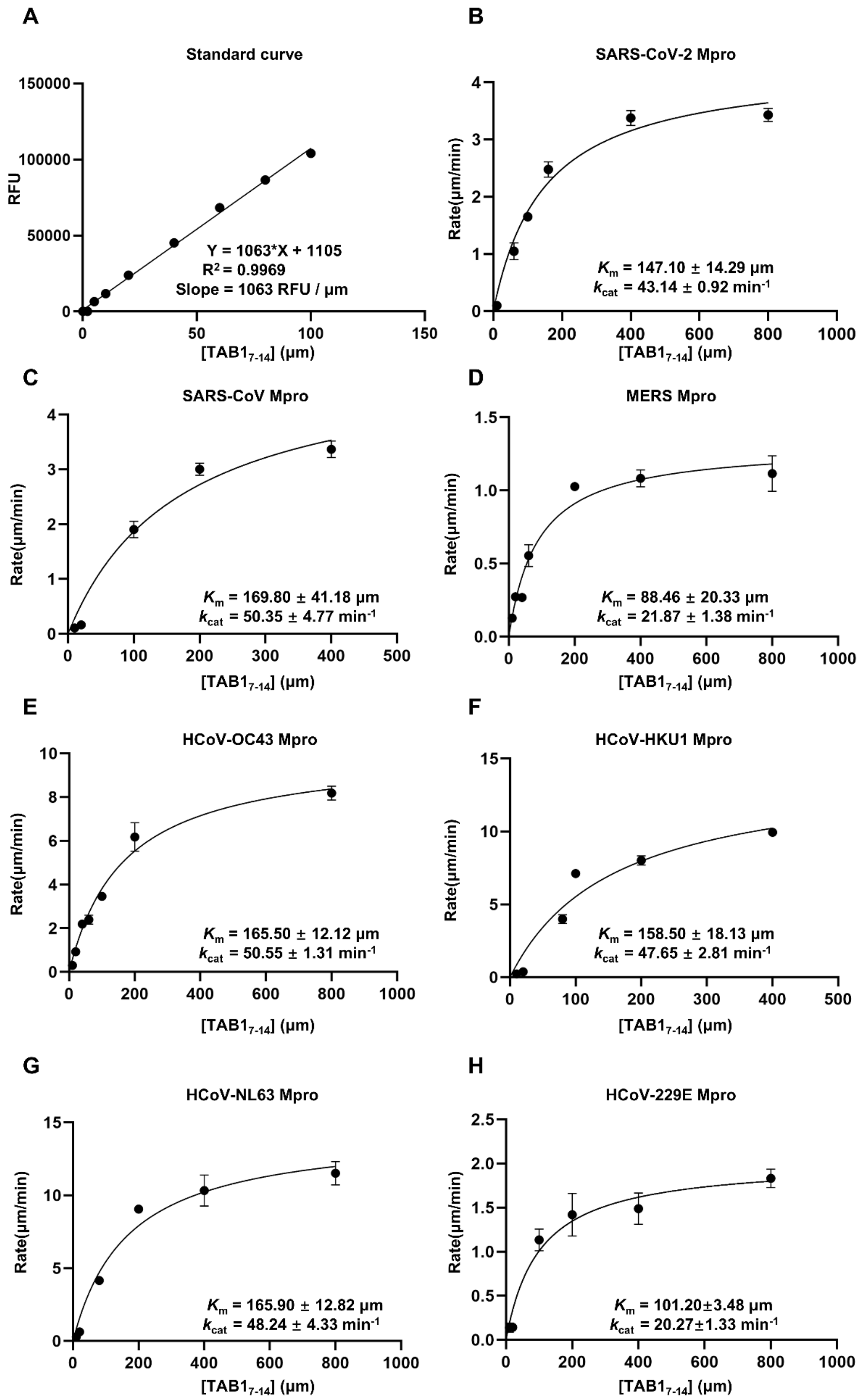

Comment 2: *The authors determined the structure of SARS-CoV-2 Mpro C145A in complex with human TAB1 (residues 441–448) at a resolution of 3.42 Å. In comparison with hundreds of high-resolution Mpro structures deposited in the PDB, this resolution is relatively low and may not provide sufficient detail for a thorough structural analysis. The authors should consider optimizing the crystals and improving the resolution to obtain more precise structural*

information.

Response 2: We sincerely thank the reviewers for raising this important point. We acknowledge that the resolution of the Mpro/TAB1 complex structure is lower than the high-resolution structures of Mpro in complex with small-molecule inhibitors reported in the field. In the revised manuscript, we will clearly describe this as a "medium-resolution" structure and will add a brief technical discussion regarding the resolution in the revised manuscript (line 242), as summarized below.

1. Possible Technical Reasons for the Resolution Limit. The peptide (TAB1$_{441-448}$, sequence LTLQSTNT) used for co-crystallization was designed based on the Mpro-binding fragment of TAB1. This peptide is highly hydrophobic and exhibits poor solubility in aqueous buffer. To enable crystallization, a low concentration of DMSO (<2%) was required to solubilize the peptide. **We note that DMSO could negatively impact the final diffraction quality**.

2. Considerations for Extended Peptide Screening. We have also evaluated the properties of longer peptide candidates. However, many of these also display near-neutral isoelectric points and similarly poor solubility, suggesting that reliance on DMSO for solubilization would likely present the same technical challenges without a clear path to significantly improved crystal quality.

TAB1	Peptide	Theoretical pI
441-448	LTLQSTNT	5.52
440-449	TLTLQSTNTH	6.40
439-450	PTLTLQSTNTHT	7.17
438-451	SPTLTLQSTNTHTQ	6.46
437-452	QSPTLTLQSTNTHTQS	6.74
436-453	NQSPTLTLQSTNTHTQSS	6.74
435-454	TNQSPTLTLQSTNTHTQSSS	6.40

3. Despite the moderate resolution, the primary goal of this structure was to unambiguously identify the TAB1 cleavage site on Mpro. This structural information has provided the direct and essential foundation for our subsequent mechanistic experiments, including site-directed mutagenesis, enzymatic kinetic analyses, and cellular functional assays. Thus, this structure has played a critical and specific role in defining the precise molecular interface between SARS-CoV-2 Mpro and TAB1.

Comment 3: *Given the low-resolution crystal structure, the discussion in the section titled "Comparison of the Mpro and TAB1 Structures of Seven Human Coronaviruses" is not convincing for detailed structural comparison.*

Response 3: We sincerely thank the reviewer for this comment. We agree that detailed structural comparisons are challenging given the medium resolution (3.43 Å) of the complex. In the revised manuscript, we have adjusted the discussion in this section to more explicitly acknowledge the resolution limits and to focus the comparison on the most reliable, conserved features. Following the reviewer's suggestion, and to keep the main text concise while preserving the information for interested readers, we moved this structural analysis section , along with its corresponding figure, to the Supplementary Materials, Fig. S6.

北京大學
PEKING UNIVERSITY

1299 LiShui Road F-401, Shenzhen University Town
Xili, Nanshan District, Shenzhen, Guangdong, China
Tel:+86-0755-2603-2321
Email: huang.hao@pku.edu.cn
http://web.pkusz.edu.cn/huanghao/

Manuscript ID: COMMSBIO-25-10053B

Title: The seven human coronavirus main proteases exhibit differential modulation of NF-κB suppression via TAB1 cleavage

Dear Editors and Reviewers,

We appreciate the opportunity to revise our manuscript and are grateful for the insightful comments provided by the editor and reviewers. In the following, we have provided point-by-point responses to each of the reviewers' comments. In the revised manuscript, we use red to indicate the traces of this revision.

Reviewer #1

The authors have responded very well to all of the previous comments and I do not see any reason that this can now not be accepted.

Response: Thank you very much for your time in reviewing our revised manuscript and for your final positive assessment.

I make two minor points:

1. While I appreciate that they do not have an antibody to Mpro - they do exist, so I feel the Ponceau stain in figure 1 could be replaced.

Response 1: Following your suggestion, we have removed the Ponceau-stained bands in the modified Figure 1.

2. The authors state that the Ponceau shows equal loading - however, there is clearly less protein present in the mutant version than the wild-type in figure 1D. However,

given all of the other evidence of the interaction they propose - these are minor and not essential to fix. I would almost be tempted to remove the Ponceau stain - though I do appreciate the need for it.

Response 2: We apologize for the previous insufficient precision in our description. Following reviewer's suggestion, we have removed the data of Ponceau-stained gel in the revised manuscript.

Reviewer #2:

Regarding the revised version, I consider it necessary to repeat some of the Mpro–TAB1 interaction data. Moreover, given that TAB1 antibodies are commercially available, it would be advisable to detect TAB1 using antibodies against the protein (polyclonal antibodies recognizing multiple epitopes might be more appropriate) rather than relying on tag antibodies, given that the N terminal tag is cleaved off.

Response: Thank you very much for reviewing our revised draft again and for providing these important suggestions. Regarding the interaction between Mpro and TAB1, in the IP experiment (Fig. 1A), lane 4 shows that the enzymatically inactive Mpro C145A cannot cleave TAB1, with no cleavage bands observed, but a clear interaction is present. In confocal microscopy (Fig. 1B), Mpro C145A also interacts with TAB1, showing a Pearson coefficient of 0.73. These data support the interaction between Mpro and TAB1. Most importantly, we have solved the co-crystal structure of Mpro in complex with the TAB1 peptide in this study, which serves as the gold standard for validating direct protein-protein interactions. Taken together, our integrated biochemical, cellular, and structural analyses provide compelling evidence for the Mpro-TAB1 interaction.

Specific comments:

1 Considering the transient nature of many enzyme-substrate interactions, the authors' rebuttal could explain why uncleaved FLAG-TAB1 fails to pull down Mpro. However, I

still have concerns regarding Figure 1A. First, why are cleaved TAB1 fragments detected by the FLAG antibody (two bands) in the input samples, but only the full-length form (a single band) remains in the pull-down (PD) samples? Second, there is a serious inconsistency between the protein amounts in the input and the pull-down: the group with the highest input shows the least amount pulled down. Furthermore, comparable—if not higher—levels of full-length TAB1 were enriched in the wild-type group (where TAB1 cleavage occurs) compared to the mutant group (where no cleavage occurs).

Response 1: Thank you for these comments. In Fig. 1A, WT Mpro cleaves substrate TAB1, resulting in a decrease in the total amount of full-length TAB1 (Input) within the cells. However, the IP experiment captures the interaction complexes at the moment of lysis. Although most of the TAB1 has been cleaved, some full-length TAB1 is still binding and being enriched, so the PD band can still be detected. In contrast, the C145A mutant stably binds and protects TAB1 from degradation, leading to a greater accumulation of full-length TAB1 in the Input. This contrast instead proves the cleavage activity of WT Mpro on its substrate TAB1.

The main text modification (line 97):"Notably, in the input samples, the Flag antibody detected both full-length (FL-) TAB1 and a cleaved fragment (Fig. 1A), confirming the proteolytic activity of WT-Mpro. These phenomena collectively indicate that the interaction between the wild-type Mpro and its substrate TAB1 is transient and catalytically dependent: Mpro dissociates rapidly from the product after completing the catalytic cleavage, making it difficult for its own molecule to remain in the immunoprecipitation complex. The residual full-length Flag-TAB1 in the immunoprecipitate may represent the intact substrate fraction. This result qualitatively proves the direct interaction between Mpro and TAB1 and suggests that its catalytic activity significantly affects the stability of the complex.

2 Considering that the N-terminal FLAG tag on FLAG-TAB1 is cleaved by Mpro, using a FLAG antibody to represent TAB1 localization in Figure 1C is also inaccurate. Moreover, given the transient nature of the enzyme-substrate intermediate complex,

live-cell dynamic imaging might be more suitable than imaging fixed samples.

Response 2: We are grateful for the important insights provided by the reviewer. The immunofluorescence confocal experiment is shown in Fig. 1B, not Fig. 1C. Due to the current equipment limitations, the suggested experiment could not be conducted in this study. Alternatively, we presented the data on the catalytic inactive Mpro-C145A interacting with Flag-TAB1 (the bottom row in Fig. 1B), where the Flag-tag was not cleaved. Thus, our data on WT-Mpro/TAB1 and Mpro-C145A/TAB1 provided consistent localization information.

3 In Figure 2E, the Q10A mutation does not show a substantial reduction in the Mpro-TAB1 interaction. This contradicts the authors' description: "Mutation of either Q10 or Q444 significantly weakened the binding affinity between TAB1 and Mpro C145A. Moreover, the Q10A/Q444A double mutant failed to co-immunoprecipitate with Mpro C145A, indicating a complete loss of binding (Fig. 2E). Collectively, these data establish Q10 and Q444 as critical residues on TAB1 for both recognition and cleavage by SARS-CoV-2 Mpro."

Response 3: Thank you for the comment. We agree that the general description of "significantly weakened" in the original text failed to accurately reflect the difference in the degree of binding reduction between the Q10A and Q444A mutants in Figure 2E. We have accordingly revised the main text to clearly distinguish the phenotypes of the two: “the Q10A mutant retains binding, while the Q444A mutant shows a more significant reduction in binding.” (line 136) These revisions ensure that the textual description is fully consistent with the data in the figures.

4 In Figure 3F, the use of an N-terminally tagged TAB1 (which is cleaved by Mpro) likely overestimates the effect of Mpro cleavage at the TAB1 C-terminus on its binding to TAK1. Although the authors touched on this point in the rebuttal, it is not reflected in the main text. Perhaps an alternative approach would be to insert the tag after the Q10 site, or to use Q10 mutants, to exclude the influence of TAB1 cleavage at the Q10

site on this experiment.

Response 4: We sincerely apologize for omitting this important discussion from the main text in our previous revision. We agree with the reviewer that the cleavage at the N-terminal Q10 site separates the N-terminal tag from the main body of TAB1, which technically limits the detection of the remaining fragments in our Co-IP assay and may overestimate the reduction in TAK1 binding.

However, as we demonstrated in Figure 3E, the far C-terminal region of TAB1 (445-504) is absolutely required for its interaction with TAK1. Therefore, even if we were able to immune-precipitate the fragments (e.g., 1-10 or 1-444), they would still fail to bind TAK1 due to the loss of the C-terminal binding domain.

Following your highly constructive suggestion, we have now explicitly acknowledged this caveat and incorporated this rationale into the main text to ensure scientific rigor. (Line 181: "It is worth noting that the use of an N-terminally tagged TAB1 may overestimate the reduction in TAK1 binding due to the concurrent cleavage at the Q10 site. However, as demonstrated in Figure 3E, the C-terminal region of TAB1 (445-504) is essential for TAK1 binding. Thus, the loss of the C-terminus by Mpro cleavage is sufficient to abolish the TAB1-TAK1 interaction entirely, regardless of the tag separation at the N-terminus."

Reviewer #3:

The authors have addressed the reviewer's concerns.

Response: Thank you for reviewing our revised manuscript and providing the final evaluation.
